# Supplementary material for: Measuring supply-side service disruption: a systematic review of the methods for measuring disruption in the context of maternal and newborn health services in low and middle-income settings
Source: BMJ Open. 2023 Dec 9;13(12):e077583. doi: 10.1136/bmjopen-2023-077583 (PMC10729240; doi:10.1136/bmjopen-2023-077583)
Supplement: Supplementary data [file bmjopen-2023-077583supp001.pdf]

APPENDIX 1: Search Strategy

Database	MNH	Service	COVID-19
MEDLINE	maternal OR pre?natal OR peri?natal or peri?partum OR ante?natal OR post?natal OR postpartum OR pregnan* OR breastf* OR babo?r OR midwife* OR "birth attendant*" OR "attended birth*" OR new?born [TI/AB]<br><br>maternal health services [MeSH]	service OR program* [TI/AB]	COVID-19 OR 2019-nCoV OR SARS-CoV-2 OR coronavirus OR COVID OR COVID19 [TI/AB]<br><br>covid-19 [MeSH]
EMBASE	maternal OR pre?natal OR peri?natal or peri?partum OR ante?natal OR post?natal OR postpartum OR pregnan* OR breastf* OR babo?r OR midwife* OR "birth attendant*" OR "attended birth*" OR new?born [TI/AB]	service OR program* [TI/AB]	COVID-19 OR 2019-nCoV OR SARS-CoV-2 OR coronavirus OR COVID OR COVID19 [TI/AB]
Global Health	maternal OR pre?natal OR peri?natal or peri?partum OR ante?natal OR post?natal OR postpartum OR pregnan* OR breastf* OR babo?r OR midwife* OR "birth attendant*" OR "attended birth*" OR new?born [TI/AB]	service OR program* [TI/AB]	COVID-19 OR 2019-nCoV OR SARS-CoV-2 OR coronavirus OR COVID OR COVID19 [TI/AB]

McGowan CR, *et al. BMJ Open* 2023; 13:e077583. doi: 10.1136/bmjopen-2023-077583

APPENDIX 2: INCLUDED STUDIES

AUTHOR	YEAR	AIM	METHOD	SETTING	DATA COLLECTED	FOCUS	FINDINGS
Adelekan (40)	2021	"…to investigate the extent to which the COVID-19 pandemic and related lockdowns had affected the provision of essential reproductive, maternal, child, and adolescent health (RMCAH) services in primary health care facilities across the Nigerian States" (p. 01).	**QUALITATIVE**<br>Semi-structured interviews (questionnaire not provided)<br>Description of questions: description of the health facility, RMCAH service delivery and use, and difficulties experienced before/during/after lockdown.<br>Head nurses and midwives	**NIGERIA**<br>Primary Health Centres (PHCs) in selected Local Government Areas (LGAs) in 10 states: Lagos, Akwa Ibom, Kano, Kaduna, Gombe, Borno, Ogun, Enugu, Adamawa, and the Federal Capital Territory (FCT) (Abuja Municipal Area Council)	**2020**<br>1 Nov-16 Dec	**RMCAH**	**PROVISION OF SERVICE**<br>**Defined as: Percentage of surveyed PHC's offering a service**<br>ANC: No decline in provision of service except in Gombe (10% decrease during lockdown)<br>Delivery care: No decline in provision of service.<br>Intrapartum care: No decline in provision of service except in Gombe (increased during lockdown but following lockdown dropped to pre-lockdown levels).<br>Postnatal care: Decline in provision of service during lockdown (for some states) and further decline following lockdown (for some states).<br>Childhood immunisation: Decline during lockdown and further decline following lockdown (except in Borno)
Akaba (39)	2022	"…to explore the barriers and facilitators of access to MNCH services during the first wave of COVID-19	**QUALITATIVE**<br>In-depth interviews (questionnaire provided)<br>Used 'three delays model' to structure interviews (i.e. reason for delay in seeking	**NIGERIA**<br>Six states: Abuja, Lagos, Kano (high numbers of cases); Engu, Taraba,	**2020**<br>May/Jul	**MNH**	**QUALITY OF SERVICE**<br>• Delays in receiving care<br>• Shortage of health staff<br>• Inadequate supplies<br>• Lack of preparedness amongst staff

		pandemic in Nigeria" (p. 01).	care, delay in reaching health care facility, delay in receiving care at the hospital).<br>Service users, health workers, policymakers	Bayelsa (lower numbers of cases)			
Asefa (47)	2022	"…to explore how the COVID-19 pandemic negatively affected frontline health workers' ability to provide respectful maternity care globally" (p. 378).	**QUALITATIVE**<br>Survey data [free-text responses] (questionnaire not provided)<br>Description of question: '*At this time, to what extent do you feel that you are able to provide respectful care to women and newborns compared to before the COVID-19 outbreak?*'<br>Health workers (mainly midwives, obstetricians, gynaecologists, nurses, and GPs)	**71 COUNTRIES**<br>Includes LMICs and HICs	**2020**<br>Jul-Dec	**Respectful maternity care**	**QUALITY OF SERVICE**<br>Six ways in which the provision of care was negatively affected:<br>• Less family involvement<br>• Reduced emotional support to women<br>• Reduced physical support for women<br>• Compromised standards of care<br>• Increased risk of medically unjustified caesarean section<br>• Overwhelmed staff with rapidly changing guidelines and enhanced infection prevention measures
Ashish (37)	2021	"…to understand the disruption of maternity services in Nepal due to the COVID-19 pandemic as well as to explore the provision and experience of institutional delivery care" (p. 2).	**BOTH**<br>**Quantitative**<br>Observation [number of health care workers in the labour and delivery room and those re-deployed to COVID-19 area; number of days maternity services were disrupted]	**NEPAL**	2019/20<br>Mar-Aug 2019, Mar-Aug 2020	**Respectful maternity care**	**PROVISION OF SERVICE**<br>• Maternity services halted for mean 4.3 days during pandemic<br>• Redeployment of staff to COVID-19 care<br>• Positive<br>• Reduced number of health care workers in labour room (compared to pre-COVID)

			**Qualitative**<br>Semi-structured interviews (questionnaire not provided)<br>Mothers with live births (followed-up 45 days post-partum)				**QUALITY OF SERVICE**<br>• Less than half were counselled to keep newborn warm (40%), or exclusive breastfeeding (44%).<br>• Few were counselled on newborn danger signs (15%)<br>• Consent before vaginal examination (primiparous 55%; nulliparous 44%)
Assefa (13)	2021	"…to characterize the impacts of the COVID-19 pandemic on the interruptions on health services from the perspectives of both HCPs and community members in three sub-Saharan African countries, Burkina Faso, Ethiopia, and Nigeria" (p. 324).	**QUANTITATIVE**<br>Survey data [interruption of health service by type] (questionnaire not included)<br>Questions related to the interruption of a type of health service were scored from 0 to 2 (0 = no interruption;1 = partial interruption; 2 = complete interruption). Asked to score: ANC, iron and folate, family planning.<br>Health care workers (also surveyed community to assess access – not included)	**BURKINA FASO, ETHIOPIA, NIGERIA**	**2020**<br>Jul-Nov	**General (but includes maternal and reproductive health services)**	**PROVISION OF SERVICE**<br>• Almost all services were reported to have experienced disruption<br>• Maternal and reproductive services particularly interrupted<br>• MRH: Nigeria (2.24 out of 6); Burkina Faso (1.72 out of 6); and Ethiopia (1.67 out of 6). [where 6=complete interruption]. Total (1.85 out of 6).<br>• Total health service interruption: Nigeria (7.9 out of 20); Burkina Faso (6.2 out of 20); and Ethiopia (5.4 out of 20). [where 20=complete interruption]. Total (6.49 out of 20)
Avula (27)	2022	to, "…stud[y] disruptions and restorations, challenges and adaptations in health and nutrition service delivery by frontline workers (FLWs) in India during	**QUANTITATIVE**<br>Survey data [service delivery at T1-lockdown and T2-post-lockdown] (questionnaire included)<br>Question example:	**INDIA**<br>Seven states: Bihar, Chhattisgarh, Madya Pradesh, Odisha, Tamil Nadu, Telangana, and Uttar Pradesh	**2020**<br>Aug-Oct	**FLW-provided services (iron and folic acid, and ANC)**	**PROVISION OF SERVICE**<br>• Services were disrupted during T1-lockdown but were restored in the T2-post lockdown (but not to T0-pre-pandemic levels)

		COVID-19 in 2020" (p. 1).	a. "Were you able to provide help in providing antenatal care services to all pregnant women?" (Yes/No)<br>b. "…in the last month?" (0=No one; 1=Yes, only to some pregnant women in the last trimester for high-risk pregnant women; 2=Yes, to all pregnant women)<br>c. "…during the last COVID-19 lockdown in April?"<br>HIS data also retrieved to establish T0-pre-COVID-19 service provision.<br>Frontline workers				
Basnet (38)	2022	"…to explore the experiences of nurses providing maternity care in the public sector during the COVID-19 pandemic in Nepal" (p. 01).	**QUALITATIVE**<br>Semi-structured interviews (topic guide not provided)<br>Topic guide was developed based on previous relevant literature, the research question, and informal interviews.<br>Nurses	**NEPAL**<br>Biratnagar (Koshi Hospital)	**2020**<br>Sep-Nov	**MNH**	**QUALITY OF SERVICE**<br>• The majority of the 10 participants had experienced decreased quality of care<br>• Decrease in nurse-patient time<br>• Decrease in presence of medical practitioners<br>• Time allocated per patient decreased<br>**PROVISION OF SERVICE**<br>• Reduced kangaroo mother care for pre-term<br>• Counselling less consistent
Hazfiarini (33)	2022	"…[t]o explore how COVID-19 influenced the provision of high-	**QUALITATIVE**<br>Semi-structured interviews (topic guide not provided)	**INDONESIA**<br>Surabaya and Mataram	**2020/1**	**Midwife services**	**PROVISION OF SERVICE**<br>• Cancelled education classes

		quality maternity care in Indonesia" (p. 01).	Topic guide included three sections: midwives were asked how they provided antenatal, childbirth, postpartum care prior to COVID-19 [Q1] and during COVID-19 [Q2], and were asked to describe experiences providing care during COVID-19 [Q3].<br>Midwives		Dec 2020 - Feb 2021		• Reduced frequency of ANC reduced (reducing from 6 to 2-4)<br>• Reduced post-partum visits
Leung (41)	2022	"…to explore maternity healthcare workers' experiences of, and perceptions about providing maternity care during the COVID-19 outbreak in Lagos State, Nigeria" (p. 01).	**QUALITATIVE**<br>Semi-structured interviews (topic guide provided)<br>Topic guide included:<br>"How has COVID-19 outbreak affected maternity care services?<br>What impact do you feel that COVID-19 pandemic has had on your work?<br>Probe: How has COVID-19 affected normal daily tasks/responsibilities?<br>Probe: How has COVID-19 affected the way you look after patients?<br>Probe: Have supply of drugs, equipment, PPE been affected?	**NIGERIA**<br>Seven Local Government Areas in Lagos State (Lagos Mainland, Surulere, Ikeja, Alimosho, Kosofe, Badagry, OshodiIsolo)	**2021**<br>Mar-Apr	**Facility-based maternity care**	**PROVISION OF SERVICE**<br>• Antenatal appointments for non-urgent cases had been reduced<br>• Home visits not possible<br>• Some felt services maintained<br>**QUALITY OF SERVICE**<br>• Some midwives had taken on COVID-19 roles (e.g. vaccination)<br>• Reduced capacity of consultation room

			Probe: Have staff been redeployed from or within your facility?<br>Please tell me about any changes COVID-19 has had on your work<br>Probe: Can you explain why there have been these changes?<br>Probe: How do you feel about the changes you have had to make during the pandemic?<br>Probe: COVID-19 control measures - How do you feel about mask wearing/social distancing/handwashing?<br>What differences have you noticed in the way that patients interact with your services during COVID-19?<br>Probe: Has there been a change? Why?<br>Midwives and TBAs				
Mirzakhani (35)	2022	"…to explore the experiences of women with high-risk pregnancies who were receiving prenatal care during the COVID-19 pandemic" (p. 01).	**QUALITATIVE**<br>Semi-structured interviews (no topic guide provided)<br>Topic guide included:<br>1. What was your experience with prenatal care during the Corona pandemic?	**IRAN**<br>Ghaem, Ommolbanin, and Imam Reza in Mashhad	**2020/2**<br><br>Sep 2020 – Mar 2021	**Women with high-risk pregnancies**	**QUALITY OF SERVICE**<br>• Health care provider's care and attention had decreased during COVID-19<br>• Staff refused to listen to foetal sounds or perform abdominal examinations<br>• Shorter visits from health care workers

			2. How do you feel about being pregnant in this situation?<br>3. How do you feel when you apply for prenatal care?<br>4. How is your access to prenatal care in the face of the Corona pandemic?<br>5. Discuss the factors that make you feel good about receiving health services.<br>6. Discuss the factors that make you feel uneasy/bad about receiving health services.<br>7. How does the environment and location of health delivery affect health services receipt?<br>8. What limitations /restrictions did you face when seeking health services?<br>Women with high-risk pregnancies				• Staff not taking time to talk<br>• Mothers reported stress, anxiety, neglect<br>**PROVISION OF SERVICE**<br>• Some doctors closed their offices<br>• Drug shortages
Mohammed (42)	2022	"…[to] assess the effect of Covid-19 on maternal and child health services in Federal medical Centre Gusau Zamfara state following the lockdown" (p. 124).	**QUALITATIVE**<br>Key informant interviews (no topic guide provided)<br>Topic guide was 'standard COVID-19 KII guide' and asked respondents' opinion on the COVID-19 outbreak	**NIGERIA**<br>Federal medical centre Gusau, Zamfara state	??	**MNH services**	**PROVISION OF SERVICE**<br>• Some respondents reported having to quarantine after contact with COVID-19 positive patient<br>• Most participants believed services were affected with some suspended

			and its specific and general effects on the facility. Heads of units delivering MNCH services (principal medical officers, chief nursing officers, principal nursing officers and nutrition officers)				**QUALITY OF SERVICE** • Paediatric outpatient and paediatric emergency unit reduced staffing numbers
Musiimenta (43)	2022	"…[to] describe the challenges experienced in accessing maternal and child health services by women with limited or no education during this COVID-19 pandemic and discuss the potential of social network technologies (SNTs) to support maternal and child health amidst this crisis".	**QUALITATIVE** Interviews (no topic guide provided) Interviews meant to elicit information about challenges women encountering in accessing maternal and child health services during lockdown. Women who had recently given birth in a rural setting	**UGANDA** Mbarara Regional Referral Hospital	**2021** Jul	**Post-natal care**	**PROVISION OF SERVICE** • Delays in accessing services, long queues and missed appointments
Nakate (44)	2022	"…[to] explore the experiences of mothers and their significant others of comprehensive care in the first 1000 days of life post-conception during the COVID-19 pandemic" (p. 01).	**QUALITATIVE** Semi-structured interviews (no topic guide provided) Topics included: Open-ended questions regarding participants' experiences accessing care and their understanding of these experiences.	**UGANDA** Bunghokho-Mutoto sub-county, Mbale District	**2020** 10 Oct-9 Nov	**First 1000 days post-conception**	**PROVISION OF SERVICE** • Lack of follow-up visits • Curfew resulted in staff starting late and leaving early whilst mothers waited • Facilities reduced admissions or appointments due to staff shortages **QUALITY OF SERVICE**

			Mothers (pregnant or with a child under 2 years) and their significant others				• Shortages of staff and work overload was felt to have hindered provision of care<br>• Staff pre-occupied with infection prevention and control
Nguyen (24)	2021	"…to (1) quantify the effect of the COVID-19 pandemic and COVID-19 restrictions on health and nutrition service provision and utilisation by pregnant women and mothers of children under 2 years of age in Dhaka City, Bangladesh, and (2) identify adaptions and a range of feasible solutions that have the potential to strengthen delivery and uptake of essential health and nutrition interventions in the context of COVID-19 and beyond" (p. 03).	**QUANTITATIVE**<br>Survey data [to assess survey provision before pandemic, during lockdown, and post-lockdown]<br>(survey not included)<br>Survey (health providers):<br>1) Feb baseline: assess exposure to nutrition training, workload and time commitments, and services provided.<br>2) Sep/Oct follow-up: assess trainings, receipt of PPE, additional responsibilities, service provision, and challenges and adaptations (asked to recall Mar-May [lockdown] and Sept)<br>Survey (mothers):<br>1) baseline: assess receipt of health and nutrition services<br>2) follow-up: how service use had changed, exposure to services, counselling messages, mode of service receipt and challenges	**BANGLADESH**<br><br>Dhaka City	**2020**<br><br>Sept-Oct	**Mothers with children <2**	**PROVISION OF SERVICE**<br>• Fewer facilities offered ANC services during lockdown when compared to before pandemic (dropping 6.6 percentage points)<br>**QUALITY OF SERVICE**<br>• Most ANC services still offered (with the exception of anthropometric measurements (20 percentage point drop for weight measurements), (29 percentage point drop for height measurements)<br>• Post-lockdown anthropometric measurements were higher post-lockdown but still below pre-pandemic levels<br>• Smaller drops reported for iron folic-acid and calcium supplementation (<4 percentage points), nutrition counselling (dropped to 12.5 percentage points post-lockdown when compared to lockdown)<br>• Other services affected but all improved post-lockdown though not to pre-pandemic levels

			**Included a baseline survey**<br>Health providers and mothers with children under 2				• Increased workloads during the pandemic was reported by 56% of providers.
Padhye (28)	2022	"…to understand the status of maternal health service provision and challenges faced by 110 pregnant and recently delivered women, 38 health care providers and 18 Village Health Sanitation and Nutrition Committee members during COVID-19 pandemic" (p. 01).	**QUALITATIVE**<br>Interviews<br>(topic guide not included)<br>Interviews were intended to:<br>understand the effect of COVID-19 and lockdown on service provision (for health care providers) and understand the role and capacity building efforts for providing access to maternal health services (VHSNC) in their village.<br>Health care providers and Village Health Sanitation and Nutrition Committee members (VHSNC)	**INDIA**<br><br>Two districts in Assam (Kamrup and Darrang)	**2020**<br><br>Oct-Dec	**Maternal health services**	**PROVISION OF SERVICE**<br>• Most ANC/PNC services were managed through home visits but travel to remote areas was difficult<br>• Non-availability of laboratory services was a significant gap in ANC provisioning<br>• Village-level health centres were closed for three months during lockdown<br>• Post-lockdown iron and folic acid tablets were unavailable at the village health centres<br>• Village Accredited Social Health Activists reported not being able to provide any of the expected health services during the pandemic<br>• Services that were not available included ultrasound check-up, laboratory services, health care providers, and certain health facilities<br>**QUALITY OF SERVICES**<br>• Home deliveries for high-risk women were supported by health care staff but without backup

							support from a medical officer or an auxiliary nurse midwife
PSBI Formative Research Study Group (45)	2022	"…to understand the pandemic's impact on barriers to and mitigation for strategies of care-seeking and managing possible serious bacterial infection (PSBI) in young infants" (p. 01).	**QUALITATIVE**<br>Observation, in-depth interviews, and focus groups (to ascertain trends in service provision and use across lockdown and post lockdown periods)<br>(conceptual framework included)<br>Conceptual framework lists factors influencing PSBI management:<br>**Changes in National and local policies**<br>• Conversion of hospitals into COVID-19 care centres<br>**COVID-19 care centres**<br>• Resource reallocation • Task shifting • Diversion of cases to other higher or lower health care facilities.<br>**Health Systems changes: public and private; facility and community**<br>• Functionality of outpatient and inpatient facilities • Effective referral system • Availability of human resources • Availability of essential equipment,	**BANGLADESH, ETHIOPIA, INDIA, NIGERIA, PAKISTAN**	**2020/1**<br><br>Dec 2020 – Mar 2021	**Newborn care**	**PROVISION OF SERVICE**<br>• Several hospitals were closed, offered restricted services, or converted to COVID-19 care centres<br>• Respondents discouraged the admission of infants except in an emergency<br>• Many of these problems reported to have existed pre-COVID-19<br>• Community clinics closed during lockdown<br>• Routine services (e.g. ANC, vaccination, and post-partum care) were disrupted<br>• Outpatient care was disrupted at many sites due to service suspension, limited hours, or non-availability of staff<br>• Home-based post-natal and referral were also reduced<br>**QUALITY OF SERVICES**<br>• Some facility staff indicated that staff shortages due to COVID-19, insufficient oxygen and medicines, and unhygienic conditions at facilities had led to sub-optimal care for young infants<br>• Some respondents reported poor care and limited patient time with staff who were unwilling to touch or examine infants

			supplies and medicines • Perceptions, fear and apprehensions among health care providers and community health workers • Availability of COVID-related supplies<br>**Caregivers and Community**<br>• Fear of accessing care • Needs for seeking treatment outside the home or allowing health personnel to visit home • Constraints in accessing treatment • Transportation availability, costs • Perceived barriers • Immediate competing demands • Economic stress, loss of employment, PSBI low priority<br>Health care staff (CHWs, staff nurses, medical officers, program managers, and private practitioners)				• Many of these problems reported to have existed pre-COVID-19<br>• Social distancing felt to have reduced service quality
Rao (48)	2021	"…to survey reported disruptions to small and sick newborn care worldwide and undertake thematic analysis of healthcare providers' experiences and proposed mitigation strategies to provide insights on	**QUALITATIVE**<br>Survey data<br>(survey not included)<br>Survey collected free-text data on:<br>Demographic characteristics, healthcare preparedness and response to COVID-19, the effect of COVID-19 on	**62 COUNTRIES**<br>mainly LMICs	**2020**<br>13 Jul – 13 Oct	**Newborn care**	**QUALITY OF SERVICES**<br>• Changes to newborn care reported including reallocation of unit space and reassignment of staff from newborn care to COVID-19 and other duties<br>• Oxygen supplies for newborns compromised<br>**PROVISION OF SERVICES**

		disruptions to coverage and quality of small and sick newborn care, and to identify possible solutions to protect vulnerable newborns during the COVID-19 and similar future pandemics" (p. 1).	health professionals and newborn care, and major disruptions and solutions.<br>Health providers (nurses, midwives, doctors and community health workers), public health professionals and policymakers				• Some respondents reported closure of wards (in this case KMC) due to its proximity to the COVID-19 area<br>• A hospital outbreak resulted in closure of 'mother lodger' and KMC wards<br>• Limited social distancing space affected the quality of neonatal care<br>• Fewer staff to carry out follow-up visits and fewer appointments per newborn<br>• Staff anxiety about infection compromised KMC care
Rezapour (36)	2022	"…[to] investigate the impact of the COVID-19 pandemic on the service utilization status in the Iranian PHC system" (p. 01).	**QUANTITATIVE**<br>Survey of patients<br>(text message included)<br>Indicator Actual Percentage of Service Delivery (ASPD) measured the actual number of people who have gone to PHC facilities and received the service.<br>SMS was sent to all patients via SMS. SMS message read: "Has the service been received?"<br>**Includes baseline survey**<br>PHC facility patients	**IRAN**	**2019-2021**<br><br>Mar 2019-Jan 2020 (before); Feb 2020-Feb 2021 (after)	**General (include midwifery services)**	**PROVISION OF SERVICES**<br>• There was a significant difference in ASPD for midwifery services when comparing pre-pandemic to the COVID-19 period
Sahoo (29)	2022	"…[to] explore the experiences of	**QUALITATIVE**	**INDIA**	**2020**	**ANC, nutritional**	**PROVISION OF SERVICES**

		vulnerable communities—urban-slum-dwelling women regarding maternal and child health services during COVID-19" (p. 01).	In-depth interviews [about experiences including coping strategies, and health system readiness concerning participants' MCH care]<br>(topic guide included)<br>Topic guide questions:<br>Q1. Please share your experience during the COVID-19 pandemic?<br>Q2. What is your experience with availing antenatal services/Intra/postnatal services/immunization services during a regular period in your community? Probe: availability—site, day, time, staff, logistics, transport, appropriateness—technical and professional adequacy, approachability, acceptability, affordability, continuity of services<br>Q3. What changes have you seen in services during the COVID-19 situation? Probe: availability, appropriateness, approachability, acceptability, affordability, continuity of services<br>Q4. How did you manage the care of your sick infants during the pandemic?	Four states: Odisha, Uttarakhand, Chhattisgarh, and Assam.	Apr-May	**care for pregnant women, intra-natal and post-natal care, immunisation, and sick infant treatment**	• The majority of respondents reported that they received the antenatal check-up whilst in lockdown<br>• Some intra-natal services were not provided by the facility<br>• Timing of immunisation services were changed to accommodate staff COVID-19 duties

			Q5. How did you cope with the situation?<br>Q6. In your view, how can we strengthen the services in an emergency?<br>Urban slum dwelling women who were antenatal, intra-natal, or postnatal				
Saso (49)	2020	"…[t]o understand immunization service interruptions specifically for maternal, neonatal and infant vaccines" (p. 01).	**BOTH**<br>Survey data<br>(survey question included)<br>Survey questions:<br>Are you aware of any issues in delivering these vaccines successfully during the COVID-19 pandemic? (Yes/No/Don't know)<br>If you answered yes to the above question, please provide some detail below (e.g. administration problems, rescheduling, staff shortage, parental concerns, social/logistical barriers, others)<br>Health care workers (e.g. doctors, midwives, nurses, lab scientists, public health officials)	**51 COUNTRIES**<br>Includes 32 (63%) LMICs	**2020**<br>15 Apr-30 Apr	**Maternal and infant vaccination**	**PROVISION OF SERVICE**<br>• 50% of respondents reported disruption in either maternal or infant vaccine delivery (similarly reported across HICs and LMICs)<br>• Disruption newborn vaccination was reported by 42% (n=15) of LMIC-based respondents<br>• Disruption in maternal vaccination was reported by 53% (n=19)<br>• Disruption to clinic services was reported by most participants<br>• Issues secondary to vaccine shortages and logistics primarily described within LMICs<br>**QUALITY OF SERVICE**<br>• Staff shortages were described more frequently in the context of newborn, infant, toddler vaccination<br>• The barriers described in LMICs were more skewed towards supply-side issues
Semaan (50)	2022	"…to explore PNC provision, availability,	**QUANTITATIVE**	**61 COUNTRIES**	**2020**	**Post-natal care (mothers**	**QUALITY OF SERVICE**

		content and quality following the early phase of the COVID-19 pandemic" (p. 01).	Survey data (questions developed iteratively from past surveys)<br>(survey questions provided)<br>Survey questions:<br>In the past month, how was inpatient postnatal care for women and newborns affected? (Please select all that apply)<br>In the past month, how was outpatient postnatal care affected? (Please select all that apply)<br>In the past month, was the content of inpatient postnatal care to women and newborns provided by you or in your facility affected in terms of: (Please select all that apply)<br>In the past month, was the content of outpatient postnatal care to women and newborns provided by you or in your facility affected in terms of: (Please select all that apply)<br>Maternal and newborn health providers	Includes 34 LMICs	Jul-Dec	**and newborns)**	• Amongst LMIC respondents 37% reported reduced numbers of beds due to social distancing<br>• Amongst LMIC respondents 14% reported reduced space in postnatal ward<br>**PROVISION OF SERVICE**<br>• Amongst LMIC respondents 53% reported that appointments were scheduled further apart<br>• Amongst LMIC respondents 28% reported not being able to provide postnatal care face-to-face for all patients<br>• Amongst LMIC respondents 20 reported that home visits had stopped<br>• Amongst LMIC respondents 8% reported suspension of outpatient postnatal care<br>• Amongst LMIC respondents 22% reported shorter operating hours or fewer days for postnatal care<br>• Amongst LMIC respondents 6% reported suspending inpatient postnatal care
Shrivastava (30)	2021	"…to understand the challenges faced by pregnant women	**MEDIA ANALYSIS**<br>Content analysis (News articles reporting on the	INDIA	2020	Pregnant women	**PROVISION OF SERVICE**

		seeking institutional care during the lockdown" (p. 202).	challenges that pregnant women faced when seeking institutional care during the lockdown)<br>Pregnant women seeking institutional care	17 states	Mar-May		• Night shift staff were reassigned to other duties to address HR gaps caused by COVID-19<br>**QUALITY OF SERVICE**<br>• Pregnant women experienced poor quality care during lockdown; related to systemic health system issues, or individual care
Sinha (31)	2022	"…[t]o estimate utilization of maternal, perinatal healthcare services after the lockdown was implemented in response to the COVID-19 pandemic compared to the period before" (p. 01).	**QUALITATIVE**<br>Semi-structured survey and in-depth interviews with a sub-sample<br>(no survey questions or topic guide provided)<br>Survey included:<br>Sociodemographic, antenatal, intrapartum and postnatal care details including out-of-pocket expenditure<br>Interview topics:<br>Experiences, challenges, and barriers in accessing health care services.<br>Women who had recently delivered	INDIA<br><br>Delhi	2019/20<br><br>Oct 2019 – Mar 2020 (pre-lockdown)<br><br>Mar 2020 – Nov 2020 (after lockdown)	Maternal and perinatal services	**PROVISION OF SERVICE**<br>• Many hospitals had been converted into CVODI-19 care centres<br>• Respondents reported that community workers were unavailable during lockdown for AND or postnatal services<br>**QUALITY OF SERVICE**<br>ANC (survey)<br>• The most common barriers to availing ANC services were poor quality of care (55%), lack of transport (41%), or long waiting times (39%).<br>ANC (interviews)<br>• Accounts of poor quality care and staff reluctant to carry out examinations<br>Intrapartum care (survey)<br>• 28% of respondents reported issues with intrapartum care<br>Intrapartum care (interviews)

							• Accounts of fear of contracting COVID-19, poor quality of care, and availability of few or no health staff<br>• Respondents found labour room experience distressing due to shortage of staff and unhygienic facilities
Tadesse (25)	2020	"…[t]o assess the impact of the COVID-19 pandemic on antenatal care utilization among pregnant women attending public facilities in Northeast Ethiopia" (p. 1181).	**QUANTITATIVE**<br>Interviewer-administered questionnaire<br>(questionnaire provided)<br>Did you miss or late to start ANC visit during COVID-19 outbreak? (Yes/No)<br>Facility related factors:<br>1) deploying of maternal care workers (Yes/No)<br>2) interruption and diversion of maternity services to COVID-19 response (Yes/No)<br>3) Fear of COVID-19 infection (Yes/No)<br>4) Social distancing (Yes/No)<br>Pregnant women	**ETHIOPIA**	**2020**<br>Feb-Aug	**ANC**	**PROVISION OF SERVICE**<br>• Interruption and diversion of maternal health services due to COVID-19 reduced full service use by 32%
Tilahun (26)	2022	"…to examine the effect of the COVID-19 pandemic on MNCH service uptake and to identify effective strategies to ensure MNCH service delivery	**QUALITATIVE**<br>In depth interviews<br>(no interview guide; no description of interview guide)	**ETHIOPIA**	**2020**	**Maternal and child health services**	**PROVISION OF SERVICE**<br>• Respondents reported low commitment of health care workers, movement restrictions, and shortage of resources were

		during the pandemic in Ethiopia" (p. 2782).	Decision-makers, health workers, NGO delegates and clients				felt to have reduced the quality of services
Ulaganeethi (32)	2021	"…to assess the proportion of pregnant women who had not completed the ideal number of antenatal visits, availability of iron-folic acid (IFA) supplements and challenges in availing health services during the period of lockdown" (p. i23).	**QUALITATIVE**<br>Semi-structured interviews<br>(Interview guide provided)<br>Interviews included:<br>3. Did you face any difficulty in availing antenatal care services or healthcare services for any danger sign you noticed during this lockdown? Can you […] your experience?<br>4. Why you were not able avail the antenatal services? (Probe: non-availability of transportation facilities, fear of visiting the hospital, anxiety to go out of house, family support, getting medicines, fear of getting COVID, spread of COVID to child, delivery, income).<br>Pregnant women	**INDIA**<br><br>Puducherry	2020<br><br>Jul-Jun	ANC	**PROVISION OF SERVICE**<br>• Respondents indicated that health facilities closed down their outpatient clinics during lockdown
Villalobos Dintrans (46)	2021	"…[t]o describe the perception of key actors regarding the interruption of health services for populations not prioritized by the pandemic —pregnant women, newborns,	**QUANTITATIVE**<br>Survey (survey questions provided)<br>Question:<br>For the following health actions for pregnant women, newborns, children,	**19 Latin American and Caribbean countries**	2020	General (includes MNH services)	**PROVISION OF SERVICE**<br>Services for newborns and pregnant women, such as institutional delivery care and postpartum care, as well as vaccination programs, showed the

		children, adolescents, and women— in Latin American and Caribbean (LAC) countries during the first stage of the COVID-19 pandemic" (p. 01).	adolescents and women, at a general level, indicate the state in your country during the month of June 2021. [have been maintained, have been partially reduced, have been suspended, Adapted to new circumstances, created new services, don't know].<br>Decision-makers and implementers of social and health programs and policies				best perceptions of coverage during the pandemic.
Widiasih (34)	2021	"…to explore the experiences of pregnant women regarding ANC and monitoring fetal wellbeing during the COVID-19 pandemic" (p. 01).	**QUALITATIVE**<br>Semi-structured interview<br>(no interview guide provided)<br>Interviews described as:<br>"…covering the pregnant woman's experience when engaged in ANC and how and when they undertook fetal welfare checks during the COVID-19 pandemic".<br>Pregnant women	INDONESIA	2021<br>Jan	Self-foetal wellbeing monitoring and ANC	**PROVISION OF SERVICE**<br>• Respondents reported queues to access care, limits on the number of patients, and changes to service hours
