# Supplementary material for: Measuring supply-side service disruption: a systematic review of the methods for measuring disruption in the context of maternal and newborn health services in low and middle-income settings
Source: BMJ Open. 2023 Dec 9;13(12):e077583. doi: 10.1136/bmjopen-2023-077583 (PMC10729240; doi:10.1136/bmjopen-2023-077583)
Supplement: Author's manuscript [file bmjopen-2023-077583.draft_revisions.pdf]

BMJ Open is committed to open peer review. As part of this commitment we make the peer review history of every article we publish publicly available.

When an article is published we post the peer reviewers' comments and the authors' responses online. We also post the versions of the paper that were used during peer review. These are the versions that the peer review comments apply to.

The versions of the paper that follow are the versions that were submitted during the peer review process. They are not the versions of record or the final published versions. They should not be cited or distributed as the published version of this manuscript.

BMJ Open is an open access journal and the full, final, typeset and author-corrected version of record of the manuscript is available on our site with no access controls, subscription charges or pay-per-view fees (http://bmjopen.bmj.com).

If you have any questions on BMJ Open's open peer review process please email info.bmjopen@bmj.com

BMJ Open

Measuring supply-side service disruption: A systematic review of the methods for measuring disruption in the context of maternal and newborn health services in low- and middle-income settings

Journal:	*BMJ Open*
Manuscript ID	bmjopen-2023-077583
Article Type:	Original research
Date Submitted by the Author:	09-Jul-2023
Complete List of Authors:	McGowan, Catherine; London School of Hygiene & Tropical Medicine, Department of Infectious Disease Epidemiology<br>Gokulakrishnan, Dhikshitha; Leeds Teaching Hospitals NHS Trust<br>Monaghan, Emily; Save the Children International, Humanitarian Department; Croydon University Hospital<br>Abdelmagid, Nada ; London School of Hygiene & Tropical Medicine, Department of Infectious Disease Epidemiology<br>Romig, Laura; Save the Children Federation Inc, Department of Humanitarian Response<br>Gallagher, Meghan; Save the Children Federation Inc, Department of Humanitarian Response<br>Meyers, Janet; Save the Children Federation Inc, Department of Humanitarian Response<br>Cummings, Rachael; London School of Hygiene & Tropical Medicine, PHP; Save the Children International, Humanitarian Department<br>Cardinal, Laura; Save the Children International, Humanitarian Department
Keywords:	COVID-19, Systematic Review, Quality in health care < HEALTH SERVICES ADMINISTRATION & MANAGEMENT, Maternal medicine < OBSTETRICS

SCHOLARONE™
Manuscripts

I, the Submitting Author has the right to grant and does grant on behalf of all authors of the Work (as defined in the below author licence), an exclusive licence and/or a non-exclusive licence for contributions from authors who are: i) UK Crown employees; ii) where BMJ has agreed a CC-BY licence shall apply, and/or iii) in accordance with the terms applicable for US Federal Government officers or employees acting as part of their official duties; on a worldwide, perpetual, irrevocable, royalty-free basis to BMJ Publishing Group Ltd ("BMJ") its licensees and where the relevant Journal is co-owned by BMJ to the co-owners of the Journal, to publish the Work in this journal and any other BMJ products and to exploit all rights, as set out in our licence.

The Submitting Author accepts and understands that any supply made under these terms is made by BMJ to the Submitting Author unless you are acting as an employee on behalf of your employer or a postgraduate student of an affiliated institution which is paying any applicable article publishing charge ("APC") for Open Access articles. Where the Submitting Author wishes to make the Work available on an Open Access basis (and intends to pay the relevant APC), the terms of reuse of such Open Access shall be governed by a Creative Commons licence – details of these licences and which Creative Commons licence will apply to this Work are set out in our licence referred to above.

Other than as permitted in any relevant BMJ Author's Self Archiving Policies, I confirm this Work has not been accepted for publication elsewhere, is not being considered for publication elsewhere and does not duplicate material already published. I confirm all authors consent to publication of this Work and authorise the granting of this licence.

For peer review only

Measuring supply-side service disruption: A systematic review of the methods for measuring disruption in the context of maternal and newborn health services in low- and middle-income settings

For peer review only

Catherine R. McGowan[1†], Dhikshitha Gokulakrishnan[2], Emily Monaghan[3,4], Nada Abdelmagid[1], Laura Romig[3], Meghan Gallagher[5], Janet Meyers[3], Rachael Cummings[3,6], Laura Cardinal[3]

1. Faculty of Epidemiology and Population Health, London School of Hygiene & Tropical Medicine, Keppel Street, London WC1E 7HT UK
2. Leeds Teaching Hospitals NHS Trust, Great George St, Leeds LS1 3EX
3. Humanitarian Department, Save the Children International, St Vincent House, 30 Orange Street, London, WC2H 7HH
4. Croyden University Hospital, 530 London Road, London CR7 7YE
5. Department of Global Health, Save the Children Federation Inc, 899 N. Capitol St #900, NE, Washington, DC 20002
6. Faculty of Public Health and Policy, London School of Hygiene & Tropical Medicine, 15-17Tavistock Place, London WC1H 9SH

WORDS: 4968

† Corresponding author:

Catherine R. McGowan
Department of Infectious Disease Epidemiology
London School of Hygiene & Tropical Medicine
Keppel Street
London WC1E 7HT
United Kingdom
Catherine.McGowan@lshtm.ac.uk

ABSTRACT

Background: During the COVID-19 pandemic most essential services experienced some level of disruption. Disruption in LMICs was more severe than in HICs. Early evidence of disruption suggested that services for maternal and newborn health were disproportionately affected raising concerns about health equity. Most indicators of disruption measure either demand-side disruption only, or they conflate demand- and supply-side disruption. There is currently no published guidance on measuring supply-side disruption. The objective of this systematic review was to identify the methods and approaches that have been used to measure supply-side service disruptions to maternal and newborn health services in the context of COVID-19.

Methods: We searched MEDLINE, EMBASE, and Global Health in January 2023 for empirical studies describing methods and approaches to measuring supply-side service disruption of maternal and newborn health services in LMICs. We also searched the grey literature. We included measures which isolate supply-side disruption to the provision or quality of services. We present a typology of measurement methods and approaches using narrative synthesis.

Results: We identified 28 studies. 19 studies used qualitative methods to measure disruption, seven used quantitative methods, and two used mixed-methods. Studies involved service providers (n=14), service users (n=11), or both (n=3). Five approaches were used: 1) cross-sectional surveys of the nature and experience of supply-side disruption, 2) surveys to measure temporal changes in service provision or quality, 3) surveys to create composite disruption scores, 4) surveys of service users to measure receipt of services, and 5) clinic observation of the provision and quality of services.

Discussion: Our review identified methods and approaches for measuring supply-side service disruption of maternal and newborn health services. These indicators provide important information about the causes and extent of supply-side disruption and provide a useful starting point for developing specific guidance describing meaningful metrics for measuring service disruption in LMICs.

WORDS: 300

For peer review only

STREGHTS AND LIMITATIONS

- Despite the absence of guidance, meaningful attempts have been made to measures supply-side service disruption of maternal and newborn health services in the context of COVID-19
- Many of the included sources aimed to measure supply-side disruption yet provided crude measures of service provision that likely conflate supply-side and demand-side disruption
- Measuring disruption may be viewed as a liability, or may be considered demoralising to service-providers, and may have resulted in a reluctance to measure disruption or publish findings
- Publication delay may explain the small number od studies identified

For peer review only

INTRODUCTION

With few exceptions, the COVID-19 pandemic exposed the vulnerabilities of health systems globally. In response to the burden of COVID-19 cases, many countries closed or scaled-back non-essential health services to allow relocation of resources to the COVID-19 response, and to reduce health system strain. In mid-2020 the WHO conducted the first of three key informant surveys amongst national health authorities in 105 countries (1). These 'pulse surveys' aimed to assess the impact of COVID-19 on 25 essential health services. Nearly all countries reported service disruptions, with lower-income countries reporting greater service disruption than higher income countries. The causes of disruption were identified as a mixture of service demand and supply factors. The most reported supply-side cause of disruption was the cancellation of elective services (reported by 66% of countries). Other supply-side causes included: staff redeployment to COVID-19 response (49%) or insufficient staff to provide services (29%), insufficient personal protective equipment for health care workers (44%), closure of health services (33%) or facilities (41%), and interruptions in the availability of medical supplies (30%) (1). Demand-side indicators typically equate to indicators of service uptake which may evidence a reluctance to present to health services, or challenges with access. Demand-side indicators may demonstrate reduced uptake despite continuity, or scale up, of services.

Defining a package of essential services is a key preparedness activity; however, scaling back non-essential services may not be sufficient to avoid widespread and lasting disruption. The latest WHO pulse survey (published May 2023) notes that despite evidence of health system recovery, 84% of participating countries reported ongoing disruption to essential health services in the last quarter of 2022 (2), suggesting that disruption persists well into the recovery period. In June 2020 the WHO published operational guidance for maintaining essential health services during the pandemic (3). The guidance cautions that all health system adaptations "*…should be made in accordance with ethical principles, such as equity in the allocation of resources and access, self-determination, non-abandonment, and respect for dignity and human rights*" (3, p. 03). Though every country determines its own essential health services package based on its local contexts and capacities, the WHO recommended that seven health service categories be maintained during the acute phase of the pandemic including: 1) prevention and treatment of communicable diseases, 2) reproductive health services (including obstetric and child health), 3) core services for vulnerable populations (including infants and older adults), 4) chronic diseases (including mental health conditions), 5) critical facility-based therapies, 6) management of emergency health conditions, and 7) auxiliary services (including diagnostic imaging and laboratory services). The guidance emphasised that

For peer review only - http://bmjopen.bmj.com/site/about/guidelines.xhtml

critical decisions about the nature and timing of modifications to service delivery, "*… must be informed by the use of accurate and timely data throughout all phases of the COVID-19 pandemic*" (3, p. 18).

Data on service disruption should be suitable to inform rapid and appropriate adjustments (e.g. workforce optimisation strategies, resource allocation adaptations), including adjustments across essential services to ensure health equity (4). Whilst supply-side disruptions may contribute to decreased uptake (and vice versa), defining indicators which isolate the direction of disruption can inform measurements of the extent of demand-side disruption that can be attributed to supply-side disruption. In addition, monitoring essential health services requires meaningfully disaggregated data (to ensure that services are being delivered equitably), data that allow comparison of disruption over time (e.g. across key phases of the pandemic), and rapid needs assessments to inform facility-level priorities (3, 5). The WHO guidance on maintaining essential health services includes an extended list of sample indicators for measuring disruption; however, most of these indicators measure demand-side disruption only (e.g. number of women presenting to facility) or conflate demand-side and supply-side disruption (e.g. number of facility births).

Disruption of maternal and newborn health services during COVID-19

The negative impact of the COVID-19 pandemic on maternal and newborn health (MNH) outcomes has been well established at the population level. However, the causal pathways that create poor outcomes are less understood (6). The evidence suggesting a direct link between maternal COVID-19 infection and maternal/newborn outcomes is limited (7, 8). It is suspected that disruption to MNH services – either in terms of reduced overall provision of services, or reduced quality of services – was primarily driving poor MNH outcomes (9). It has been estimated that supply and demand side disruption, combined with increasing food insecurity, could lead to between 12, 200 and 56, 700 excess maternal deaths globally (10). Vulnerable populations, including women and children, are experiencing the greatest effects (both direct and indirect) of the pandemic, thereby widening existing inequities (11) and reversing progress towards improving the health of these and other vulnerable groups (6, 9).

Though nearly all countries reported disruptions to essential health services, disruptions were often more severe in low- and middle-income (LMIC) countries (12). Whilst all essential health services have been disrupted by COVID-19, some have been disproportionately affected (12). Evidence suggests that MNH services have been disproportionately impacted by COVID-19-related service disruption, particularly in LMIC settings (13, 14). A scoping review of the impact of COVID-19 on maternal, newborn, child health and nutrition concluded that a considerable amount of funding for

For peer review only - http://bmjopen.bmj.com/site/about/guidelines.xhtml

these services in fragile and conflict-affected settings had been diverted to COVID-19 response activities (15). A recent systematic review aiming to synthesise the direct and indirect effects of the pandemic concluded that, "*…minor consideration was given to preserving and promoting health service access and utilization for mothers and children, especially in historically underserved areas in Africa*" (16, p. 1).

Rationale

The need for better service coverage data was identified early in the pandemic as the limitations of routinely collected data for reporting disruption became apparent (4). The literature currently overrepresents demand-side disruptions. Many crude health outcome indicators, such as number of stillbirths and maternal mortality, conflate supply-side and demand-side disruptions. Relatively few reports of COVID-19-related service disruption aim to isolate supply-side disruption yet measuring supply side disruptions is key to mitigating their effects. Moreover, we were unable to identify comprehensive guidance for measuring supply-side disruption of MNH services. This review aims to address this gap by describing how COVID-19-related, supply-side disruption has been measured in the context of MNH services in LMICs.

Objectives

The objectives of this review are: 1) to present methods and approaches used to *quantitatively* measure supply-side service disruptions in LMICs in the context of COVID-19 and MNH services, and 2) to consolidate the *qualitative* evidence of supply-side service and quality disruptions to MNH services in LMICs.

This review is reported against the PRISMA 2020 checklist (17) and updated reporting guidance (18). The review protocol has been published in the PROSPERO prospective systematic review registry (CRD42022381537) (19).

METHODS

Eligibility criteria

Eligibility was defined as any empirical source that reported on at least one indicator of supply-side disruption, regardless of method (i.e. quantitative or qualitative). We defined supply-side disruption broadly to include cancelling/scaling back of a MNH service, or reduction in quality (including reduction in staff numbers). We included studies reporting on any specific MNH clinical service (e.g. childbirth, antenatal care [ANC], postnatal care [PNC] for mothers and newborns, essential newborn care), MNH services more broadly (even if their components were not described), or studies of

For peer review only - http://bmjopen.bmj.com/site/about/guidelines.xhtml

multiple essential services disaggregated by service and including at least one MNH service. We define supply-side disruption broadly to include disruption to the *provision* of care (e.g. services closed or reduced hours), and disruption that compromises *quality* of care (e.g. decreased consultation time with providers, decreased health staff on duty, or fewer services and/or procedures offered). We did not include in either category perceived disruptions to quality of care attributed to minimal and reasonable infection prevention and control requirements (e.g. the requirement to wear masks during consultation, social distancing whilst in a queue).

We did *not* include reports of disruption solely in the context of family planning or sexual health services; studies reporting only on MNH service uptake or indicators which combine or conflate demand side and supply side disruptions; or comparisons of baseline (e.g. pre-COVID) and follow-up outcome indicators (e.g. stillbirths) without isolating the effect of supply-side disruption. Sources were also excluded if they reported on supply chain disruption (e.g. blood for transfusion) without reporting on resulting discontinuation, scale-down, or reduced quality of services. We included sources written in any language. Finally, we excluded pre-prints.

Information sources and search strategy

We searched MEDLINE, EMBASE, and Global Health databases (via Ovid) for peer-reviewed and relevant grey literature on 23 January 2023; no start date was used as this was obviated by the inclusion of COVID-19 as a search term. We adapted our search strategy from the search methods used to maintain and update the Cochrane Pregnancy and Childbirth Group's Specialised Register (20). The complete database search strategy is included in **Appendix 1**. The grey literature searches were carried out using Google Search (26 February 2023) and Duck Duck Go (23 February 2023) using independent search strings including: 'service disruption' and COVID; 'essential service disruption'; maternal and newborn and 'service disruption'; maternal and newborn and services and COVID. The team also searched relevant websites including the WHO Global Pulse Survey website (21) and the WHO COVID-19 Technical Guidance for Maintaining Essential Services and Systems website (22).

Selection process, data collection and synthesis

The lead author [CM] carried out the initial screening of all sources retrieved via database searching. Screening of grey literature sources was carried out by two authors [EM/LR]. The lead author manually extracted all data in NVivo 1.0 (Melbourne, AU: QSR International Pty Ltd) based on the following characteristics and domains: title, year, author, aim, method, setting, time (data collection), programme or population, findings, and definition of disruption. We compiled a narrative synthesis of the literature. Only data on supply-side disruption were extracted from

For peer review only - http://bmjopen.bmj.com/site/about/guidelines.xhtml

sources reporting on both demand-side and supply-side factors. We define *quality of service* in line with WHO definition of quality (i.e. the service is effective, safe, people-centred, timely, equitable, integrated, and efficient) (23). We define *provision of service* to include cessation or scale back of services.

Reporting bias and certainty assessment

We did not assess bias of the studies themselves. Whilst we had intended to use an established guideline to evaluate the quantitative indicators identified in the literature, no source provided sufficient information to allow a complete quality assessment.

Patient and public involvement

All authors have worked professionally as humanitarians (with a specialisation in MNH and/or sexual and reproductive health), and/or in clinical medicine/allied health services. Specific members of the review team have direct experience in the design, delivery, and evaluation of MNH services in LMICs [EM/NA/MG/JM/RC].

RESULTS

Study selection

The initial database search returned 4,438 records published on or before 23 January 2023. We used EndNote 20.5 (Philadelphia, PA: Clarivate) to delete duplicates which resulted in 2657 unique records. Initial screening was carried out based on review of the title/abstract and resulted in 94 sources for which we were able to retrieve 85 full-text documents. 28 peer-reviewed sources and three grey literature sources (reported below as one source) remained following full-text review. The PRISMA Flow chart is included in **Figure 1**.

INSERT FIGURE 1 HERE

The reasons for exclusion, following full-text review, were: only reported on uptake (n=18), not sufficiently focussed on disruption (n=18), not sufficiently focussed on MNH (n=5), not sufficiently focussed on LMICs (n=2), review article (n=3), not empirical (n=2), no disruption (n=3), pre-print (n=6), and evaluation of a particular MNH intervention (n=1). The grey literature search produced 43 unique results of which 10 were included in the final synthesis. None of the grey literature sources met the inclusion criteria as: they were not empirical (n=9), were not focussed on MNH (n=2), presented only demand-side indicators (n=6), or they included indicators of disruption which did not isolate supply-side factors (n=4).

For peer review only - http://bmjopen.bmj.com/site/about/guidelines.xhtml

Study characteristics

The characteristics of the 28 included studies are listed in **Appendix 2**. Studies were carried out in: Bangladesh (24), Ethiopia (25, 26), India (27, 28, 29, 30, 31, 32), Indonesia (33, 34), Iran (35, 36), Nepal (37, 38), Nigeria (39, 40, 41, 42), and Uganda (43, 44). Three studies included multiple LMIC countries (13, 45, 46), and four included both HIC and LMIC countries (47, 48, 49, 50). Most studies (n=18) used qualitative methods to collect data about supply-side disruption (26, 28, 29, 31, 32, 33, 34, 35, 38, 39, 40, 41, 42, 43, 44, 45, 47, 48); one study involved a qualitative media analysis (30). Seven studies were based on quantitative methods (13, 24, 25, 27, 36, 46, 50), and two used both qualitative and quantitative methods (37, 49). Most studies (n= 14) involved participation from service-providers (e.g. nurses, midwives, obstetricians, community health workers, policy-makers) (13, 27, 28, 33, 38, 40, 41, 42, 45, 46, 47, 48, 49, 50), whilst 11 involved service-users (25, 29, 30, 31, 32, 34, 35, 36, 37, 43, 44), and three included both groups (24, 26, 39).

Results of synthesis

We report below a synthesis of the quantitative approaches used to measure supply-side disruptions to the provision of services and/or the quality of services due to COVID-19 – a summary of these findings can be found in **Tables 1 and 2**. In addition, we summarise the qualitative approaches to measuring supply-side disruption.

INSERT TABLE 1 HERE

Quantitative measures of disruption

Quantitative indicator data were collected using the following approaches:

Surveys of health care workers or health stakeholders about disruptions (49, 50) or missed services (25) were carried out in four studies. The first involved distributing a survey amongst health care workers in 51 countries (including 32 LMICs) – asking if they were aware of any problems providing MNH immunisation services (yes/no) – if respondents answered yes, they were asked to indicate the nature of the problems (described below in the section on qualitative measures) (49). This approach produced a crude proportionate measure of subjective assessment of disruption to vaccine delivery across several countries (e.g. 53% of LMIC-based respondents reported disruptions to maternal immunisation services compared to 42% of HIC-based respondents).

Using a pre-defined list of disruptions to the provision and quality of services, a study in 61 countries (including 34 LMICs) asked health care workers to indicate how specific MNH services (e.g. inpatient/outpatient post-natal care) were affected by COVID-19 in the preceding month (50). This study produced proportionate measures of the subjective assessment of the causes of disruption to post-natal care across multiple countries; for example, respondents from LMICs reported various

For peer review only - http://bmjopen.bmj.com/site/about/guidelines.xhtml

disruptions due to COVID-19 including reduced numbers of beds due to social distancing [37%], cessation of home visits [20%], and suspension of post-natal care [8%] (50).

A study amongst health stakeholders in 19 Latin American and Caribbean countries asked respondents to indicate their perception of the disruption to various health services (including MNH) during the pandemic using a Likert scale (i.e. services have been maintained, partially reduced, suspended, adapted to COVID-19, and new services were created) (46). The study concluded that services for newborns and pregnant women (e.g. institutional delivery care, postpartum care, vaccination programs) were found to have showed the best perceptions of coverage during the pandemic (46).

Finally, a survey to assess disruption to ANC services was sent to pregnant women in Ethiopia asking, '*Did you miss or were late to start an ANC visit during the COVID-19 outbreak?'* (yes/no) (25). Respondents were asked to indicate a reason for missed or delayed ANC visits from a selection of responses describing facility-related factors for disruption; such as, "deploying of maternal care workers", "interruption and diversion of maternity services to the COVID-19 response", "fear of COVID-19 infection", and "social distancing". The study produced a measure of the proportion of respondents who experienced a disrupted ANC visit (which does not isolate supply-side factors) as well as a proportionate subjective assessment of the causes of the disruption. Two-hundred and sixteen (out of 389) respondents reported missed or late ANC visits, of which 72 [33.33%] attributed this to diversion of maternal services to COVID-19 (25).

INSERT TABLE 2 HERE

Surveys of health care workers or health stakeholders (to measure temporal changes in disruption) were used in two ways: 1) in India retrospective baseline data, in addition to accounts of the situation at the time of the interview were compared (27), and 2) in Bangladesh, data about service provision and service quality were collected from health care workers and mothers with children under two at baseline (i.e. time 1 [T1]), and at T3 (which includes retrospective questions about T2) (24).

The study from India involved sending a survey to health care workers asking them if they were able to provide ANC services to all pregnant women during the most recent lockdown (T1), and in the preceding month (T2). Responses were: 0 (none), 1 (yes, but only to some pregnant women), or 2 (yes, to all pregnant women). Data about pre-pandemic service provision (T0) were collected from hospital HIS systems (27); however, though these data were chosen to provide a baseline measure that aligned with the questions in the survey, the health information system (HIS) indicators (i.e. # of women receiving >4 ANC check-ups, and the number of women given iron and folic acid tablets)

For peer review only - http://bmjopen.bmj.com/site/about/guidelines.xhtml

conflate demand-side and supply-side factors. The study provided the proportion of women provided ANC services at T1 and T2 (as a measure of changes in service provision), and the number of women receiving services at T0. The study found that ANC services were disrupted during the lockdown period but were restored during the post-lockdown period, but not to pre-pandemic levels) (27).

The study from Bangladesh included a baseline assessment carried out in early 2020 asking health care workers about their exposure to training, their workload and time commitments, and the types of services, specifically health and nutrition services for mothers and young children, they were providing (e.g. was service X provided in T1) (24). Data were also collected from pregnant women and mothers of young children (e.g. was service X received) at the same time points. The study provides a proportionate measure of service provision (defined as the proportion of service-providers indicating provision of specific services, and the proportion of service-users indicating receipt of specific services) at pre-pandemic (T0), lockdown (T1), and post lockdown (T2). The study found that fewer facilities offered ANC services during lockdown when compared to before the pandemic (dropping 6.6 percentage points), and that there were significant drops in the receipt of anthropometric measurements, and iron/folic acid/calcium supplementation. All services recovered post-lockdown but not to pre-pandemic levels (24).

Surveys of health care workers to construct composite service disruption scores for specific services (including MNH services) were used in Burkina Faso, Ethiopia, and Nigeria (13). Responses were scored 0 (no interruption), 1 (partial interruption), 2 (complete interruption) for each of the three MNH services (i.e. ANC, iron and folate supplementation, and family planning services) for a maximum disruption score of six. The average of all individual responses was calculated for each of the three study settings. Maternal and reproductive services were scored an average of 2.24 (out of six) for Nigeria, 1.72 for Burkina Faso, and 1.67 for Ethiopia (13).

Survey of service users to measure receipt of health services was carried out in Iran to assess the Actual Percentage of Service Delivery (APSD) (36). An SMS message - i.e. *'Has the service been received?'* (yes/no) - was sent to all patients attending hospital services. Data were collected before and during the pandemic and were aggregated by calendar month. The study indicates the proportion of attendees who received a service, by month (e.g. the study found that there was a small but significant drop in the ASPD for midwifery services when comparing the pandemic to pre-pandemic periods) (36).

Observation of health facilities was carried out in Nepal, using a structured clinical observation checklist, to determine the number of health care workers in the labour and delivery room, the

For peer review only - http://bmjopen.bmj.com/site/about/guidelines.xhtml

number of workers redeployed to COVID-19, and the number of days of disruption of maternity services (i.e. no maternity services provided on these days) (37). The study collected baseline data over six months in 2019 (pre-pandemic period), followed by data collection over six months in 2020 (pandemic period). The study measured the mean difference in the number of health workers in the labour room and the number of days that maternity services were disrupted between the two time periods. The study provides a mean number of days (4.3) that maternity services were halted during the pandemic period (March – August 2019), and concluded that the number of health care workers (per 24 hours in the labour and delivery room) had decreased to 5.4 (from 6.2 before the pandemic) due to redeployment to COVID-19-dedicated care (37).

Qualitative measures of disruption

Various qualitative approaches were used to generate accounts of disruptions to the provision or quality of services including: 1) semi-structured interviews with service providers and/or service users to elicit descriptions of experiences of disruption (26, 28, 29, 31, 32, 34, 35, 37, 38, 39, 40, 41, 42, 43, 44), 2) semi-structured interviews with service providers eliciting comparisons of the provision of care at different times (33, 47), 3) semi-structured interviews amongst service users asking about management, barriers, and facilitators influencing service delivery (stratified by levels of health system) (45), 4) free-text field in a survey of service providers (48, 49) and 5) media analysis of news reports (30). Some of these sources focussed on the experiences of respondents during a lockdown period (32), or during a lockdown period compared to the period preceding or following lockdown (31, 40). Only six of these studies published the original data collection instrument (29, 32, 39, 41, 45, 49).

Provision of services

Disruptions to provision of services included: closed health facilities or village health posts (28, 37), facilities that had closed certain wards/units/clinics/services (e.g. outpatient clinics (32, 42, 48), doctor's offices (35), support for kangaroo mother care (38), counselling for care of mother and newborn (38)), limits to the number of patients that could be seen (34, 44), lack of follow-up (44, 48) or home visits (33, 41), redeployment of staff to support COVID-19 services (30, 37, 41, 48), reduced beds or space (41), reduced hours of service (44, 45), health facilities converted to COVID-19 care centres (31, 45), unavailable health care workers (31, 45), lack of laboratory services (28), and specific services (e.g. intra-natal services) unavailable (29) or reduced (29, 33, 40, 41, 45).

Quality of services

Disruptions to the quality of services included: poor quality of care (30, 31, 35, 38, 44, 45, 47), long waiting times (31, 34, 43), poor attitude of health care workers towards clients (26), reduced

For peer review only - http://bmjopen.bmj.com/site/about/guidelines.xhtml

numbers of health care workers (37, 38, 42, 45), reduced physical and/or emotional support (47), staff preoccupied with infection prevention and control (44), unhygienic conditions (45), shorter times with health care workers (35, 38, 45), health care workers unwilling to perform certain examinations/procedures (e.g. abdominal palpation, auscultation of foetal heart rate) (35, 45), increased risk of medically unjustified caesarean section (47), and shortages of resources (including medicines, vaccines and/or oxygen) were also reported (26, 35, 45, 49).

DISCUSSION

Our initial screening (on title and abstract) revealed that the majority of the literature on service disruption reports on demand-side measures. This was also a finding of a recent review of health system performance indicators (51). Our search identified a small number of measures isolating supply-side factors (N=28). Amongst these, seven sources included quantitative measures, of which two focussed on the causes (as opposed to the presence or extent) of disruption (25, 50). The 19 qualitative (and two mixed methods) studies included rich descriptions of the experiences of supply-side delay from the perspective of both service-providers and service-users, yet none of the findings from these studies appear to have informed the design of quantitative surveys. Only one study used empirical findings (from a previous iteration of the study survey) to pre-define survey fields describing the nature of service disruption (50). However, we believe these qualitative studies may provide important contextual relevance and granularity to future surveys.

Emergency preparedness guidance documents often emphasise the importance of monitoring and evaluation but typically do not include indicators of health system disruption. The WHO's COVID-19 Strategic Preparedness and Response Plan states, "*…it is important to track health system capacity and performance; including hospital and ICU bed occupancy rates, as well as the health system's ability to continue providing non-COVID-19 services*"; however, a bank of specific indicators is not included (52). The evidence we identified suggests that approaches to measuring supply-side disruption are varied, inconsistent, and largely *ad hoc*. We identified a small number of measures of supply-side disruption that included real time baseline measurements; this suggests that systems ought to be in place at the onset of a pandemic to allow for the collection of baseline measurements. Retrospective baseline measurements may lack precision as they are typically prone to recall and confirmation biases. In addition, early planning for periodic follow-up is important as, "…metrics of system responses and resilience may only be understood appropriately as we relate them to the severity and dynamics of the shock itself" (53, p. 1202).

For peer review only - http://bmjopen.bmj.com/site/about/guidelines.xhtml

Facility assessments (and community assessments) designed to establish the continuity of essential services in the context of COVID-19 are important tools for monitoring the provision and quality of care; however, they may require adaptation for future outbreaks (and other crises), and may not be suitable for specific health programmes or priorities (5, 54). In addition, our review suggests that there are additional approaches to measuring supply-side disruption that may provide an important complement to facility assessments. The evidence also suggests that qualitative interviews may be a more suitable approach to collecting data about quality of services and may provide the opportunity for participants to offer localised solutions. Qualitative methods may also clarify the mechanisms through which services are affected (e.g. including cascading disruptions, proxy measures of disruption) which may help to identify targeted mitigation strategies (51).

Finally, we excluded one source reporting on a clinical review of patient records to assess the extent of unnecessary hospitalisations of pregnant women in Tajikistan (55). This study was excluded as it was focussed on demand side indicators (i.e. total hospitalisations before and during COVID-19) and only assessed unnecessary hospitalisations for two months during COVID-19 as a measure of the strength of the existing primary health care system. However, though the indicator was not suitable for assessing COVID-19 related disruption in this context, we believe that unnecessary hospital admissions (and unnecessarily prolonged admissions) could be a suitable indicator of disruption to quality of primary health care - insofar as it reflects poor organisation, poor use of resources, and reduces inpatient capacity - if collected prospectively or retrospectively to include the pre-outbreak period.

Limitations of the evidence

Many of the included sources provide weak description of methods (only six of the 28 sources include the original survey or the topic guide). Some sources aim to measure supply-side disruption but present crude measures of provision (e.g. number of ANC visits provided per day) which likely conflate supply and demand-side disruptions. Whilst the qualitative studies provide important context-specific information, they may not be transferable to other settings. Many respondents indicated that services were closed or that they were of low quality during the pandemic (or specifically during lockdown) but few sources aim to determine to what extent services were available, and at what comparative quality, prior to the pandemic. Concerns are often raised about the pandemic undermining the steady gains made in the provision of MNH services in recent years (9); thus, comparing disruption to a baseline measure fails to account for gains that would have been made otherwise and may therefore underestimate the true extent of the disruption.

For peer review only

Limitations of the review

Whilst COVID-19 related disruption was globally pervasive, there are relatively few sources focussed on the disruption of MNH services. As service disruption may have been assumed to be unavoidable (and thus not worthy of measuring), it may be underreported. Similarly, acknowledging disruption may be viewed as a liability, or may be considered demoralising to service-providers, which may have resulted in a reluctance to measure disruption or publish findings. We opted for a broad search strategy to avoid missing sources that did not specifically reference 'disruption'; however, we still may have missed relevant sources which did not reference 'service' or 'programme' in the title or abstract. In addition, we suspect that publication delay may have limited the amount of available evidence.

CONCLUSION

The evidence suggests: an overrepresentation of demand-side indicators, varied and inconsistent methods for isolating supply-side disruption, few published studies of supply-side disruption that include baseline data (with most relying on retrospective baseline estimates or subjective estimation of change over time), relatively few of the quantitative indicators are designed to measure the causes of disruption, studies reporting on supply-side disruption from the perspective of service-users, and a lack of a clear or nuanced (i.e. including both the provision of services, and quality of services) framework for defining supply-side disruption. There are few studies which measure disruption across all essential services. Whilst in some settings MNH services were reported to be less disrupted than other essential services, it is still important to measure disruption across all essential services to ensure equitable scale-down in response to supply-side constraints.

Taken together, the evidence emphasises the importance of monitoring essential health services and highlights the need for specific guidance for defining service disruption and for developing indicators that are able to isolate supply-side disruption both of the provision and quality of services. Given the difficulty of setting up robust monitoring systems during an emergency, and the value both of baseline measures and regular follow-up to identify the early signs of disruption, we recommend that strategies for measuring disruption be developed and incorporated into preparedness plans. Finally, we suggest that preparedness plans provide specific indicators – including indicators for measuring disruption to MNH services – alongside guidance on how data should be collected, and the resources required to support data collection and reporting in an emergency.

For peer review only - http://bmjopen.bmj.com/site/about/guidelines.xhtml

OTHER

This review was made possible by the generous support of the American people through the United States Agency for International Development (USAID). The contents are the responsibility of READY Initiative and do not necessarily reflect the views of USAID or the United States Government.

The review protocol has been published in the PROSPERO prospective systematic review registry (CRD42022381537).

For peer review only

For peer review only - http://bmjopen.bmj.com/site/about/guidelines.xhtml

REFERENCES

1. World Health Organization. Pulse survey on continuity of essential health services during the COVID-19 pandemic [Interim report]. Geneva: WHO; 2020.

2. World Health Organization. Fourth round of the global pulse survey on continuity of essential health services during the COVID-19 pandemic: November 2022–January 2023: interim report, 1 May 2023. Geneva: WHO; 2023.

3. World Health Organization. Maintaining essential health services: operational guidance for the COVID-19 context: interim guidance. Geneva: WHO; 2020.

4. World Health Organization. Maintaining the provision and use of services for maternal, newborn, child and adolescent health and older people during the COVID-19 pandemic: Lessons learned from 19 countries. Geneva: WHO; 2021.

5. World Health Organization. Continuity of essential health services: Facility assessment tool. Geneva: WHO; 2021.

6. Chmielewska B, Barratt I, Townsend R, Kalafat E, van der Meulen J, Gurol-Urganci I, et al. Effects of the COVID-19 pandemic on maternal and perinatal outcomes: a systematic review and meta-analysis. Lancet Glob Health. 2021;9(6):e759-e72.

7. Di Toro F, Gjoka M, Di Lorenzo G, De Santo D, De Seta F, Maso G, et al. Impact of COVID-19 on maternal and neonatal outcomes: a systematic review and meta-analysis. Clin Microbiol Infect. 2021;27(1):36-46.

8. Kotlar B, Gerson E, Petrillo S, Langer A, Tiemeier H. The impact of the COVID-19 pandemic on maternal and perinatal health: a scoping review. Reprod Health. 2021;18(1):10.

9. Srivastava K. Covid-19: Why has India had a spike in stillbirths? BMJ. 2021;374:n2133.

10. Roberton T, Carter ED, Chou VB, Stegmuller AR, Jackson BD, Tam Y, et al. Early estimates of the indirect effects of the COVID-19 pandemic on maternal and child mortality in low-income and middle-income countries: a modelling study. Lancet Glob Health. 2020;8(7):e901-e8.

11. Gurley N, Ebeling E, Bennett A, Kashondo JK, Ogawa VA, Couteau C, et al. National policy responses to maintain essential health services during the COVID-19 pandemic. Bull World Health Organ. 2022;100(2):168-70.

12. World Health Organization. Pulse survey on continuity of essential health services during the COVID-19 pandemic: interim report, 27 August 2020. Geneva: WHO; 2020.

13. Assefa N, Sie A, Wang D, Korte ML, Hemler EC, Abdullahi YY, et al. Reported Barriers to Healthcare Access and Service Disruptions Caused by COVID-19 in Burkina Faso, Ethiopia, and Nigeria: A Telephone Survey. Am J Trop Med Hyg. 2021;105(2):323-30.

For peer review only - http://bmjopen.bmj.com/site/about/guidelines.xhtml

14. United Nations Children's Fund. Futures at risk: The impact of COVID-19 on maternal, newborn, and child health in fragile and conflict-affected settings. New York, NY: UNICEF; 2021.

15. Rodo M, Singh L, Russell N, Singh NS. A mixed methods study to assess the impact of COVID-19 on maternal, newborn, child health and nutrition in fragile and conflict-affected settings. Confl Health. 2022;16(1):30.

16. Adu PA, Stallwood L, Adebola SO, Abah T, Okpani AI. The direct and indirect impact of COVID-19 pandemic on maternal and child health services in Africa: a scoping review. Glob Health Res Policy. 2022;7(1):20.

17. Page MJ, McKenzie JE, Bossuyt PM, Boutron I, Hoffmann TC, Mulrow CD, et al. The PRISMA 2020 statement: an updated guideline for reporting systematic reviews. BMJ (Clinical research ed). 2021;372:n71-n.

18. Page MJ, Moher D, Bossuyt PM, Boutron I, Hoffmann TC, Mulrow CD, et al. PRISMA 2020 explanation and elaboration: updated guidance and exemplars for reporting systematic reviews. BMJ. 2021;372:n160.

19. National Institute for Health Research. READY maternal and new-born health service disruption associated with COVID-19: a systematic review York: PROSPERO; 2022 [Available from: https://www.crd.york.ac.uk/prospero/display_record.php?RecordID=381537https://www.crd.york.ac.uk/prospero/display_record.php?RecordID=381537].

20. Alfirevic Z KE, Dowswell T, et al. Which method is best for the induction of labour? A systematic review, network meta-analysis and cost-effectiveness analysis. (Health Technology Assessment, No. 20.65.) Appendix 2, Search strategy Southampton (UK): NIHR Journals Library (Cochrane Pregnancy and Childbirth Group); 2016 [Available from: https://www.ncbi.nlm.nih.gov/books/NBK379828/.

21. World Health Organization. Global pulse survey on continuity of essential health services during the COVID-19 pandemic Geneva: WHO; 2023 [Available from: https://www.who.int/teams/integrated-health-services/monitoring-health-services/global-pulse-survey-on-continuity-of-essential-health-services-during-the-covid-19-pandemic.

22. World Health Organization. Coronavirus disease (COVID-19) technical guidance: Maintaining Essential Health Services and Systems Geneva: WHO; 2023 [Available from: https://www.who.int/emergencies/diseases/novel-coronavirus-2019/technical-guidance/maintaining-essential-health-services-and-systems.

23. World Health Organization. Quality health services. Geneva: WHO; 2020.

24. Nguyen PH, Sununtnasuk C, Pant A, Tran Mai L, Kachwaha S, Ash D, et al. Provision and utilisation of health and nutrition services during COVID-19 pandemic in urban Bangladesh. Matern Child Nutr. 2021;17(4):e13218.

25. Tadesse E. Antenatal Care Service Utilization of Pregnant Women Attending Antenatal Care in Public Hospitals During the COVID-19 Pandemic Period. Int J Women Health. 2020;12:1181-8.

For peer review only - http://bmjopen.bmj.com/site/about/guidelines.xhtml

26. Tilahun B, Nigusie A, Zelalem M, Mekonnen ZA. Effect of COVID-19 Pandemic on Maternal and Child Health Services and Strategies for Effective Service Implementation in Ethiopia. J Multidiscip Healthc. 2022;15:2781-95.

27. Avula R, Nguyen PH, Ashok S, Bajaj S, Kachwaha S, Pant A, et al. Disruptions, restorations and adaptations to health and nutrition service delivery in multiple states across India over the course of the COVID-19 pandemic in 2020: An observational study. PLoS ONE. 2022;17(7):e0269674.

28. Padhye R, Purushotham A, Paul M, Sardeshpande N, Ballala R, Dhar S, et al. Accessing Maternal Health Care in the Midst of the COVID-19 Pandemic: A Study in Two Districts of Assam, India. Front. 2022;3:750520.

29. Sahoo KC, Doley C, Negi S, Das S, Verma P, Kanungo S, et al. Experiences of Urban Slum-Dwelling Women With Maternal and Child Health Services During COVID-19 Pandemic: A Multi-City Qualitative Study From India. Int J Public Health. 2022;67:1604348.

30. Shrivastava S, Rai S, Sivakami M. Challenges for pregnant women seeking institutional care during the COVID-19 lockdown in India: A content analysis of online news reports. Indian J Med Ethics. 2021;VI(3):1-24.

31. Sinha B, Dudeja N, Mazumder S, Kumar T, Adhikary P, Roy N, et al. Estimating the Impact of COVID-19 Pandemic Related Lockdown on Utilization of Maternal and Perinatal Health Services in an Urban Neighborhood in Delhi, India. Front. 2022;3:816969.

32. Ulaganeethi R, Dorairajan G, Ramaswamy G, Thekkur P, Olickal JJ, Rajkumari N, et al. 'I was scared I will end up in another abortion': a mixed-methods study assessing the impact of COVID-19 pandemic and lockdown on the antenatal care of pregnant women in Puducherry, South India. Fam Pract. 2021;38(Suppl 1):i23-i9.

33. Hazfiarini A, Zahroh RI, Akter S, Homer CSE, Bohren MA. Indonesian midwives' perspectives on changes in the provision of maternity care during the COVID-19 pandemic: A qualitative study. Midwifery. 2022;108:103291.

34. Widiasih R, Hidayat D, Zakaria H, Utama DQ, Komariah M, Maryam NNA, et al. Self-Fetal Wellbeing Monitoring and Ante-Natal Care during the COVID-19 Pandemic: A Qualitative Descriptive Study among Pregnant Women in Indonesia. Int J Environ Res Public Health. 2021;18(21):06.

35. Mirzakhani K, Shoorab NJ, Akbari A, Khadivzadeh T. High-risk pregnant women's experiences of the receiving prenatal care in COVID-19 pandemic: a qualitative study. BMC Pregnancy Childbirth. 2022;22(1):363.

36. Rezapour R, Dorosti AA, Farahbakhsh M, Azami-Aghdash S, Iranzad I. The impact of the Covid-19 pandemic on primary health care utilization: an experience from Iran. BMC Health Serv Res. 2022;22(1):404.

37. Ashish KC, Peterson SS, Gurung R, Skalkidou A, Gautam J, Malla H, et al. The perfect storm: Disruptions to institutional delivery care arising from the COVID-19 pandemic in Nepal. J. 2021;11:05010.

For peer review only - http://bmjopen.bmj.com/site/about/guidelines.xhtml

38. Basnet B, Chapagain P, Subedi S, Dahal T, Neupane S, Khanal R, et al. Experiences of nurses providing maternity care in a public hospital during the COVID-19 pandemic in Nepal: a qualitative study. PLoS Global Public Health. 2022;2(4).

39. Akaba GO, Dirisu O, Okunade KS, Adams E, Ohioghame J, Obikeze OO, et al. Barriers and facilitators of access to maternal, newborn and child health services during the first wave of COVID-19 pandemic in Nigeria: findings from a qualitative study. BMC Health Serv Res. 2022;22(1):611.

40. Adelekan B, Goldson E, Abubakar Z, Mueller U, Alayande A, Ojogun T, et al. Effect of COVID-19 pandemic on provision of sexual and reproductive health services in primary health facilities in Nigeria: a cross-sectional study. Reprod Health. 2021;18(1):166.

41. Leung C, Olufunlayo T, Olateju Z, MacArthur C, Taylor B. Perceptions and experiences of maternity care workers during COVID-19 pandemic in Lagos State, Nigeria; a qualitative study. BMC Health Serv Res. 2022;22(1):606.

42. Mohammed BA, Abubakar D, Muhammad SA, Aremu Y, Onazi SO, Akeredolu F, et al. Impact of COVID-19 on maternal newborn child health services in Federal Medical Centre Gusau, Zamfara State. Science World Journal. 2022;17(1):124-9.

43. Musiimenta A, Tumuhimbise W, Atukunda EC, Ayebaza S, Kobutungi P, Mugaba AT, et al. Challenges in accessing maternal and child health services during COVID-19 and the potential role of social networking technologies. Digit Health. 2022;8:20552076221086769.

44. Nakate MG, Mackay S, Ndirangu-Mugo E, Fleming V. Experiences of mothers and significant others in accessing comprehensive healthcare in the first 1000 days of life post-conception during COVID-19 in rural Uganda. BMC Pregnancy Childbirth. 2022;22(1):938.

45. Psbi Formative Research Study Group. Barriers to optimal care and strategies to promote safe and optimal management of sick young infants during the COVID-19 pandemic: A multi-country formative research study. J. 2022;12:05023.

46. Villalobos Dintrans P, Maddaleno M, Granizo Roman Y, Valenzuela Delpiano P, Castro A, Vance C, et al. [Disruption of health services for pregnant women, newborns, children, adolescents, and women during the COVID-19 pandemic: ISLAC 2020 ProjectInterrupcao dos servicos de saude para gravidas, recem-nascidos, criancas, adolescentes e mulheres durante a pandemia de COVID-19: projeto ISLAC 2020]. Rev Panam Salud Publica. 2021;45:e140.

47. Asefa A, Semaan A, Delvaux T, Huysmans E, Galle A, Sacks E, et al. The impact of COVID-19 on the provision of respectful maternity care: Findings from a global survey of health workers. Women Birth. 2022;35(4):378-86.

48. Rao SPN, Minckas N, Medvedev MM, Gathara D, Y NP, Seifu Estifanos A, et al. Small and sick newborn care during the COVID-19 pandemic: global survey and thematic analysis of healthcare providers' voices and experiences. BMJ Global Health. 2021;6(3):03.

For peer review only - http://bmjopen.bmj.com/site/about/guidelines.xhtml

49. Saso A, Skirrow H, Kampmann B. Impact of COVID-19 on Immunization Services for Maternal and Infant Vaccines: Results of a Survey Conducted by Imprint-The Immunising Pregnant Women and Infants Network. 2020;8(3):22.

50. Semaan A, Dey T, Kikula A, Asefa A, Delvaux T, Langlois EV, et al. "Separated during the first hours" - postnatal care for women and newborns during the COVID-19 pandemic: a mixed-methods cross-sectional study from a global online survey of maternal and newborn healthcare providers. PLoS Global Public Health. 2022;2(4).

51. Turcotte-Tremblay A-M, Leerapan B, Akweongo P, Amponsah F, Aryal A, Asai D, et al. Tracking health system performance in times of crisis using routine health data: lessons learned from a multicountry consortium. Health Research Policy and Systems. 2023;21(1):14.

52. World Health Organization. COVID-19 Strategic Preparedness and Response Plan Operational Planning Guideline. Geneva: WHO; 2021.

53. Fleming P, O'Donoghue C, Almirall-Sanchez A, Mockler D, Keegan C, Cylus J, et al. Metrics and indicators used to assess health system resilience in response to shocks to health systems in high income countries-A systematic review. Health Policy. 2022;126(12):1195-205.

54. World Health Organization. Community needs, perceptions and demand: community assessment tool: A module from the suite of health service capacity assessments in the context of the COVID-19 pandemic. Geneva: WHO; 2021.

55. World Health Organization. Data availability for assessing disruption of essential services for maternal, newborn, child and adolescent health during COVID-19: lessons learned from Tajikistan. Geneva: WHO; 2022.

For peer review only

ACKNOWLEDGEMENTS

We would like to thank Dr Louisa Baxter for providing guidance on clinical and programming quality. Thank you to Dr Alicia Renedo for coding the Spanish language source. Thank you also to Dr Shirley Huchcroft for her invaluable feedback on an early draft of this paper.

For peer review only

For peer review only - http://bmjopen.bmj.com/site/about/guidelines.xhtml

FIGURE 1: PRISMA flow chart

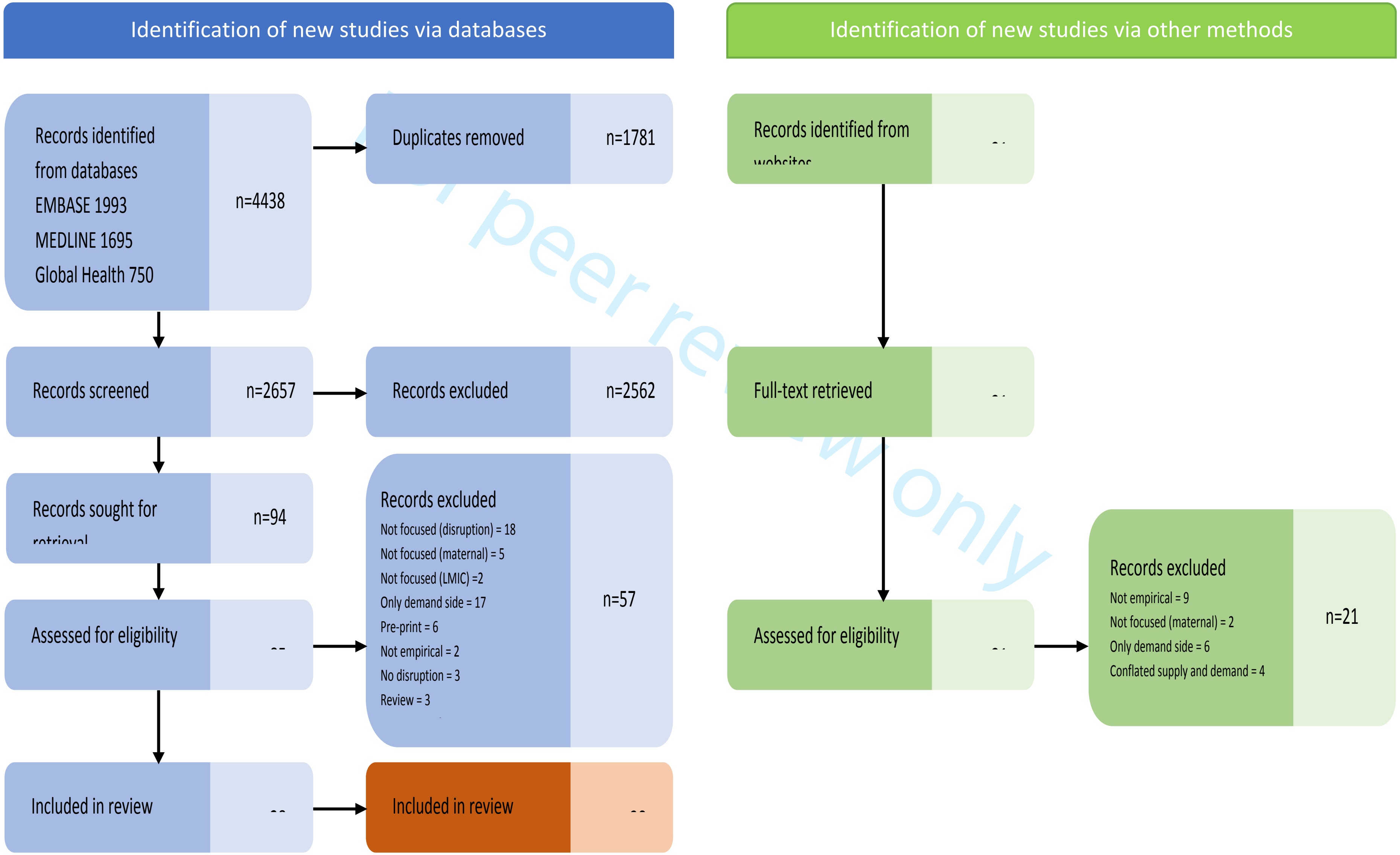

For peer review only - http://bmjopen.bmj.com/site/about/guidelines.xhtml

TABLE 1: MEASURES OF DISRUPTION

	PROVISION OF SERVICES	QUALITY OF SERVICES
QUALITATIVE METHODS	• Semi-structured interviews with health care workers and/or service users asking about difficulties providing care (26, 28, 29, 31, 32, 34, 35, 37, 38, 40, 41, 42, 43, 44)<br>• Free-text field in a survey (48, 49)<br>• Semi-structured interviews with health care workers asking how they provided care at T1 (retrospective baseline) and how they currently (T2) provide care (33)<br>• Semi-structured interviews with health care workers which ask about impacts at various levels of the health system (45)<br>• Review of news articles reporting on disruption (30)	• Semi-structured interviews with health staff and/or service users (26, 28, 31, 35, 37, 38, 39, 41, 42, 44, 48, 49)<br>• Surveys of health workers which include a free-text field asking to what extent has care been provided at T1 (retrospective baseline) compared to T2 (47)<br>• Semi-structured interviews with health care workers which ask about impacts at various levels of the health system (45)<br>• Review of news articles reporting on disruption (30)

For peer review only - http://bmjopen.bmj.com/site/about/guidelines.xhtml

**QUANTITATIVE METHODS**	• Observation of health facilities (to determine number of health care workers on wards, days service disrupted) (37)<br>• Surveys of health care workers which ask respondents to provide a 'disruption score' (13)<br>• Surveys of health care workers which ask about service provision at T1 (retrospective baseline) and T2 (27)<br>• Surveys of health care workers, asking about service provision, taken at baseline (T1), and repeated at T2 and T3 (24)<br>• Surveys of health care workers about disruptions (46, 49, 50)<br>• Surveys of service users (via SMS) asking them to indicate if they received the health service for which they attended the facility (36)<br>• Survey of service users asking them if they missed a health care service and if yes then they are asked to indicate why from a list of facility-related factors (25)	• Surveys of health care workers, asking about service quality, taken at baseline (T1), and repeated at T2 and T3 (24)<br>• Surveys of health care workers about disruptions (50)

TABLE 2: INDICATORS

AUTHOR	LOCATION	DESIGN	MEASURE	POPULATION	INTERVENTION	INDICATOR	EXAMPLE	NOTES
Ashish (37)	Nepal	Observation of clinical services	Extent of disruption	Service-providers	Maternity services	Measured the mean difference of the number of health workers in the labour room and the number of days that maternity services were disrupted between the two time periods (pre-	e.g. mean number of days (4.3) that maternity services were halted during the pandemic and concluded that the number of health care workers (per 24 hours in the labour and delivery room) had decreased to	

For peer review only - http://bmjopen.bmj.com/site/about/guidelines.xhtml

						pandemic and pandemic).	5.4 (from 6.2 before the pandemic) health workers due to redeployment to COVID-19 dedicated care.	
Assefa (13)	Burkina Faso, Ethiopia, Nigeria	Cross-sectional survey	Extent of disruption	Service-providers	General (includes iron and folic acid, ANC, and family planning services)	Measure of the subjective assessment of the categorical level of service disruption presented as a mean interruption score.	e.g. maternal and reproductive services were scored an average of 2.24 out of six [six = total disruption] for Nigeria, 1.72 out of six for Burkina Faso, and 1.67 out of six for Ethiopia	
Avula (27)	India	Cross-sectional survey (asking about T1 and T2) and routine HIS data on service provision (T0)	Extent of disruption	Service-providers	Iron and folic acid, and ANC	Measure of changes in service provision (categorically defined at T1 and T2 as proportion of women provided with ANC services, and at T0 as the number of women receiving services) across the pre-pandemic, lockdown, and post-lockdown periods.	e.g. ANC services were disrupted during the lockdown period but were restored during the post-lockdown period (but not to pre-pandemic levels).	
Nguyen (24)	Banglades h	Longitudinal survey (at T0, and repeated at T2 [at which time participants asked to recall services	Extent of disruption	Service-users and service-providers	Iron and folic acid, and ANC/PNC	Proportionate measure of service provision (defined as the proportion of service-providers indicating provision of specific	e.g. fewer facilities offered ANC services during lockdown when compared to before the pandemic (dropping 6.6 percentage points), and that there	

For peer review only - http://bmjopen.bmj.com/site/about/guidelines.xhtml

		provided in the month preceding the survey (T2) and during lockdown (T1)]				services, and the proportion of service-users indicating receipt of specific services) at pre-pandemic (T0), lockdown (T1), and post lockdown (T2).	were significant drops in the receipt of anthropometric measurements, and iron/folic acid/calcium supplementation. All services recovered post-lockdown but not to the pre-pandemic levels.	
Rezapour (36)	Iran	Survey	Extent of disruption	Service-users	General (includes midwifery services)	Proportionate measure of services received as a factor of total presentations, by month.	e.g. the study found that there was a small but significant drop in the ASPD for midwifery services when comparing the pandemic to the pre-pandemic periods.	
Saso (49)	51 countries	Cross-sectional survey	Extent of disruption	Service-providers	Maternal and infant vaccination services	Proportionate measure of subjective assessment of disruption to vaccine delivery across several countries.	e.g. 53% of LMIC-based respondents reported disruptions to maternal immunisation services compared to 42% of HIC-based respondents.	
Semaan (50)	61 countries	Cross-sectional survey	Causes of disruption	Service-providers	Maternal and newborn post-natal care	Proportionate measures of the subjective assessment of the causes of disruption (in terms of the provision and quality of services) to post-natal	e.g. respondents from LMICs reported various disruptions due to COVID-19 including reduced numbers of beds due to social distancing (37%), cessation of home visits	The survey categories describing disruption were identified from previous

For peer review only - http://bmjopen.bmj.com/site/about/guidelines.xhtml

						care across multiple countries.	(20%), and suspension of post-natal care (8%).	survey rounds.
Tadesse (25)	Ethiopia	Cross-sectional survey	Causes of disruption	Service-users	ANC services	Proportionate measure of disrupted (delayed or missed) ANC visit as well as a proportionate subjective assessment of the causes of the disruption.	e.g. 216 respondents reported missed or late ANC visits, of which 72 (33.33%) attributed this to diversion of maternal services to COVID-19.	It is unclear where the survey categories of disruption have come from.
Villalobos Dintrans (46)	Latin America and Caribbean	Cross-sectional survey	Extent of disruption	Service-providers	General (includes MNH services)	Proportionate measure (using a Likert scale: suspended, partially reduced, same as before, adapted to circumstances, new service created) of the perception of the impact of COVID-19 on health during the pandemic.	e.g. services for newborns and pregnant women — such as institutional delivery care and postpartum care—, as well as vaccination programs, showed the best perceptions of coverage during the pandemic.	

For peer review only - http://bmjopen.bmj.com/site/about/guidelines.xhtml

APPENDIX 1: Search Strategy

Database	MNH	Service	COVID-19
MEDLINE	maternal OR pre?natal OR peri?natal or peri?partum OR ante?natal OR post?natal OR postpartum OR pregnan* OR breastf* OR babo?r OR midwife* OR “birth attendant*” OR “attended birth*” OR new?born [TI/AB]<br><br>maternal health services [MeSH]	service OR program* [TI/AB]	COVID-19 OR 2019-nCoV OR SARS-CoV-2 OR coronavirus OR COVID OR COVID19 [TI/AB]<br><br>covid-19 [MeSH]
EMBASE	maternal OR pre?natal OR peri?natal or peri?partum OR ante?natal OR post?natal OR postpartum OR pregnan* OR breastf* OR babo?r OR midwife* OR “birth attendant*” OR “attended birth*” OR new?born [TI/AB]	service OR program* [TI/AB]	COVID-19 OR 2019-nCoV OR SARS-CoV-2 OR coronavirus OR COVID OR COVID19 [TI/AB]
Global Health	maternal OR pre?natal OR peri?natal or peri?partum OR ante?natal OR post?natal OR postpartum OR pregnan* OR breastf* OR babo?r OR midwife* OR “birth attendant*” OR “attended birth*” OR new?born [TI/AB]	service OR program* [TI/AB]	COVID-19 OR 2019-nCoV OR SARS-CoV-2 OR coronavirus OR COVID OR COVID19 [TI/AB]

For peer review only - http://bmjopen.bmj.com/site/about/guidelines.xhtml

APPENDIX 2: INCLUDED STUDIES

AUTHOR	YEAR	AIM	METHOD	SETTING	DATA COLLECTED	FOCUS	FINDINGS
Adelekan (40)	2021	"…to investigate the extent to which the COVID-19 pandemic and related lockdowns had affected the provision of essential reproductive, maternal, child, and adolescent health (RMCAH) services in primary health care facilities across the Nigerian States" (p. 01).	**QUALITATIVE**<br>Semi-structured interviews (questionnaire not provided)<br>Description of questions: description of the health facility, RMCAH service delivery and use, and difficulties experienced before/during/after lockdown.<br>Head nurses and midwives	**NIGERIA**<br>Primary Health Centres (PHCs) in selected Local Government Areas (LGAs) in 10 states: Lagos, Akwa Ibom, Kano, Kaduna, Gombe, Borno, Ogun, Enugu, Adamawa, and the Federal Capital Territory (FCT) (Abuja Municipal Area Council)	**2020**<br>1 Nov-16 Dec	**RMCAH**	**PROVISION OF SERVICE**<br>**Defined as: Percentage of surveyed PHC's offering a service**<br>ANC: No decline in provision of service except in Gombe (10% decrease during lockdown)<br>Delivery care: No decline in provision of service.<br>Intrapartum care: No decline in provision of service except in Gombe (increased during lockdown but following lockdown dropped to pre-lockdown levels).<br>Postnatal care: Decline in provision of service during lockdown (for some states) and further decline following lockdown (for some states).<br>Childhood immunisation: Decline during lockdown and further decline following lockdown (except in Borno)
Akaba (39)	2022	"…to explore the barriers and facilitators of access to MNCH services during the first wave of COVID-19	**QUALITATIVE**<br>In-depth interviews (questionnaire provided)<br>Used 'three delays model' to structure interviews (i.e.	**NIGERIA**<br>Six states: Abuja, Lagos, Kano (high numbers of cases); Engu, Taraba,	**2020**<br>May/Jul	**MNH**	**QUALITY OF SERVICE**<br>• Delays in receiving care<br>• Shortage of health staff<br>• Inadequate supplies

For peer review only - http://bmjopen.bmj.com/site/about/guidelines.xhtml

		pandemic in Nigeria" (p. 01).	reason for delay in seeking care, delay in reaching health care facility, delay in receiving care at the hospital).<br>Service users, health workers, policymakers	Bayelsa (lower numbers of cases)			• Lack of preparedness amongst staff
Asefa ([47])	2022	"…to explore how the COVID-19 pandemic negatively affected frontline health workers' ability to provide respectful maternity care globally" (p. 378).	**QUALITATIVE**<br>Survey data [free-text responses] (questionnaire not provided)<br>Description of question: '*At this time, to what extent do you feel that you are able to provide respectful care to women and newborns compared to before the COVID-19 outbreak?*'<br>Health workers (mainly midwives, obstetricians, gynaecologists, nurses, and GPs)	**71 COUNTRIES**<br><br>Includes LMICs and HICs	**2020**<br><br>Jul-Dec	**Respectful maternity care**	**QUALITY OF SERVICE**<br>Six ways in which the provision of care was negatively affected:<br>• Less family involvement<br>• Reduced emotional support to women<br>• Reduced physical support for women<br>• Compromised standards of care<br>• Increased risk of medically unjustified caesarean section<br>• Overwhelmed staff with rapidly changing guidelines and enhanced infection prevention measures
Ashish ([37])	2021	"…to understand the disruption of maternity services in Nepal due to the COVID-19 pandemic as well as to explore the provision and experience of	**BOTH**<br>**Quantitative**<br>Observation [number of health care workers in the labour and delivery room and those re-deployed to COVID-19 area; number of	**NEPAL**	2019/20<br><br>Mar-Aug 2019, Mar-Aug 2020	**Respectful maternity care**	**PROVISION OF SERVICE**<br>• Maternity services halted for mean 4.3 days during pandemic<br>• Redeployment of staff to COVID-19 care<br>• Positive

For peer review only - http://bmjopen.bmj.com/site/about/guidelines.xhtml

		institutional delivery care" (p. 2).	days maternity services were disrupted]<br>**Qualitative**<br>Semi-structured interviews (questionnaire not provided)<br>Mothers with live births (followed-up 45 days post-partum)				• Reduced number of health care workers in labour room (compared to pre-COVID)<br>**QUALITY OF SERVICE**<br>• Less than half were counselled to keep newborn warm (40%), or exclusive breastfeeding (44%).<br>• Few were counselled on newborn danger signs (15%)<br>• Consent before vaginal examination (primiparous 55%; nulliparous 44%)
Assefa (13)	2021	"…to characterize the impacts of the COVID-19 pandemic on the interruptions on health services from the perspectives of both HCPs and community members in three sub-Saharan African countries, Burkina Faso, Ethiopia, and Nigeria" (p. 324).	**QUANTITATIVE**<br>Survey data [interruption of health service by type] (questionnaire not included)<br>Questions related to the interruption of a type of health service were scored from 0 to 2 (0 = no interruption;1 = partial interruption; 2 = complete interruption). Asked to score: ANC, iron and folate, family planning.<br>Health care workers (also surveyed community to assess access – not included)	**BURKINA FASO, ETHIOPIA, NIGERIA**	**2020**<br>Jul-Nov	**General (but includes maternal and reproductive health services)**	**PROVISION OF SERVICE**<br>• Almost all services were reported to have experienced disruption<br>• Maternal and reproductive services particularly interrupted<br>• MRH: Nigeria (2.24 out of 6); Burkina Faso (1.72 out of 6); and Ethiopia (1.67 out of 6). [where 6=complete interruption]. Total (1.85 out of 6).<br>• Total health service interruption: Nigeria (7.9 out of 20); Burkina Faso (6.2 out of 20); and Ethiopia (5.4 out of 20). [where 20=complete interruption]. Total (6.49 out of 20)
Avula (27)	2022	to, "…stud[y] disruptions and restorations, challenges and adaptations in health and nutrition	**QUANTITATIVE**<br>Survey data [service delivery at T1-lockdown and T2-post-	**INDIA**<br>Seven states: Bihar, Chhattisgarh, Madya Pradesh, Odisha, Tamil	**2020**<br>Aug-Oct	**FLW-provided services (iron and folic acid, and ANC)**	**PROVISION OF SERVICE**<br>• Services were disrupted during T1-lockdown but were restored in the

For peer review only - http://bmjopen.bmj.com/site/about/guidelines.xhtml

		service delivery by frontline workers (FLWs) in India during COVID-19 in 2020" (p. 1).	lockdown] (questionnaire included)<br>Question example:<br>a. "Were you able to provide help in providing antenatal care services to all pregnant women?" (Yes/No)<br>b. "…in the last month?" (0=No one; 1=Yes, only to some pregnant women in the last trimester for high-risk pregnant women; 2=Yes, to all pregnant women)<br>c. "…during the last COVID-19 lockdown in April?"<br>HIS data also retrieved to establish T0-pre-COVID-19 service provision.<br>Frontline workers	Nadu, Telangana, and Uttar Pradesh			T2-post lockdown (but not to T0-pre-pandemic levels)
Basnet (38)	2022	"…to explore the experiences of nurses providing maternity care in the public sector during the COVID-19 pandemic in Nepal" (p. 01).	**QUALITATIVE**<br>Semi-structured interviews (topic guide not provided)<br>Topic guide was developed based on previous relevant literature, the research question, and informal interviews.<br>Nurses	**NEPAL**<br>Biratnagar (Koshi Hospital)	**2020**<br>Sep-Nov	**MNH**	**QUALITY OF SERVICE**<br>• The majority of the 10 participants had experienced decreased quality of care<br>• Decrease in nurse-patient time<br>• Decrease in presence of medical practitioners<br>• Time allocated per patient decreased<br>**PROVISION OF SERVICE**<br>• Reduced kangaroo mother care for pre-term<br>• Counselling less consistent

For peer review only - http://bmjopen.bmj.com/site/about/guidelines.xhtml

Hazfiarini (33)	2022	"…[t]o explore how COVID-19 influenced the provision of high-quality maternity care in Indonesia" (p. 01).	**QUALITATIVE**<br>Semi-structured interviews (topic guide not provided)<br>Topic guide included three sections: midwives were asked how they provided antenatal, childbirth, postpartum care prior to COVID-19 [Q1] and during COVID-19 [Q2], and were asked to describe experiences providing care during COVID-19 [Q3].<br>Midwives	**INDONESIA**<br>Surabaya and Mataram	**2020/1**<br>Dec 2020 - Feb 2021	**Midwife services**	**PROVISION OF SERVICE**<br>• Cancelled education classes<br>• Reduced frequency of ANC reduced (reducing from 6 to 2-4)<br>• Reduced post-partum visits
Leung (41)	2022	"…to explore maternity healthcare workers' experiences of, and perceptions about providing maternity care during the COVID-19 outbreak in Lagos State, Nigeria" (p. 01).	**QUALITATIVE**<br>Semi-structured interviews (topic guide provided)<br>Topic guide included:<br>"How has COVID-19 outbreak affected maternity care services?<br>What impact do you feel that COVID-19 pandemic has had on your work?<br>Probe: How has COVID-19 affected normal daily tasks/responsibilities?<br>Probe: How has COVID-19 affected the way you look after patients?	**NIGERIA**<br>Seven Local Government Areas in Lagos State (Lagos Mainland, Surulere, Ikeja, Alimosho, Kosofe, Badagry, OshodiIsolo)	**2021**<br>Mar-Apr	**Facility-based maternity care**	**PROVISION OF SERVICE**<br>• Antenatal appointments for non-urgent cases had been reduced<br>• Home visits not possible<br>• Some felt services maintained<br>**QUALITY OF SERVICE**<br>• Some midwives had taken on COVID-19 roles (e.g. vaccination)<br>• Reduced capacity of consultation room

For peer review only - http://bmjopen.bmj.com/site/about/guidelines.xhtml

			Probe: Have supply of drugs, equipment, PPE been affected?<br>Probe: Have staff been redeployed from or within your facility?<br>Please tell me about any changes COVID-19 has had on your work<br>Probe: Can you explain why there have been these changes?<br>Probe: How do you feel about the changes you have had to make during the pandemic?<br>Probe: COVID-19 control measures - How do you feel about mask wearing/social distancing/handwashing?<br>What differences have you noticed in the way that patients interact with your services during COVID-19?<br>Probe: Has there been a change? Why?<br>Midwives and TBAs				
Mirzakhani (35)	2022	"…to explore the experiences of women with high-risk pregnancies who were receiving prenatal care	**QUALITATIVE**<br>Semi-structured interviews (no topic guide provided)<br>Topic guide included:	**IRAN**<br>Ghaem, Ommolbanin, and Imam Reza in Mashhad	**2020/2**<br>Sep 2020 – Mar 2021	**Women with high-risk pregnancies**	**QUALITY OF SERVICE**<br>• Health care provider's care and attention had decreased during COVID-19

For peer review only - http://bmjopen.bmj.com/site/about/guidelines.xhtml

		during the COVID-19 pandemic" (p. 01).	1. What was your experience with prenatal care during the Corona pandemic?<br>2. How do you feel about being pregnant in this situation?<br>3. How do you feel when you apply for prenatal care?<br>4. How is your access to prenatal care in the face of the Corona pandemic?<br>5. Discuss the factors that make you feel good about receiving health services.<br>6. Discuss the factors that make you feel uneasy/bad about receiving health services.<br>7. How does the environment and location of health delivery affect health services receipt?<br>8. What limitations /restrictions did you face when seeking health services?<br>Women with high-risk pregnancies				• Staff refused to listen to foetal sounds or perform abdominal examinations<br>• Shorter visits from health care workers<br>• Staff not taking time to talk<br>• Mothers reported stress, anxiety, neglect<br>**PROVISION OF SERVICE**<br>• Some doctors closed their offices<br>• Drug shortages
Mohammed (42)	2022	"…[to] assess the effect of Covid-19 on maternal and child health services in Federal medical Centre	**QUALITATIVE**<br>Key informant interviews (no topic guide provided)	**NIGERIA**	??	**MNH services**	**PROVISION OF SERVICE**<br>• Some respondents reported having to quarantine after contact with COVID-19 positive patient

For peer review only

		Gusau Zamfara state following the lockdown" (p. 124).	Topic guide was 'standard COVID-19 KII guide' and asked respondents' opinion on the COVID-19 outbreak and its specific and general effects on the facility.<br>Heads of units delivering MNCH services (principal medical officers, chief nursing officers, principal nursing officers and nutrition officers)	Federal medical centre Gusau, Zamfara state			• Most participants believed services were affected with some suspended<br>**QUALITY OF SERVICE**<br>• Paediatric outpatient and paediatric emergency unit reduced staffing numbers
Musiimenta (43)	2022	"…[to] describe the challenges experienced in accessing maternal and child health services by women with limited or no education during this COVID-19 pandemic and discuss the potential of social network technologies (SNTs) to support maternal and child health amidst this crisis".	**QUALITATIVE**<br>Interviews (no topic guide provided)<br>Interviews meant to elicit information about challenges women encountering in accessing maternal and child health services during lockdown.<br>Women who had recently given birth in a rural setting	**UGANDA**<br>Mbarara Regional Referral Hospital	**2021**<br>Jul	**Post-natal care**	**PROVISION OF SERVICE**<br>• Delays in accessing services, long queues and missed appointments
Nakate (44)	2022	"…[to] explore the experiences of mothers and their significant others of comprehensive care in the first 1000 days of life post-conception	**QUALITATIVE**<br>Semi-structured interviews (no topic guide provided)<br>Topics included:<br>Open-ended questions regarding participants' experiences accessing care	**UGANDA**<br>Bunghokho-Mutoto sub-county, Mbale District	**2020**<br>10 Oct-9 Nov	**First 1000 days post-conception**	**PROVISION OF SERVICE**<br>• Lack of follow-up visits<br>• Curfew resulted in staff starting late and leaving early whilst mothers waited

For peer review only - http://bmjopen.bmj.com/site/about/guidelines.xhtml

		during the COVID-19 pandemic" (p. 01).	and their understanding of these experiences.<br>Mothers (pregnant or with a child under 2 years) and their significant others				• Facilities reduced admissions or appointments due to staff shortages<br>**QUALITY OF SERVICE**<br>• Shortages of staff and work overload was felt to have hindered provision of care<br>• Staff pre-occupied with infection prevention and control
Nguyen (24)	2021	"…to (1) quantify the effect of the COVID-19 pandemic and COVID-19 restrictions on health and nutrition service provision and utilisation by pregnant women and mothers of children under 2 years of age in Dhaka City, Bangladesh, and (2) identify adaptions and a range of feasible solutions that have the potential to strengthen delivery and uptake of essential health and nutrition interventions in the context of COVID-19 and beyond" (p. 03).	**QUANTITATIVE**<br>Survey data [to assess survey provision before pandemic, during lockdown, and post-lockdown]<br>(survey not included)<br>Survey (health providers):<br>1) Feb baseline: assess exposure to nutrition training, workload and time commitments, and services provided.<br>2) Sep/Oct follow-up: assess trainings, receipt of PPE, additional responsibilities, service provision, and challenges and adaptations (asked to recall Mar-May [lockdown] and Sept)<br>Survey (mothers):<br>1) baseline: assess receipt of health and nutrition services	**BANGLADESH**<br><br>Dhaka City	**2020**<br><br>Sept-Oct	**Mothers with children <2**	**PROVISION OF SERVICE**<br>• Fewer facilities offered ANC services during lockdown when compared to before pandemic (dropping 6.6 percentage points)<br>**QUALITY OF SERVICE**<br>• Most ANC services still offered (with the exception of anthropometric measurements (20 percentage point drop for weight measurements), (29 percentage point drop for height measurements)<br>• Post-lockdown anthropometric measurements were higher post-lockdown but still below pre-pandemic levels<br>• Smaller drops reported for iron folic-acid and calcium supplementation (<4 percentage points), nutrition counselling (dropped to 12.5 percentage points post-lockdown when compared to lockdown)

For peer review only - http://bmjopen.bmj.com/site/about/guidelines.xhtml

			2) follow-up: how service use had changed, exposure to services, counselling messages, mode of service receipt and challenges<br>**Included a baseline survey**<br>Health providers and mothers with children under 2				• Other services affected but all improved post-lockdown though not to pre-pandemic levels<br>• Increased workloads during the pandemic was reported by 56% of providers.
Padhye (28)	2022	"…to understand the status of maternal health service provision and challenges faced by 110 pregnant and recently delivered women, 38 health care providers and 18 Village Health Sanitation and Nutrition Committee members during COVID-19 pandemic" (p. 01).	**QUALITATIVE**<br>Interviews<br>(topic guide not included)<br>Interviews were intended to: understand the effect of COVID-19 and lockdown on service provision (for health care providers) and understand the role and capacity building efforts for providing access to maternal health services (VHSNC) in their village.<br>Health care providers and Village Health Sanitation and Nutrition Committee members (VHSNC)	**INDIA**<br>Two districts in Assam (Kamrup and Darrang)	**2020**<br>Oct-Dec	**Maternal health services**	**PROVISION OF SERVICE**<br>• Most ANC/PNC services were managed through home visits but travel to remote areas was difficult<br>• Non-availability of laboratory services was a significant gap in ANC provisioning<br>• Village-level health centres were closed for three months during lockdown<br>• Post-lockdown iron and folic acid tablets were unavailable at the village health centres<br>• Village Accredited Social Health Activists reported not being able to provide any of the expected health services during the pandemic<br>• Services that were not available included ultrasound check-up, laboratory services, health care providers, and certain health facilities

For peer review only - http://bmjopen.bmj.com/site/about/guidelines.xhtml

							**QUALITY OF SERVICES**<br>• Home deliveries for high-risk women were supported by health care staff but without backup support from a medical officer or an auxiliary nurse midwife
PSBI Formative Research Study Group (45)	2022	"…to understand the pandemic's impact on barriers to and mitigation for strategies of care-seeking and managing possible serious bacterial infection (PSBI) in young infants" (p. 01).	**QUALITATIVE**<br>Observation, in-depth interviews, and focus groups (to ascertain trends in service provision and use across lockdown and post lockdown periods)<br>(conceptual framework included)<br>Conceptual framework lists factors influencing PSBI management:<br>**Changes in National and local policies**<br>• Conversion of hospitals into COVID-19 care centres<br>**COVID-19 care centres**<br>• Resource reallocation • Task shifting • Diversion of cases to other higher or lower health care facilities.<br>**Health Systems changes: public and private; facility and community**<br>• Functionality of outpatient and inpatient facilities •	**BANGLADESH, ETHIOPIA, INDIA, NIGERIA, PAKISTAN**	**2020/1**<br>Dec 2020 – Mar 2021	**Newborn care**	**PROVISION OF SERVICE**<br>• Several hospitals were closed, offered restricted services, or converted to COVID-19 care centres<br>• Respondents discouraged the admission of infants except in an emergency<br>• Many of these problems reported to have existed pre-COVID-19<br>• Community clinics closed during lockdown<br>• Routine services (e.g. ANC, vaccination, and post-partum care) were disrupted<br>• Outpatient care was disrupted at many sites due to service suspension, limited hours, or non-availability of staff<br>• Home-based post-natal and referral were also reduced<br>**QUALITY OF SERVICES**<br>• Some facility staff indicated that staff shortages due to COVID-19, insufficient oxygen and medicines, and unhygienic conditions at

For peer review only - http://bmjopen.bmj.com/site/about/guidelines.xhtml

			Effective referral system • Availability of human resources • Availability of essential equipment, supplies and medicines • Perceptions, fear and apprehensions among health care providers and community health workers • Availability of COVID-related supplies<br>**Caregivers and Community**<br>• Fear of accessing care • Needs for seeking treatment outside the home or allowing health personnel to visit home • Constraints in accessing treatment • Transportation availability, costs • Perceived barriers • Immediate competing demands • Economic stress, loss of employment, PSBI low priority<br>Health care staff (CHWs, staff nurses, medical officers, program managers, and private practitioners)				facilities had led to sub-optimal care for young infants<br>• Some respondents reported poor care and limited patient time with staff who were unwilling to touch or examine infants<br>• Many of these problems reported to have existed pre-COVID-19<br>• Social distancing felt to have reduced service quality
Rao (48)	2021	"…to survey reported disruptions to small and sick newborn care worldwide and undertake thematic analysis of healthcare	**QUALITATIVE**<br>Survey data<br>(survey not included)<br>Survey collected free-text data on:	**62 COUNTRIES**<br>mainly LMICs	**2020**<br>13 Jul – 13 Oct	**Newborn care**	**QUALITY OF SERVICES**<br>• Changes to newborn care reported including reallocation of unit space and reassignment of staff from newborn care to COVID-19 and other duties

For peer review only - http://bmjopen.bmj.com/site/about/guidelines.xhtml

		providers' experiences and proposed mitigation strategies to provide insights on disruptions to coverage and quality of small and sick newborn care, and to identify possible solutions to protect vulnerable newborns during the COVID-19 and similar future pandemics" (p. 1).	Demographic characteristics, healthcare preparedness and response to COVID-19, the effect of COVID-19 on health professionals and newborn care, and major disruptions and solutions.<br>Health providers (nurses, midwives, doctors and community health workers), public health professionals and policymakers				• Oxygen supplies for newborns compromised<br>**PROVISION OF SERVICES**<br>• Some respondents reported closure of wards (in this case KMC) due to its proximity to the COVID-19 area<br>• A hospital outbreak resulted in closure of 'mother lodger' and KMC wards<br>• Limited social distancing space affected the quality of neonatal care<br>• Fewer staff to carry out follow-up visits and fewer appointments per newborn<br>• Staff anxiety about infection compromised KMC care
Rezapour (36)	2022	"…[to] investigate the impact of the COVID-19 pandemic on the service utilization status in the Iranian PHC system" (p. 01).	**QUANTITATIVE**<br>Survey of patients<br>(text message included)<br>Indicator Actual Percentage of Service Delivery (ASPD) measured the actual number of people who have gone to PHC facilities and received the service.<br>SMS was sent to all patients via SMS. SMS message read: "Has the service been received?"<br>**Includes baseline survey**	**IRAN**	**2019-2021**<br>Mar 2019-Jan 2020 (before); Feb 2020-Feb 2021 (after)	**General (include midwifery services)**	**PROVISION OF SERVICES**<br>• There was a significant difference in ASPD for midwifery services when comparing pre-pandemic to the COVID-19 period

For peer review only - http://bmjopen.bmj.com/site/about/guidelines.xhtml

			PHC facility patients				
Sahoo ([29](#))	2022	"…[to] explore the experiences of vulnerable communities—urban-slum-dwelling women regarding maternal and child health services during COVID-19" (p. 01).	**QUALITATIVE**<br>In-depth interviews [about experiences including coping strategies, and health system readiness concerning participants' MCH care]<br>(topic guide included)<br>Topic guide questions:<br>Q1. Please share your experience during the COVID-19 pandemic?<br>Q2. What is your experience with availing antenatal services/Intra/postnatal services/immunization services during a regular period in your community? Probe: availability—site, day, time, staff, logistics, transport, appropriateness—technical and professional adequacy, approachability, acceptability, affordability, continuity of services<br>Q3. What changes have you seen in services during the COVID-19 situation? Probe: availability, appropriateness, approachability, acceptability, affordability, continuity of services	**INDIA**<br>Four states: Odisha, Uttarakhand, Chhattisgarh, and Assam.	**2020**<br>Apr-May	**ANC, nutritional care for pregnant women, intra-natal and post-natal care, immunisation, and sick infant treatment**	**PROVISION OF SERVICES**<br>• The majority of respondents reported that they received the antenatal check-up whilst in lockdown<br>• Some intra-natal services were not provided by the facility<br>• Timing of immunisation services were changed to accommodate staff COVID-19 duties

For peer review only - http://bmjopen.bmj.com/site/about/guidelines.xhtml

			Q4. How did you manage the care of your sick infants during the pandemic?<br>Q5. How did you cope with the situation?<br>Q6. In your view, how can we strengthen the services in an emergency?<br>Urban slum dwelling women who were antenatal, intra-natal, or postnatal				
Saso (49)	2020	"…[t]o understand immunization service interruptions specifically for maternal, neonatal and infant vaccines" (p. 01).	**BOTH**<br>Survey data<br>(survey question included)<br>Survey questions:<br>Are you aware of any issues in delivering these vaccines successfully during the COVID-19 pandemic? (Yes/No/Don't know)<br>If you answered yes to the above question, please provide some detail below (e.g. administration problems, rescheduling, staff shortage, parental concerns, social/logistical barriers, others)<br>Health care workers (e.g. doctors, midwives, nurses, lab scientists, public health officials)	**51 COUNTRIES**<br><br>Includes 32 (63%) LMICs	**2020**<br><br>15 Apr-30 Apr	**Maternal and infant vaccination**	**PROVISION OF SERVICE**<br>• 50% of respondents reported disruption in either maternal or infant vaccine delivery (similarly reported across HICs and LMICs)<br>• Disruption newborn vaccination was reported by 42% (n=15) of LMIC-based respondents<br>• Disruption in maternal vaccination was reported by 53% (n=19)<br>• Disruption to clinic services was reported by most participants<br>• Issues secondary to vaccine shortages and logistics primarily described within LMICs<br>**QUALITY OF SERVICE**<br>• Staff shortages were described more frequently in the context of newborn, infant, toddler vaccination

For peer review only - http://bmjopen.bmj.com/site/about/guidelines.xhtml

							• The barriers described in LMICs were more skewed towards supply-side issues
Semaan (50)	2022	"...to explore PNC provision, availability, content and quality following the early phase of the COVID-19 pandemic" (p. 01).	**QUANTITATIVE**<br>Survey data (questions developed iteratively from past surveys)<br>(survey questions provided)<br>Survey questions:<br>In the past month, how was inpatient postnatal care for women and newborns affected? (Please select all that apply)<br>In the past month, how was outpatient postnatal care affected? (Please select all that apply)<br>In the past month, was the content of inpatient postnatal care to women and newborns provided by you or in your facility affected in terms of: (Please select all that apply)<br>In the past month, was the content of outpatient postnatal care to women and newborns provided by you or in your facility affected in terms of: (Please select all that apply)	**61 COUNTRIES**<br>Includes 34 LMICs	**2020**<br>Jul-Dec	**Post-natal care (mothers and newborns)**	**QUALITY OF SERVICE**<br>• Amongst LMIC respondents 37% reported reduced numbers of beds due to social distancing<br>• Amongst LMIC respondents 14% reported reduced space in postnatal ward<br>[ADD FIGURE 4]<br>**PROVISION OF SERVICE**<br>• Amongst LMIC respondents 53% reported that appointments were scheduled further apart<br>• Amongst LMIC respondents 28% reported not being able to provide postnatal care face-to-face for all patients<br>• Amongst LMIC respondents 20 reported that home visits had stopped<br>• Amongst LMIC respondents 8% reported suspension of outpatient postnatal care<br>• Amongst LMIC respondents 22% reported shorter operating hours or fewer days for postnatal care<br>• Amongst LMIC respondents 6% reported suspending inpatient postnatal care<br>[ADD FIGURE 4]

For peer review only - http://bmjopen.bmj.com/site/about/guidelines.xhtml

			Maternal and newborn health providers				
Shrivastava (30)	2021	"…to understand the challenges faced by pregnant women seeking institutional care during the lockdown" (p. 202).	**MEDIA ANALYSIS**<br>Content analysis (News articles reporting on the challenges that pregnant women faced when seeking institutional care during the lockdown)<br>Pregnant women seeking institutional care	INDIA<br><br>17 states	2020<br><br>Mar-May	Pregnant women	**PROVISION OF SERVICE**<br>• Night shift staff were reassigned to other duties to address HR gaps caused by COVID-19<br>**QUALITY OF SERVICE**<br>• Pregnant women experienced poor quality care during lockdown; related to systemic health system issues, or individual care
Sinha (31)	2022	"…[t]o estimate utilization of maternal, perinatal healthcare services after the lockdown was implemented in response to the COVID-19 pandemic compared to the period before" (p. 01).	**QUALITATIVE**<br>Semi-structured survey and in-depth interviews with a sub-sample<br>(no survey questions or topic guide provided)<br>Survey included:<br>Sociodemographic, antenatal, intrapartum and postnatal care details including out-of-pocket expenditure<br>Interview topics:<br>Experiences, challenges, and barriers in accessing health care services.<br>Women who had recently delivered	INDIA<br><br>Delhi	2019/20<br><br>Oct 2019 – Mar 2020 (pre-lockdown)<br><br>Mar 2020 – Nov 2020 (after lockdown)	Maternal and perinatal services	**PROVISION OF SERVICE**<br>• Many hospitals had been converted into CVODI-19 care centres<br>• Respondents reported that community workers were unavailable during lockdown for AND or postnatal services<br>**QUALITY OF SERVICE**<br>ANC (survey)<br>• The most common barriers to availing ANC services were poor quality of care (55%), lack of transport (41%), or long waiting times (39%).<br>ANC (interviews)<br>• Accounts of poor quality care and staff reluctant to carry out examinations<br>Intrapartum care (survey)

For peer review only - http://bmjopen.bmj.com/site/about/guidelines.xhtml

							• 28% of respondents reported issues with intrapartum care<br>Intrapartum care (interviews)<br>• Accounts of fear of contracting COVID-19, poor quality of care, and availability of few or no health staff<br>• Respondents found labour room experience distressing due to shortage of staff and unhygienic facilities
Tadesse (25)	2020	"…[t]o assess the impact of the COVID-19 pandemic on antenatal care utilization among pregnant women attending public facilities in Northeast Ethiopia" (p. 1181).	**QUANTITATIVE**<br>Interviewer-administered questionnaire<br>(questionnaire provided)<br>Did you miss or late to start ANC visit during COVID-19 outbreak? (Yes/No)<br>Facility related factors:<br>1) deploying of maternal care workers (Yes/No)<br>2) interruption and diversion of maternity services to COVID-19 response (Yes/No)<br>3) Fear of COVID-19 infection (Yes/No)<br>4) Social distancing (Yes/No)<br>Pregnant women	**ETHIOPIA**	**2020**<br>Feb-Aug	**ANC**	**PROVISION OF SERVICE**<br>• Interruption and diversion of maternal health services due to COVID-19 reduced full service use by 32%
Tilahun (26)	2022	"…to examine the effect of the COVID-19 pandemic on MNCH	**QUALITATIVE**<br>In depth interviews	**ETHIOPIA**	**2020**	**Maternal and child health services**	**PROVISION OF SERVICE**<br>• Respondents reported low commitment of health care

For peer review only

For peer review only - http://bmjopen.bmj.com/site/about/guidelines.xhtml

		service uptake and to identify effective strategies to ensure MNCH service delivery during the pandemic in Ethiopia" (p. 2782).	(no interview guide; no description of interview guide)<br>Decision-makers, health workers, NGO delegates and clients				workers, movement restrictions, and shortage of resources were felt to have reduced the quality of services
Ulaganeethi (32)	2021	"…to assess the proportion of pregnant women who had not completed the ideal number of antenatal visits, availability of iron-folic acid (IFA) supplements and challenges in availing health services during the period of lockdown" (p. i23).	**QUALITATIVE**<br>Semi-structured interviews (Interview guide provided)<br>Interviews included:<br>3. Did you face any difficulty in availing antenatal care services or healthcare services for any danger sign you noticed during this lockdown? Can you […] your experience?<br>4. Why you were not able avail the antenatal services? (Probe: non-availability of transportation facilities, fear of visiting the hospital, anxiety to go out of house, family support, getting medicines, fear of getting COVID, spread of COVID to child, delivery, income).<br>Pregnant women	**INDIA**<br>Puducherry	2020<br>Jul-Jun	ANC	**PROVISION OF SERVICE**<br>• Respondents indicated that health facilities closed down their outpatient clinics during lockdown
Villalobos Dintrans (46)	2021	"…[t]o describe the perception of key actors regarding the interruption of health	**QUANTITATIVE**<br>Survey (survey questions provided)<br>Question:	**19 Latin American and Caribbean countries**	2020	General (includes MNH services)	**PROVISION OF SERVICE**<br>Services for newborns and pregnant women, such as institutional delivery care and

For peer review only - http://bmjopen.bmj.com/site/about/guidelines.xhtml

		services for populations not prioritized by the pandemic —pregnant women, newborns, children, adolescents, and women— in Latin American and Caribbean (LAC) countries during the first stage of the COVID-19 pandemic" (p. 01).	For the following health actions for pregnant women, newborns, children, adolescents and women, at a general level, indicate the state in your country during the month of June 2021. [have been maintained, have been partially reduced, have been suspended, Adapted to new circumstances, created new services, don't know].<br>Decision-makers and implementers of social and health programs and policies				postpartum care, as well as vaccination programs, showed the best perceptions of coverage during the pandemic.
Widiasih (34)	2021	"…to explore the experiences of pregnant women regarding ANC and monitoring fetal wellbeing during the COVID-19 pandemic" (p. 01).	**QUALITATIVE**<br>Semi-structured interview<br>(no interview guide provided)<br>Interviews described as:<br>"…covering the pregnant woman's experience when engaged in ANC and how and when they undertook fetal welfare checks during the COVID-19 pandemic".<br>Pregnant women	INDONESIA	2021<br>Jan	Self-foetal wellbeing monitoring and ANC	**PROVISION OF SERVICE**<br>• Respondents reported queues to access care, limits on the number of patients, and changes to service hours

For peer review only - http://bmjopen.bmj.com/site/about/guidelines.xhtml

BMJ Open

Measuring supply-side service disruption: A systematic review of the methods for measuring disruption in the context of maternal and newborn health services in low- and middle-income settings

Journal:	*BMJ Open*
Manuscript ID	bmjopen-2023-077583.R1
Article Type:	Original research
Date Submitted by the Author:	13-Nov-2023

Complete List of Authors:	McGowan, Catherine; London School of Hygiene & Tropical Medicine, Department of Infectious Disease Epidemiology<br>Gokulakrishnan, Dhikshitha; Leeds Teaching Hospitals NHS Trust<br>Monaghan, Emily; Save the Children International, Humanitarian Department; Croydon University Hospital<br>Abdelmagid, Nada ; London School of Hygiene & Tropical Medicine, Department of Infectious Disease Epidemiology<br>Romig, Laura; Save the Children Federation Inc, Department of Humanitarian Response<br>Gallagher, Meghan; Save the Children Federation Inc, Department of Humanitarian Response<br>Meyers, Janet; Save the Children Federation Inc, Department of Humanitarian Response<br>Cummings, Rachael; London School of Hygiene & Tropical Medicine, PHP; Save the Children International, Humanitarian Department<br>Cardinal, Laura; Save the Children International, Humanitarian Department

**Primary Subject Heading**:	Public health
Secondary Subject Heading:	Global health
Keywords:	COVID-19, Systematic Review, Quality in health care < HEALTH SERVICES ADMINISTRATION & MANAGEMENT, REPRODUCTIVE MEDICINE

SCHOLARONE™
Manuscripts

I, the Submitting Author has the right to grant and does grant on behalf of all authors of the Work (as defined in the below author licence), an exclusive licence and/or a non-exclusive licence for contributions from authors who are: i) UK Crown employees; ii) where BMJ has agreed a CC-BY licence shall apply, and/or iii) in accordance with the terms applicable for US Federal Government officers or employees acting as part of their official duties; on a worldwide, perpetual, irrevocable, royalty-free basis to BMJ Publishing Group Ltd ("BMJ") its licensees and where the relevant Journal is co-owned by BMJ to the co-owners of the Journal, to publish the Work in this journal and any other BMJ products and to exploit all rights, as set out in our licence.

The Submitting Author accepts and understands that any supply made under these terms is made by BMJ to the Submitting Author unless you are acting as an employee on behalf of your employer or a postgraduate student of an affiliated institution which is paying any applicable article publishing charge ("APC") for Open Access articles. Where the Submitting Author wishes to make the Work available on an Open Access basis (and intends to pay the relevant APC), the terms of reuse of such Open Access shall be governed by a Creative Commons licence – details of these licences and which Creative Commons licence will apply to this Work are set out in our licence referred to above.

Other than as permitted in any relevant BMJ Author's Self Archiving Policies, I confirm this Work has not been accepted for publication elsewhere, is not being considered for publication elsewhere and does not duplicate material already published. I confirm all authors consent to publication of this Work and authorise the granting of this licence.

For peer review only

Measuring supply-side service disruption: A systematic review of the methods for measuring disruption in the context of maternal and newborn health services in low- and middle-income settings

For peer review only

Catherine R. McGowan[1†], Dhikshitha Gokulakrishnan[2], Emily Monaghan[3,4], Nada Abdelmagid[1], Laura Romig[5], Meghan C. Gallagher[5], Janet Meyers[5], Rachael Cummings[3,6], Laura Cardinal[3]

1. Faculty of Epidemiology and Population Health, London School of Hygiene & Tropical Medicine, Keppel Street, London WC1E 7HT UK
2. Leeds Teaching Hospitals NHS Trust, Great George St, Leeds LS1 3EX
3. Humanitarian Department, Save the Children International, St Vincent House, 30 Orange Street, London, WC2H 7HH
4. Croyden University Hospital, 530 London Road, London CR7 7YE
5. Department of Humanitarian Response, Save the Children Federation Inc, 899 N. Capitol St #900, NE, Washington, DC 20002
6. Faculty of Public Health and Policy, London School of Hygiene & Tropical Medicine, 15-17 Tavistock Place, London WC1H 9SH

WORDS: 4970

† Corresponding author:

Catherine R. McGowan
Department of Infectious Disease Epidemiology
London School of Hygiene & Tropical Medicine
Keppel Street
London WC1E 7HT
United Kingdom
Catherine.McGowan@lshtm.ac.uk

ABSTRACT

Objectives: During the COVID-19 pandemic most essential services experienced some level of disruption. Disruption in LMICs was more severe than in HICs. Early reports suggested that services for maternal and newborn health were disproportionately affected, raising concerns about health equity. Most indicators measure demand-side disruption, or they conflate demand- and supply-side disruption. There is currently no published guidance on measuring supply-side disruption. The objective of this review was to identify methods and approaches used to measure supply-side service disruptions to maternal and newborn health services in the context of COVID-19.

Design: We carried out a systematic review and present a typology of measurement methods and approaches using narrative synthesis.

Data sources: We searched MEDLINE, EMBASE, and Global Health in January 2023. We also searched for grey literature.

Eligibility criteria: We included empirical studies describing the measurement of supply-side service disruption of maternal and newborn health services in LMICs in the context of COVID-19.

Data extraction and synthesis: We extracted the aim, method(s), setting, and study outcome(s) from included studies. We synthesised findings by type of measure (i.e. provision or quality of services) and methodological approach (i.e. qualitative or quantitative).

Results: We identified 28 studies describing five approaches to measuring supply-side disruption: 1) cross-sectional surveys of the nature and experience of supply-side disruption, 2) surveys to measure temporal changes in service provision or quality, 3) surveys to create composite disruption scores, 4) surveys of service users to measure receipt of services, and 5) clinic observation of the provision and quality of services.

Conclusion: Our review identified methods and approaches for measuring supply-side service disruption of maternal and newborn health services. These indicators provide important information about the causes and extent of supply-side disruption and provide a useful starting point for developing specific guidance on the measurement of service disruption in LMICs.

WORDS: 300

For peer review only

STRENGTHS AND LIMITATIONS OF THIS STUDY

- This study presents various methods and approaches used to measure supply-side disruption in the context of maternal and newborn health; despite their various limitations, these methods and approaches could be incorporated into preparedness and response guidance for measuring supply-side service disruption and for early mitigation against its effects on essential services.
- Despite the absence of comprehensive guidelines on measuring supply-side service disruption we were able to identify several studies describing methods and approaches used measure COVID-19 related disruption to maternal and newborn health services in low- and middle-income settings.
- This study presents a summary of qualitative findings about the experience of service disruption; these findings may be used to inform the design of quantitative indicators of supply-side service disruption.
- We did not carry out an assessment of study quality nor did we assess risk of bias; though this was appropriate given the aim to create a typology of quantitative indicators it may have introduced low quality and/or unreliable qualitative findings.

For peer review only

INTRODUCTION

With few exceptions, the COVID-19 pandemic exposed the vulnerabilities of health systems globally. In response to the burden of COVID-19 cases, many countries closed or scaled-back non-essential health services to allow relocation of resources to the COVID-19 response, and to reduce health system strain. In mid-2020 the WHO conducted the first of three key informant surveys amongst national health authorities in 105 countries (1). These 'pulse surveys' aimed to assess the impact of COVID-19 on 25 essential health services. Nearly all countries reported service disruptions, with lower-income countries reporting greater service disruption than higher income countries. The causes of disruption were identified as a mixture of service demand and supply factors. The most reported supply-side cause of disruption was the cancellation of elective services (reported by 66% of countries). Other supply-side causes included: staff redeployment to COVID-19 response (49%) or insufficient staff to provide services (29%), insufficient personal protective equipment for health care workers (44%), closure of health services (33%) or facilities (41%), and interruptions in the availability of medical supplies (30%) (1). Demand-side indicators may function as proxy indicators of supply insofar as they may evidence a reluctance to present to health services, or challenges with access. Demand-side indicators may demonstrate reduced uptake despite continuity, or scale up, of services.

Defining a package of essential services is a key preparedness activity; however, scaling back non-essential services may not be sufficient to avoid widespread and lasting disruption. The latest WHO pulse survey (published May 2023) notes that despite evidence of health system recovery, 84% of participating countries reported ongoing disruption to essential health services in the last quarter of 2022 (2), suggesting that disruption persists well into the recovery period. In June 2020 the WHO published operational guidance for maintaining essential health services during the pandemic (3). The guidance cautions that all health system adaptations "*…should be made in accordance with ethical principles, such as equity in the allocation of resources and access, self-determination, non-abandonment, and respect for dignity and human rights*" (3, p. 03). Though every country determines its own essential health services package based on its local contexts and capacities, the WHO recommended that seven health service categories be maintained during the acute phase of the pandemic including: 1) prevention and treatment of communicable diseases, 2) reproductive health services (including obstetric and child health), 3) core services for vulnerable populations (including infants and older adults), 4) chronic diseases (including mental health conditions), 5) critical facility-based therapies, 6) management of emergency health conditions, and 7) auxiliary services (including diagnostic imaging and laboratory services). The guidance emphasised that

For peer review only - http://bmjopen.bmj.com/site/about/guidelines.xhtml

critical decisions about the nature and timing of modifications to service delivery, "*… must be informed by the use of accurate and timely data throughout all phases of the COVID-19 pandemic*" (3, p. 18).

Data on service disruption should be suitable to inform rapid and appropriate adjustments (e.g. workforce optimisation strategies, resource allocation adaptations), including adjustments across essential services to ensure health equity (4). Whilst supply-side disruptions may contribute to decreased uptake (and vice versa), defining indicators which isolate the direction of disruption can inform measurements of the extent of demand-side disruption that can be attributed to supply-side disruption. In addition, monitoring essential health services requires meaningfully disaggregated data (to ensure that services are being delivered equitably), data that allow comparison of disruption over time (e.g. across key phases of the pandemic), and rapid needs assessments to evidence facility-level priorities (3, 5). Both the WHO guidance on maintaining essential health services, and the guidance on using routine data to monitor the effects of COVID-19 on essential services, include lists of sample indicators for measuring disruption; however, most of these indicators measure demand-side disruption as a proxy for supply-side disruption (e.g. number of women presenting to facility),or as a composite measure of both supply- and demand-side disruption (e.g. number of facility births) (4, 6).

Disruption of maternal and newborn health services during COVID-19

The negative impact of the COVID-19 pandemic on maternal and newborn health (MNH) outcomes has been well established at the population level. However, the causal pathways that create poor outcomes are less well understood (7). The evidence suggesting a direct link between maternal COVID-19 infection and maternal/newborn outcomes is limited (8, 9). It is suspected that disruption to MNH services – either in terms of reduced overall provision of services, or reduced quality of services – was primarily driving poor MNH outcomes (10). It has been estimated that supply and demand side disruption, combined with increasing food insecurity, could lead to between 12, 200 and 56, 700 excess maternal deaths globally (11). Vulnerable populations, including women and children, are experiencing the greatest effects (both direct and indirect) of the pandemic, thereby widening existing inequities (12) and reversing progress towards improving the health of these and other vulnerable groups (7, 10).

Though nearly all countries reported disruptions to essential health services, disruptions were often more severe in low- and middle-income (LMIC) countries (13). Whilst all essential health services have been disrupted by COVID-19, some have been disproportionately affected (13). Evidence suggests that MNH services have been disproportionately impacted by COVID-19-related service

For peer review only - http://bmjopen.bmj.com/site/about/guidelines.xhtml

disruption, particularly in LMIC settings (14, 15). A scoping review of the impact of COVID-19 on maternal, newborn, child health and nutrition concluded that a considerable amount of funding for these services in fragile and conflict-affected settings had been diverted to COVID-19 response activities (16). A recent systematic review aiming to synthesise the direct and indirect effects of the pandemic concluded that, "*…minor consideration was given to preserving and promoting health service access and utilization for mothers and children, especially in historically underserved areas in Africa*" (17, p. 1).

Rationale

The need for better service coverage data was identified early in the pandemic as the limitations of routinely collected data for reporting disruption became apparent (4). The literature currently overrepresents demand-side disruptions. Many crude health outcome indicators, such as number of stillbirths and maternal mortality, conflate supply-side and demand-side disruptions. Relatively few reports of COVID-19-related service disruption aim to isolate supply-side disruption yet measuring supply side disruptions is key to mitigating their effects. Whilst we were able to identify useful guidance on monitoring the effects of COVID-19 on essential health services, this guidance focussed exclusively on routinely collected data (6). Importantly, the authors caution that routine health information systems (RHIS), "…present an incomplete picture of the services used by communities because reporting rates are often low and many private care facilities (including those run by nongovernmental or religious organizations) do not report data to RHIS" (6, p. 5). In addition, RHIS present only a partial indication of the impact of COVID-19 on essential services in settings in which a significant proportion of health services are provided through the private sector (6). Finally, RHIS data vary in terms of data quality and completeness, and population denominators are particularly prone to error. This review aims to address existing gaps by describing how COVID-19-related, supply-side disruption has been measured in the context of MNH services in LMICs during COVID-19. In addition, the design of indicators is typically evidenced by qualitative research findings; as such, we sought to consolidate qualitative findings regarding the nature and extent of service disruption.

Objectives

The objectives of this review are: 1) to present methods and approaches used to *quantitatively* measure supply-side service disruptions in LMICs in the context of COVID-19 and MNH services, and 2) to consolidate the *qualitative* evidence of supply-side service and quality disruptions to MNH services in LMICs.

For peer review only - http://bmjopen.bmj.com/site/about/guidelines.xhtml

This review is reported against the PRISMA 2020 checklist (18) and updated reporting guidance (19). The review protocol has been published in the PROSPERO prospective systematic review registry (CRD42022381537) (20).

METHODS

Eligibility criteria

Eligibility was defined as any empirical source that reported on at least one indicator of supply-side disruption, regardless of method (i.e. quantitative or qualitative). We defined supply-side disruption broadly to include cancelling/scaling back of a MNH service, or reduction in quality (including reduction in staff numbers). We included studies reporting on any specific MNH clinical service (e.g. assisted delivery, antenatal care [ANC], postnatal care [PNC] for mothers and newborns, essential newborn care), MNH services more broadly (even if their components were not described), or studies of multiple essential services disaggregated by service and including at least one MNH service. We define supply-side disruption broadly to include disruption to the *provision* of care (e.g. mean difference in the number of health workers in the labour room), and disruption that compromises *quality* of care (e.g. provision of timely and appropriate care, standard precautions for preventing hospital-acquired infections are followed). We did not include in either category perceived disruptions to quality of care attributed to minimal and reasonable infection prevention and control requirements (e.g. the requirement to wear masks during consultation, social distancing whilst in a queue).

We did *not* include reports of disruption solely in the context of family planning or sexual health services; studies reporting only on MNH service uptake or indicators which combine or conflate demand side and supply side disruptions; or comparisons of baseline (e.g. pre-COVID) and follow-up outcome indicators (e.g. stillbirths) without isolating the effect of supply-side disruption. Sources were also excluded if they reported on supply chain disruption (e.g. blood for transfusion) without reporting on resulting discontinuation, scale-down, or reduced quality of services. We included sources written in any language. Finally, we excluded pre-prints.

Information sources and search strategy

We searched MEDLINE, EMBASE, and Global Health databases (via Ovid) for peer-reviewed and relevant grey literature on 23 January 2023; no start date was used as this was obviated by the inclusion of COVID-19 as a search term. We adapted our search strategy from the search methods used to maintain and update the Cochrane Pregnancy and Childbirth Group's Specialised Register (21). The complete database search strategy is included in **Appendix 1**. The grey literature searches

For peer review only - http://bmjopen.bmj.com/site/about/guidelines.xhtml

were carried out using Google Search (26 February 2023) and Duck Duck Go (23 February 2023) using independent search strings including: 'service disruption' and COVID; 'essential service disruption'; maternal and newborn and 'service disruption'; maternal and newborn and services and COVID. The team also searched relevant websites including the WHO Global Pulse Survey website (22) and the WHO COVID-19 Technical Guidance for Maintaining Essential Services and Systems website (23).

Selection process, data collection and synthesis

The lead author [CM] carried out the initial screening of all sources retrieved via database searching. Screening of grey literature sources was carried out by two authors [EM/LR]. The lead author manually extracted all data in NVivo 1.0 (Melbourne, AU: QSR International Pty Ltd) based on the following characteristics and domains: title, year, author, aim, method, setting, time (data collection), programme or population, findings, and definition of disruption. We compiled a narrative synthesis of the literature. Only data on supply-side disruption were extracted from sources reporting on both demand-side and supply-side factors. We define *quality of service* in line with WHO definition of quality (i.e. the service is effective, safe, people-centred, timely, equitable, integrated, and efficient) and the WHO standards for improving the quality of maternal and newborn care (24, 25). We define *provision of service* to include cessation or scale back of services.

Reporting bias and certainty assessment

We did not assess bias of the studies themselves. Whilst we had intended to use an established guideline to evaluate the quantitative indicators identified in the literature, no source provided sufficient information to allow a complete quality assessment.

Patient and public involvement

None.

RESULTS

Study selection

The initial database search returned 4,438 records published on or before 23 January 2023. We used EndNote 20.5 (Philadelphia, PA: Clarivate) to delete duplicates which resulted in 2657 unique records. Initial screening was carried out based on review of the title/abstract and resulted in 94 sources for which we were able to retrieve 85 full-text documents. 28 peer-reviewed sources and three grey literature sources (reported below as one source) remained following full-text review. The PRISMA Flow chart is included in **Figure 1**.

For peer review only - http://bmjopen.bmj.com/site/about/guidelines.xhtml

INSERT FIGURE 1 HERE

The reasons for exclusion, following full-text review, were: only reported on uptake (n=18), not sufficiently focussed on disruption (n=18), not sufficiently focussed on MNH (n=5), not sufficiently focussed on LMICs (n=2), review article (n=3), not empirical (n=2), no disruption (n=3), pre-print (n=6), and evaluation of a particular MNH intervention (n=1). The grey literature search produced 43 unique results of which 10 were included in the final synthesis. None of the grey literature sources met the inclusion criteria as: they were not empirical (n=9), were not focussed on MNH (n=2), presented only demand-side indicators (n=6), or they included indicators of disruption which did not isolate supply-side factors (n=4).

Study characteristics

The characteristics of the 28 included studies are listed in **Appendix 2**. Studies were carried out in: Bangladesh (26), Ethiopia (27, 28), India (29-34), Indonesia (35, 36), Iran (37, 38), Nepal (39, 40), Nigeria (41-44), and Uganda (45, 46). Three studies included multiple LMIC countries (14, 47, 48), and four included both HIC and LMIC countries (49-52). Most studies (n=18) used qualitative methods to collect data about supply-side disruption (28, 30, 31, 33-37, 40-47, 49, 50); one study involved a qualitative media analysis (32). Seven studies were based on quantitative methods (14, 26, 27, 29, 38, 48, 52), and two used both qualitative and quantitative methods (39, 51). Most studies (n= 14) involved participation from service-providers (e.g. nurses, midwives, obstetricians, community health workers, policy-makers) (14, 29, 30, 35, 40, 42-44, 47-52), whilst 11 involved service-users (27, 31-34, 36-39, 45, 46), and three included both groups (26, 28, 41).

Results of synthesis

We report below a synthesis of the quantitative approaches used to measure supply-side disruptions to the provision of services and/or the quality of services due to COVID-19 – a summary of these findings can be found in **Tables 1 and 2**. In addition, we summarise the qualitative approaches to measuring supply-side disruption.

INSERT TABLE 1 HERE

Quantitative measures of disruption

Quantitative indicator data were collected using the following approaches:

Surveys of health care workers or health stakeholders about disruptions (51, 52) or missed services (27) were carried out in four studies. The first involved distributing a survey amongst health care workers in 51 countries (including 32 LMICs) – asking if they were aware of any problems providing MNH immunisation services (yes/no) – if respondents answered yes, they were asked to indicate the

For peer review only - http://bmjopen.bmj.com/site/about/guidelines.xhtml

nature of the problems (described below in the section on qualitative measures) (51). This approach produced a crude proportionate measure of subjective assessment of disruption to vaccine delivery across several countries (e.g. 53% of LMIC-based respondents reported disruptions to maternal immunisation services compared to 42% of HIC-based respondents).

Using a pre-defined list of disruptions to the provision and quality of services, a study in 61 countries (including 34 LMICs) asked health care workers to indicate how specific MNH services (e.g. inpatient/outpatient post-natal care) were affected by COVID-19 in the preceding month (52). This study produced proportionate measures of the subjective assessment of the causes of disruption to post-natal care across multiple countries; for example, respondents from LMICs reported various disruptions due to COVID-19 including reduced numbers of beds due to social distancing [37%], cessation of home visits [20%], and suspension of post-natal care [8%] (52).

A study amongst health stakeholders in 19 Latin American and Caribbean countries asked respondents to indicate their perception of the disruption to various health services (including MNH) during the pandemic using a Likert scale (i.e. services have been maintained, partially reduced, suspended, adapted to COVID-19, and new services were created) (48). The study concluded that services for newborns and pregnant women (e.g. institutional delivery care, postpartum care, vaccination programs) were found to have showed the best perceptions of coverage during the pandemic (48).

Finally, a survey to assess disruption to ANC services was sent to pregnant women in Ethiopia asking, '*Did you miss or were late to start an ANC visit during the COVID-19 outbreak?'* (yes/no) (27). Respondents were asked to indicate a reason for missed or delayed ANC visits from a selection of responses describing facility-related factors for disruption; such as, "deploying of maternal care workers", "interruption and diversion of maternity services to the COVID-19 response", "fear of COVID-19 infection", and "social distancing". The study produced a measure of the proportion of respondents who experienced a disrupted ANC visit (which does not isolate supply-side factors) as well as a proportionate subjective assessment of the causes of the disruption. Two-hundred and sixteen (out of 389) respondents reported missed or late ANC visits, of which 72 [33.33%] attributed this to diversion of maternal services to COVID-19 (27).

INSERT TABLE 2 HERE

Surveys of health care workers or health stakeholders (to measure temporal changes in disruption) were used in two ways: 1) in India retrospective baseline data, in addition to accounts of the situation at the time of the interview were compared (29), and 2) in Bangladesh, data about service provision and service quality were collected from health care workers and mothers with children

For peer review only - http://bmjopen.bmj.com/site/about/guidelines.xhtml

under two at baseline (i.e. time 1 [T1]), and at T3 (which includes retrospective questions about T2) (26).

The study from India involved sending a survey to health care workers asking them if they were able to provide ANC services to all pregnant women during the most recent lockdown (T1), and in the preceding month (T2). Responses were: 0 (none), 1 (yes, but only to some pregnant women), or 2 (yes, to all pregnant women). Data about pre-pandemic service provision (T0) were collected from hospital HIS systems (29); however, though these data were chosen to provide a baseline measure that aligned with the questions in the survey, the health information system (HIS) indicators (i.e. # of women receiving >4 ANC check-ups, and the number of women given iron and folic acid tablets) conflate demand-side and supply-side factors. The study provided the proportion of women provided ANC services at T1 and T2 (as a measure of changes in service provision), and the number of women receiving services at T0. The study found that ANC services were disrupted during the lockdown period but were restored during the post-lockdown period, but not to pre-pandemic levels) (29).

The study from Bangladesh included a baseline assessment carried out in early 2020 asking health care workers about their exposure to training, their workload and time commitments, and the types of services, specifically health and nutrition services for mothers and young children, they were providing (e.g. was service X provided in T1) (26). Data were also collected from pregnant women and mothers of young children (e.g. was service X received) at the same time points. The study provides a proportionate measure of service provision (defined as the proportion of service-providers indicating provision of specific services, and the proportion of service-users indicating receipt of specific services) at pre-pandemic (T0), lockdown (T1), and post lockdown (T2). The study found that fewer facilities offered ANC services during lockdown when compared to before the pandemic (dropping 6.6 percentage points), and that there were significant drops in the receipt of anthropometric measurements, and iron/folic acid/calcium supplementation. All services recovered post-lockdown but not to pre-pandemic levels (26).

Surveys of health care workers to construct composite service disruption scores for specific services (including MNH services) were used in Burkina Faso, Ethiopia, and Nigeria (14). Responses were scored 0 (no interruption), 1 (partial interruption), 2 (complete interruption) for each of the three MNH services (i.e. ANC, iron and folate supplementation, and family planning services) for a maximum disruption score of six. The average of all individual responses was calculated for each of the three study settings. Maternal and reproductive services were scored an average of 2.24 (out of six) for Nigeria, 1.72 for Burkina Faso, and 1.67 for Ethiopia (14).

For peer review only - http://bmjopen.bmj.com/site/about/guidelines.xhtml

Survey of service users to measure receipt of health services was carried out in Iran to assess the Actual Percentage of Service Delivery (APSD) (38). An SMS message - i.e. *'Has the service been received?'* (yes/no) - was sent to all patients attending hospital services. Data were collected before and during the pandemic and were aggregated by calendar month. The study indicates the proportion of attendees who received a service, by month (e.g. the study found that there was a small but significant drop in the ASPD for midwifery services when comparing the pandemic to pre-pandemic periods) (38).

Observation of health facilities was carried out in Nepal, using a structured clinical observation checklist, to determine the number of health care workers in the labour and delivery room, the number of workers redeployed to COVID-19, and the number of days of disruption of maternity services (i.e. no maternity services provided on these days) (39). The study collected baseline data over six months in 2019 (pre-pandemic period), followed by data collection over six months in 2020 (pandemic period). The study measured the mean difference in the number of health workers in the labour room and the number of days that maternity services were disrupted between the two time periods. The study provides a mean number of days (4.3) that maternity services were halted during the pandemic period (March – August 2019), and concluded that the number of health care workers (per 24 hours in the labour and delivery room) had decreased to 5.4 (from 6.2 before the pandemic) due to redeployment to COVID-19-dedicated care (39).

Qualitative measures of disruption

Various qualitative approaches were used to generate accounts of disruptions to the provision or quality of services including: 1) semi-structured interviews with service providers and/or service users to elicit descriptions of experiences of disruption (28, 30, 31, 33, 34, 36, 37, 39-46), 2) semi-structured interviews with service providers eliciting comparisons of the provision of care at different times (35, 49), 3) semi-structured interviews amongst service users asking about management, barriers, and facilitators influencing service delivery (stratified by levels of health system) (47), 4) free-text field in a survey of service providers (50, 51) and 5) media analysis of news reports (32). Some of these sources focussed on the experiences of respondents during a lockdown period (34), or during a lockdown period compared to the period preceding or following lockdown (33, 42). Only six of these studies published the original data collection instrument (31, 34, 41, 43, 47, 51).

Provision of services

Disruptions to provision of services included: closed health facilities or village health posts (30, 39), facilities that had closed certain wards/units/clinics/services (e.g. outpatient clinics (34, 44, 50),

For peer review only - http://bmjopen.bmj.com/site/about/guidelines.xhtml

doctor's offices (37), support for kangaroo mother care (40), counselling for care of mother and newborn (40)), limits to the number of patients that could be seen (36, 46), lack of follow-up (46, 50) or home visits (35, 43), redeployment of staff to support COVID-19 services (32, 39, 43, 50), reduced beds or space (43), reduced hours of service (46, 47), health facilities converted to COVID-19 care centres (33, 47), unavailable health care workers (33, 47), lack of laboratory services (30), and specific services (e.g. intra-natal services) unavailable (31) or reduced (31, 35, 42, 43, 47).

Quality of services

Disruptions to the quality of services included: poor quality of care (32, 33, 37, 40, 46, 47, 49), long waiting times (33, 36, 45), poor attitude of health care workers towards clients (28), reduced numbers of health care workers (39, 40, 44, 47), reduced physical and/or emotional support (49), staff preoccupied with infection prevention and control (46), unhygienic conditions (47), shorter times with health care workers (37, 40, 47), health care workers unwilling to perform certain examinations/procedures (e.g. abdominal palpation, auscultation of foetal heart rate) (37, 47), increased risk of medically unjustified caesarean section (49), and shortages of resources (including medicines, vaccines and/or oxygen) were also reported (28, 37, 47, 51).

DISCUSSION

Our initial screening (on title and abstract) revealed that the majority of the literature on service disruption reports on demand-side measures. This was also a finding of a recent review of health system performance indicators (53). Our search identified a small number of measures isolating supply-side factors (N=28). Amongst these, seven sources included quantitative measures, of which two focussed on the causes (as opposed to the presence or extent) of disruption (27, 52). The 19 qualitative (and two mixed methods) studies included rich descriptions of the experiences of supply-side delay from the perspective of both service-providers and service-users, yet none of the findings from these studies appear to have informed the design of quantitative surveys. Only one study used empirical findings (from a previous iteration of the study survey) to pre-define survey fields describing the nature of service disruption (52). However, we believe these qualitative studies may provide important contextual relevance and granularity to future surveys.

Emergency preparedness guidance documents often emphasise the importance of monitoring and evaluation but typically do not include indicators of health system disruption. The WHO's COVID-19 Strategic Preparedness and Response Plan states, "*…it is important to track health system capacity and performance; including hospital and ICU bed occupancy rates, as well as the health system's ability to continue providing non-COVID-19 services*"; however, a bank of specific indicators is not

For peer review only - http://bmjopen.bmj.com/site/about/guidelines.xhtml

included (54). The evidence we identified suggests that approaches to measuring supply-side disruption are varied, inconsistent, and largely *ad hoc*. We identified a small number of measures of supply-side disruption that included real time baseline measurements; this suggests that systems ought to be in place at the onset of a pandemic to allow for the collection of baseline measurements. Retrospective baseline measurements may lack precision as they are typically prone to recall and confirmation biases. In addition, early planning for periodic follow-up is important as, "…metrics of system responses and resilience may only be understood appropriately as we relate them to the severity and dynamics of the shock itself" (55, p. 1202).

Facility assessments (and community assessments) designed to establish the continuity of essential services in the context of COVID-19 are important tools for monitoring the provision and quality of care; however, they may require adaptation for future outbreaks (and other crises), and may not be suitable for specific health programmes or priorities (5, 56). In addition, our review suggests that there are additional approaches to measuring supply-side disruption that may provide an important complement to facility assessments. The evidence also suggests that qualitative interviews may be a more suitable approach to collecting data about quality of services and may provide the opportunity for participants to offer localised solutions. Qualitative methods may also clarify the mechanisms through which services are affected (e.g. including cascading disruptions, proxy measures of disruption) which may help to identify targeted mitigation strategies (53).

Finally, we excluded one source reporting on a clinical review of patient records to assess the extent of unnecessary hospitalisations of pregnant women in Tajikistan (57). This study was excluded as it was focussed on demand side indicators (i.e. total hospitalisations before and during COVID-19) and only assessed unnecessary hospitalisations for two months during COVID-19 as a measure of the strength of the existing primary health care system. However, though the indicator was not suitable for assessing COVID-19 related disruption in this context, we believe that unnecessary hospital admissions (and unnecessarily prolonged admissions) could be a suitable indicator of disruption to quality of primary health care - insofar as it reflects poor organisation, poor use of resources, and reduces inpatient capacity - if collected prospectively or retrospectively to include the pre-outbreak period. Ultimately, this example highlights that service uptake may function as a proxy indicator of quality; however, service uptake alone is not suitable as a robust measure of the quality of care provided or received.

Limitations of the evidence

Many of the included sources provide weak description of methods (only six of the 28 sources include the original survey or the topic guide). Some sources aim to measure supply-side disruption

For peer review only

For peer review only - http://bmjopen.bmj.com/site/about/guidelines.xhtml

but present crude measures of provision (e.g. number of ANC visits provided per day) which likely conflate supply and demand-side disruption. Whilst the qualitative studies provide important context-specific information, they may not be transferable to other settings. Many respondents indicated that services were closed or that they were of low quality during the pandemic (or specifically during lockdown) but few sources aimed to determine to what extent services were available, and at what comparative quality, prior to the pandemic. Concerns are often raised about the pandemic undermining the steady gains made in the provision of MNH services in recent years (10); thus, comparing disruption to a baseline measure fails to account for gains that would have been made otherwise and may therefore underestimate the true extent of the disruption.

Limitations of the review

Whilst COVID-19 related disruption was globally pervasive, there are relatively few sources focussed on the disruption of MNH services. As service disruption may have been assumed to be unavoidable (and thus not worthy of measuring), it may be underreported. Similarly, acknowledging disruption may be viewed as a liability, or may be considered demoralising to service-providers, which may have resulted in a reluctance to measure disruption or publish findings. We opted for a broad search strategy to avoid missing sources that did not specifically reference 'disruption'; however, we still may have missed relevant sources which did not reference 'service' or 'programme' in the title or abstract. In addition, we suspect that publication delay may have limited the amount of available evidence. Finally, we were unable to assess the quality of the evidence due to gaps in reporting across nearly all the identified studies; as such, we may have included studies that would have otherwise been excluded.

CONCLUSION

The evidence suggests: an overrepresentation of demand-side indicators, varied and inconsistent methods for isolating supply-side disruption, few published studies of supply-side disruption that include baseline data (with most relying on retrospective baseline estimates or subjective estimation of change over time), relatively few of the quantitative indicators are designed to measure the causes of disruption, studies reporting on supply-side disruption from the perspective of service-users, and a lack of a clear or nuanced (i.e. including both the provision of services, and quality of services) framework for defining supply-side disruption. There are few studies which measure disruption across all essential services. Whilst in some settings MNH services were reported to be less disrupted than other essential services, it is still important to measure disruption across all essential services to ensure equitable scale-down in response to supply-side constraints.

For peer review only - http://bmjopen.bmj.com/site/about/guidelines.xhtml
For peer review only

Taken together, the evidence emphasises the importance of monitoring essential health services and highlights the need for specific guidance for defining service disruption and for developing indicators that are able to isolate supply-side disruption both of the provision and quality of services. Given the difficulty of setting up robust monitoring systems during an emergency, and the value both of baseline measures and regular follow-up to identify the early signs of disruption, we recommend that strategies for measuring disruption be developed and incorporated into preparedness plans. Finally, we suggest that preparedness plans provide specific indicators – including indicators for measing disruption to MNH services – alongside guidance on how data should be collected, and the resources required to support data collection and reporting in an emergency.

OTHER

This review was made possible by the generous support of the American people through the United States Agency for International Development (USAID). The contents are the responsibility of READY Initiative and do not necessarily reflect the views of USAID or the United States Government.

The review protocol has been published in the PROSPERO prospective systematic review registry (CRD42022381537).

CONTRIBUTOR STATEMENT

Conceptualisation: CM/EM/LC Formal analysis: CM/NA/LR/MG/JM Funding acquisition: LC Methodology: CM/DG Project administration: LR/JM/LC Supervision: EM/JM/RC/LC Writing (original draft): CM Writing (reviewing and editing): CM/DG/EM/NA/LR/MG/JM/RC/LC.

COMPETING INTERESTS

There are no competing interests for any author.

FUNDING

This review was made possible by the generous support of the American people through the US Agency for International Development (USAID).

DATA SHARING STATEMENT

All data relevant to the study are included in the article or uploaded as supplementary information.

For peer review only - http://bmjopen.bmj.com/site/about/guidelines.xhtml

ETHICS APPROVAL

This project did not require review by a research ethics committee.

For peer review only

For peer review only - http://bmjopen.bmj.com/site/about/guidelines.xhtml

REFERENCES

1. World Health Organization. Pulse survey on continuity of essential health services during the COVID-19 pandemic [Interim report]. Geneva: WHO; 2020.

2. World Health Organization. Fourth round of the global pulse survey on continuity of essential health services during the COVID-19 pandemic: November 2022–January 2023: interim report, 1 May 2023. Geneva: WHO; 2023.

3. World Health Organization. Maintaining essential health services: operational guidance for the COVID-19 context: interim guidance. Geneva: WHO; 2020.

4. World Health Organization. Maintaining the provision and use of services for maternal, newborn, child and adolescent health and older people during the COVID-19 pandemic: Lessons learned from 19 countries. Geneva: WHO; 2021.

5. World Health Organization. Continuity of essential health services: Facility assessment tool. Geneva: WHO; 2021.

6. World Health Organization. Analysing and using routine data to monitor the effects of COVID-19 on essential health services: Practical guide for national and subnational decision-makers. Geneva: WHO; 2021.

7. Chmielewska B, Barratt I, Townsend R, Kalafat E, van der Meulen J, Gurol-Urganci I, et al. Effects of the COVID-19 pandemic on maternal and perinatal outcomes: a systematic review and meta-analysis. Lancet Glob Health. 2021;9(6).

8. Di Toro F, Gjoka M, Di Lorenzo G, De Santo D, De Seta F, Maso G, et al. Impact of COVID-19 on maternal and neonatal outcomes: a systematic review and meta-analysis. Clin Microbiol Infect. 2021;27(1).

9. Kotlar B, Gerson E, Petrillo S, Langer A, Tiemeier H. The impact of the COVID-19 pandemic on maternal and perinatal health: a scoping review. Reprod Health. 2021;18(1).

10. Srivastava K. Covid-19: Why has India had a spike in stillbirths? BMJ. 2021;374.

11. Roberton T, Carter ED, Chou VB, Stegmuller AR, Jackson BD, Tam Y, et al. Early estimates of the indirect effects of the COVID-19 pandemic on maternal and child mortality in low-income and middle-income countries: a modelling study. Lancet Glob Health. 2020;8(7).

12. Gurley N, Ebeling E, Bennett A, Kashondo JK, Ogawa VA, Couteau C, et al. National policy responses to maintain essential health services during the COVID-19 pandemic. Bull World Health Organ. 2022;100(2).

13. World Health Organization. Pulse survey on continuity of essential health services during the COVID-19 pandemic: interim report, 27 August 2020. Geneva: WHO; 2020.

For peer review only - http://bmjopen.bmj.com/site/about/guidelines.xhtml

14. Assefa N, Sie A, Wang D, Korte ML, Hemler EC, Abdullahi YY, et al. Reported Barriers to Healthcare Access and Service Disruptions Caused by COVID-19 in Burkina Faso, Ethiopia, and Nigeria: A Telephone Survey. Am J Trop Med Hyg. 2021;105(2).

15. United Nations Children's Fund. Futures at risk: The impact of COVID-19 on maternal, newborn, and child health in fragile and conflict-affected settings. New York, NY: UNICEF; 2021.

16. Rodo M, Singh L, Russell N, Singh NS. A mixed methods study to assess the impact of COVID-19 on maternal, newborn, child health and nutrition in fragile and conflict-affected settings. Confl Health. 2022;16(1).

17. Adu PA, Stallwood L, Adebola SO, Abah T, Okpani AI. The direct and indirect impact of COVID-19 pandemic on maternal and child health services in Africa: a scoping review. Glob Health Res Policy. 2022;7(1).

18. Page MJ, McKenzie JE, Bossuyt PM, Boutron I, Hoffmann TC, Mulrow CD, et al. The PRISMA 2020 statement: an updated guideline for reporting systematic reviews. BMJ (Clinical research ed). 2021;372.

19. Page MJ, Moher D, Bossuyt PM, Boutron I, Hoffmann TC, Mulrow CD, et al. PRISMA 2020 explanation and elaboration: updated guidance and exemplars for reporting systematic reviews. BMJ. 2021;372.

20. National Institute for Health Research. READY maternal and new-born health service disruption associated with COVID-19: a systematic review York: PROSPERO; 2022 [Available from: https://www.crd.york.ac.uk/prospero/display_record.php?RecordID=381537https://www.crd.york.ac.uk/prospero/display_record.php?RecordID=381537].

21. Alfirevic Z KE, Dowswell T, et al. Which method is best for the induction of labour? A systematic review, network meta-analysis and cost-effectiveness analysis. (Health Technology Assessment, No. 20.65.) Appendix 2, Search strategy Southampton (UK): NIHR Journals Library (Cochrane Pregnancy and Childbirth Group); 2016 [Available from: https://www.ncbi.nlm.nih.gov/books/NBK379828/.

22. World Health Organization. Global pulse survey on continuity of essential health services during the COVID-19 pandemic Geneva: WHO; 2023 [Available from: https://www.who.int/teams/integrated-health-services/monitoring-health-services/global-pulse-survey-on-continuity-of-essential-health-services-during-the-covid-19-pandemic.

23. World Health Organization. Coronavirus disease (COVID-19) technical guidance: Maintaining Essential Health Services and Systems Geneva: WHO; 2023 [Available from: https://www.who.int/emergencies/diseases/novel-coronavirus-2019/technical-guidance/maintaining-essential-health-services-and-systems.

24. World Health Organization. Quality health services. Geneva: WHO; 2020.

25. World Health Organization. Standards for improving quality of maternal and newborn care in health facilities. Geneva: WHO; 2016.

For peer review only - http://bmjopen.bmj.com/site/about/guidelines.xhtml

26. Nguyen PH, Sununtnasuk C, Pant A, Tran Mai L, Kachwaha S, Ash D, et al. Provision and utilisation of health and nutrition services during COVID-19 pandemic in urban Bangladesh. Matern Child Nutr. 2021;17(4).

27. Tadesse E. Antenatal Care Service Utilization of Pregnant Women Attending Antenatal Care in Public Hospitals During the COVID-19 Pandemic Period. Int J Women Health. 2020;12.

28. Tilahun B, Nigusie A, Zelalem M, Mekonnen ZA. Effect of COVID-19 Pandemic on Maternal and Child Health Services and Strategies for Effective Service Implementation in Ethiopia. J Multidiscip Healthc. 2022;15.

29. Avula R, Nguyen PH, Ashok S, Bajaj S, Kachwaha S, Pant A, et al. Disruptions, restorations and adaptations to health and nutrition service delivery in multiple states across India over the course of the COVID-19 pandemic in 2020: An observational study. PLoS ONE. 2022;17(7).

30. Padhye R, Purushotham A, Paul M, Sardeshpande N, Ballala R, Dhar S, et al. Accessing Maternal Health Care in the Midst of the COVID-19 Pandemic: A Study in Two Districts of Assam, India. Front. 2022;3.

31. Sahoo KC, Doley C, Negi S, Das S, Verma P, Kanungo S, Pati S. Experiences of Urban Slum-Dwelling Women With Maternal and Child Health Services During COVID-19 Pandemic: A Multi-City Qualitative Study From India. Int J Public Health. 2022;67.

32. Shrivastava S, Rai S, Sivakami M. Challenges for pregnant women seeking institutional care during the COVID-19 lockdown in India: A content analysis of online news reports. Indian J Med Ethics. 2021;VI(3).

33. Sinha B, Dudeja N, Mazumder S, Kumar T, Adhikary P, Roy N, et al. Estimating the Impact of COVID-19 Pandemic Related Lockdown on Utilization of Maternal and Perinatal Health Services in an Urban Neighborhood in Delhi, India. Front. 2022;3.

34. Ulaganeethi R, Dorairajan G, Ramaswamy G, Thekkur P, Olickal JJ, Rajkumari N, Kumar Saya G. 'I was scared I will end up in another abortion': a mixed-methods study assessing the impact of COVID-19 pandemic and lockdown on the antenatal care of pregnant women in Puducherry, South India. Fam Pract. 2021;38(Suppl 1).

35. Hazfiarini A, Zahroh RI, Akter S, Homer CSE, Bohren MA. Indonesian midwives' perspectives on changes in the provision of maternity care during the COVID-19 pandemic: A qualitative study. Midwifery. 2022;108.

36. Widiasih R, Hidayat D, Zakaria H, Utama DQ, Komariah M, Maryam NNA, et al. Self-Fetal Wellbeing Monitoring and Ante-Natal Care during the COVID-19 Pandemic: A Qualitative Descriptive Study among Pregnant Women in Indonesia. Int J Environ Res Public Health. 2021;18(21).

37. Mirzakhani K, Shoorab NJ, Akbari A, Khadivzadeh T. High-risk pregnant women's experiences of the receiving prenatal care in COVID-19 pandemic: a qualitative study. BMC Pregnancy Childbirth. 2022;22(1).

For peer review only - http://bmjopen.bmj.com/site/about/guidelines.xhtml

38. Rezapour R, Dorosti AA, Farahbakhsh M, Azami-Aghdash S, Iranzad I. The impact of the Covid-19 pandemic on primary health care utilization: an experience from Iran. BMC Health Serv Res. 2022;22(1).

39. Ashish KC, Peterson SS, Gurung R, Skalkidou A, Gautam J, Malla H, et al. The perfect storm: Disruptions to institutional delivery care arising from the COVID-19 pandemic in Nepal. J. 2021;11.

40. Basnet B, Chapagain P, Subedi S, Dahal T, Neupane S, Khanal R, et al. Experiences of nurses providing maternity care in a public hospital during the COVID-19 pandemic in Nepal: a qualitative study. PLoS Global Public Health. 2022;2(4).

41. Akaba GO, Dirisu O, Okunade KS, Adams E, Ohioghame J, Obikeze OO, et al. Barriers and facilitators of access to maternal, newborn and child health services during the first wave of COVID-19 pandemic in Nigeria: findings from a qualitative study. BMC Health Serv Res. 2022;22(1).

42. Adelekan B, Goldson E, Abubakar Z, Mueller U, Alayande A, Ojogun T, et al. Effect of COVID-19 pandemic on provision of sexual and reproductive health services in primary health facilities in Nigeria: a cross-sectional study. Reprod Health. 2021;18(1).

43. Leung C, Olufunlayo T, Olateju Z, MacArthur C, Taylor B. Perceptions and experiences of maternity care workers during COVID-19 pandemic in Lagos State, Nigeria; a qualitative study. BMC Health Serv Res. 2022;22(1).

44. Mohammed BA, Abubakar D, Muhammad SA, Aremu Y, Onazi SO, Akeredolu F, et al. Impact of COVID-19 on maternal newborn child health services in Federal Medical Centre Gusau, Zamfara State. Science World Journal. 2022;17(1).

45. Musiimenta A, Tumuhimbise W, Atukunda EC, Ayebaza S, Kobutungi P, Mugaba AT, et al. Challenges in accessing maternal and child health services during COVID-19 and the potential role of social networking technologies. Digit Health. 2022;8.

46. Nakate MG, Mackay S, Ndirangu-Mugo E, Fleming V. Experiences of mothers and significant others in accessing comprehensive healthcare in the first 1000 days of life post-conception during COVID-19 in rural Uganda. BMC Pregnancy Childbirth. 2022;22(1).

47. PSBI Formative Research Study Group. Barriers to optimal care and strategies to promote safe and optimal management of sick young infants during the COVID-19 pandemic: A multi-country formative research study. J. 2022;12.

48. Villalobos Dintrans P, Maddaleno M, Granizo Roman Y, Valenzuela Delpiano P, Castro A, Vance C, C AC. [Disruption of health services for pregnant women, newborns, children, adolescents, and women during the COVID-19 pandemic: ISLAC 2020 ProjectInterrupcao dos servicos de saude para gravidas, recem-nascidos, criancas, adolescentes e mulheres durante a pandemia de COVID-19: projeto ISLAC 2020]. Rev Panam Salud Publica. 2021;45.

49. Asefa A, Semaan A, Delvaux T, Huysmans E, Galle A, Sacks E, et al. The impact of COVID-19 on the provision of respectful maternity care: Findings from a global survey of health workers. Women Birth. 2022;35(4).

For peer review only - http://bmjopen.bmj.com/site/about/guidelines.xhtml

50. Rao SPN, Minckas N, Medvedev MM, Gathara D, Y NP, Seifu Estifanos A, et al. Small and sick newborn care during the COVID-19 pandemic: global survey and thematic analysis of healthcare providers' voices and experiences. BMJ Global Health. 2021;6(3).

51. Saso A, Skirrow H, Kampmann B. Impact of COVID-19 on Immunization Services for Maternal and Infant Vaccines: Results of a Survey Conducted by Imprint-The Immunising Pregnant Women and Infants Network. 2020;8(3).

52. Semaan A, Dey T, Kikula A, Asefa A, Delvaux T, Langlois EV, et al. "Separated during the first hours" - postnatal care for women and newborns during the COVID-19 pandemic: a mixed-methods cross-sectional study from a global online survey of maternal and newborn healthcare providers. PLoS Global Public Health. 2022;2(4).

53. Turcotte-Tremblay A-M, Leerapan B, Akweongo P, Amponsah F, Aryal A, Asai D, et al. Tracking health system performance in times of crisis using routine health data: lessons learned from a multicountry consortium. Health Research Policy and Systems. 2023;21(1).

54. World Health Organization. COVID-19 Strategic Preparedness and Response Plan Operational Planning Guideline. Geneva: WHO; 2021.

55. Fleming P, O'Donoghue C, Almirall-Sanchez A, Mockler D, Keegan C, Cylus J, et al. Metrics and indicators used to assess health system resilience in response to shocks to health systems in high income countries-A systematic review. Health Policy. 2022;126(12).

56. World Health Organization. Community needs, perceptions and demand: community assessment tool: A module from the suite of health service capacity assessments in the context of the COVID-19 pandemic. Geneva: WHO; 2021.

57. World Health Organization. Data availability for assessing disruption of essential services for maternal, newborn, child and adolescent health during COVID-19: lessons learned from Tajikistan. Geneva: WHO; 2022.

For peer review only

FIGURE LEGEND

Figure 1: PRISMA flow chart

FIGURE 1: PRISMA flow chart

ACKNOWLEDGEMENTS

We would like to thank Dr Louisa Baxter for providing guidance on clinical and programming quality. Thank you to Dr Alicia Renedo for coding the Spanish language source. Thank you also to Dr Shirley Huchcroft for her invaluable feedback on an early draft of this paper.

For peer review only

For peer review only - http://bmjopen.bmj.com/site/about/guidelines.xhtml

TABLE 1: MEASURES OF DISRUPTION

	PROVISION OF SERVICES	QUALITY OF SERVICES
QUALITATIVE METHODS	• Semi-structured interviews with health care workers and/or service users asking about difficulties providing care (28, 30, 31, 33, 34, 36, 37, 39, 40, 42-46)<br>• Free-text field in a survey (50, 51)<br>• Semi-structured interviews with health care workers asking how they provided care at T1 (retrospective baseline) and how they currently (T2) provide care (35)<br>• Semi-structured interviews with health care workers which ask about impacts at various levels of the health system (47)<br>• Review of news articles reporting on disruption (32)	• Semi-structured interviews with health staff and/or service users (28, 30, 33, 37, 39-41, 43, 44, 46, 50, 51)<br>• Surveys of health workers which include a free-text field asking to what extent has care been provided at T1 (retrospective baseline) compared to T2 (49)<br>• Semi-structured interviews with health care workers which ask about impacts at various levels of the health system (47)<br>• Review of news articles reporting on disruption (32)

For peer review only - http://bmjopen.bmj.com/site/about/guidelines.xhtml

**QUANTITATIVE METHODS**	• Observation of health facilities (to determine number of health care workers on wards, days service disrupted) (39)<br>• Surveys of health care workers which ask respondents to provide a 'disruption score' (14)<br>• Surveys of health care workers which ask about service provision at T1 (retrospective baseline) and T2 (29)<br>• Surveys of health care workers, asking about service provision, taken at baseline (T1), and repeated at T2 and T3 (26)<br>• Surveys of health care workers about disruptions (48, 51, 52)<br>• Surveys of service users (via SMS) asking them to indicate if they received the health service for which they attended the facility (38)<br>• Survey of service users asking them if they missed a health care service and if yes then they are asked to indicate why from a list of facility-related factors (27)	• Surveys of health care workers, asking about service quality, taken at baseline (T1), and repeated at T2 and T3 (26)<br>• Surveys of health care workers about disruptions (52)

TABLE 2: INDICATORS

AUTHOR	LOCATION	DESIGN	MEASURE	POPULATION	INTERVENTION	INDICATOR	EXAMPLE	NOTES
Ashish (39)	Nepal	Observation of clinical services	Extent of disruption	Service-providers	Maternity services	Measured the mean difference of the number of health workers in the labour room and the number of days that maternity services were disrupted between the two time periods (pre-pandemic and pandemic).	e.g. mean number of days (4.3) that maternity services were halted during the pandemic and concluded that the number of health care workers (per 24 hours in the labour and delivery room) had decreased to 5.4 (from 6.2 before the pandemic) health workers due to redeployment to COVID-19 dedicated care.	

For peer review only - http://bmjopen.bmj.com/site/about/guidelines.xhtml

Assefa (14)	Burkina Faso, Ethiopia, Nigeria	Cross-sectional survey	Extent of disruption	Service-providers	General (includes iron and folic acid, ANC, and family planning services)	Measure of the subjective assessment of the categorical level of service disruption presented as a mean interruption score.	e.g. maternal and reproductive services were scored an average of 2.24 out of six [six = total disruption] for Nigeria, 1.72 out of six for Burkina Faso, and 1.67 out of six for Ethiopia	
Avula (29)	India	Cross-sectional survey (asking about T1 and T2) and routine HIS data on service provision (T0)	Extent of disruption	Service-providers	Iron and folic acid, and ANC	Measure of changes in service provision (categorically defined at T1 and T2 as proportion of women provided with ANC services, and at T0 as the number of women receiving services) across the pre-pandemic, lockdown, and post-lockdown periods.	e.g. ANC services were disrupted during the lockdown period but were restored during the post-lockdown period (but not to pre-pandemic levels).	
Nguyen (26)	Bangladesh	Longitudinal survey (at T0, and repeated at T2 [at which time participants asked to recall services provided in the month preceding the survey (T2) and during lockdown (T1)]	Extent of disruption	Service-users and service-providers	Iron and folic acid, and ANC/PNC	Proportionate measure of service provision (defined as the proportion of service-providers indicating provision of specific services, and the proportion of service-users indicating receipt of specific services) at pre-pandemic (T0), lockdown (T1), and post lockdown (T2).	e.g. fewer facilities offered ANC services during lockdown when compared to before the pandemic (dropping 6.6 percentage points), and that there were significant drops in the receipt of anthropometric measurements, and iron/folic acid/calcium supplementation. All services recovered post-lockdown but not to the pre-pandemic levels.	
Rezapour (38)	Iran	Survey	Extent of disruption	Service-users	General (includes midwifery services)	Proportionate measure of services received as a factor of total presentations, by month.	e.g. the study found that there was a small but significant drop in the ASPD for midwifery services when comparing the pandemic to the pre-pandemic periods.	
Saso (51)	51 countries	Cross-sectional survey	Extent of disruption	Service-providers	Maternal and infant vaccination services	Proportionate measure of subjective assessment of disruption to vaccine delivery across several countries.	e.g. 53% of LMIC-based respondents reported disruptions to maternal immunisation services compared to 42% of HIC-based respondents.	

For peer review only - http://bmjopen.bmj.com/site/about/guidelines.xhtml

Semaan (52)	61 countries	Cross-sectional survey	Causes of disruption	Service-providers	Maternal and newborn post-natal care	Proportionate measures of the subjective assessment of the causes of disruption (in terms of the provision and quality of services) to post-natal care across multiple countries.	e.g. respondents from LMICs reported various disruptions due to COVID-19 including reduced numbers of beds due to social distancing (37%), cessation of home visits (20%), and suspension of post-natal care (8%).	The survey categories describing disruption were identified from previous survey rounds.
Tadesse (27)	Ethiopia	Cross-sectional survey	Causes of disruption	Service-users	ANC services	Proportionate measure of disrupted (delayed or missed) ANC visit as well as a proportionate subjective assessment of the causes of the disruption.	e.g. 216 respondents reported missed or late ANC visits, of which 72 (33.33%) attributed this to diversion of maternal services to COVID-19.	It is unclear where the survey categories of disruption have come from.
Villalobos Dintrans (48)	Latin America and Caribbean	Cross-sectional survey	Extent of disruption	Service-providers	General (includes MNH services)	Proportionate measure (using a Likert scale: suspended, partially reduced, same as before, adapted to circumstances, new service created) of the perception of the impact of COVID-19 on health during the pandemic.	e.g. services for newborns and pregnant women —such as institutional delivery care and postpartum care—, as well as vaccination programs, showed the best perceptions of coverage during the pandemic.	

For peer review only - http://bmjopen.bmj.com/site/about/guidelines.xhtml

FIGURE 1: PRISMA flow chart

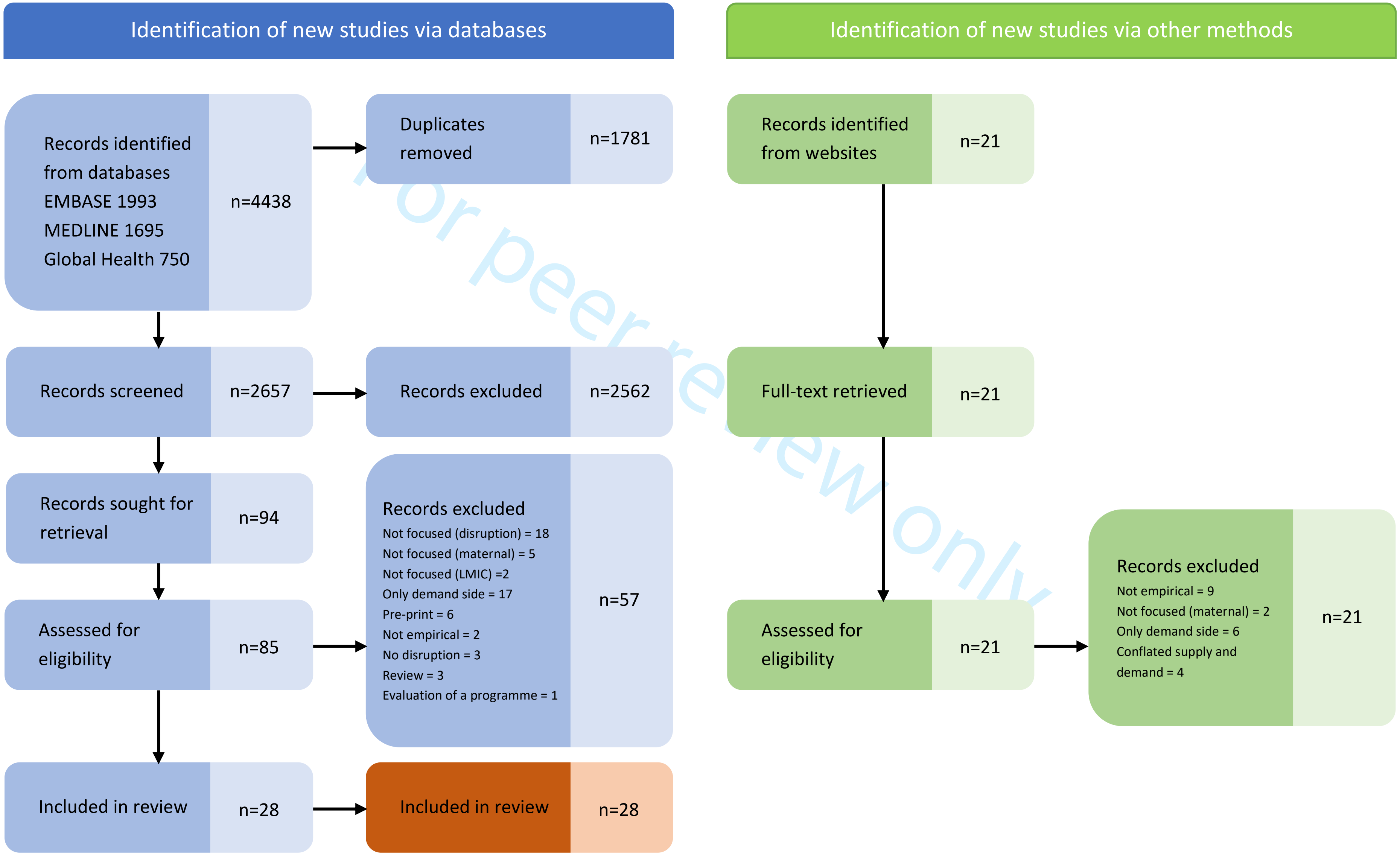

For peer review only - http://bmjopen.bmj.com/site/about/guidelines.xhtml

APPENDIX 1: Search Strategy

Database	MNH	Service	COVID-19
MEDLINE	maternal OR pre?natal OR peri?natal or peri?partum OR ante?natal OR post?natal OR postpartum OR pregnan* OR breastf* OR babo?r OR midwife* OR "birth attendant*" OR "attended birth*" OR new?born [TI/AB]<br><br>maternal health services [MeSH]	service OR program* [TI/AB]	COVID-19 OR 2019-nCoV OR SARS-CoV-2 OR coronavirus OR COVID OR COVID19 [TI/AB]<br><br>covid-19 [MeSH]
EMBASE	maternal OR pre?natal OR peri?natal or peri?partum OR ante?natal OR post?natal OR postpartum OR pregnan* OR breastf* OR babo?r OR midwife* OR "birth attendant*" OR "attended birth*" OR new?born [TI/AB]	service OR program* [TI/AB]	COVID-19 OR 2019-nCoV OR SARS-CoV-2 OR coronavirus OR COVID OR COVID19 [TI/AB]
Global Health	maternal OR pre?natal OR peri?natal or peri?partum OR ante?natal OR post?natal OR postpartum OR pregnan* OR breastf* OR babo?r OR midwife* OR "birth attendant*" OR "attended birth*" OR new?born [TI/AB]	service OR program* [TI/AB]	COVID-19 OR 2019-nCoV OR SARS-CoV-2 OR coronavirus OR COVID OR COVID19 [TI/AB]

For peer review only - http://bmjopen.bmj.com/site/about/guidelines.xhtml

APPENDIX 2: INCLUDED STUDIES

AUTHOR	YEAR	AIM	METHOD	SETTING	DATA COLLECTED	FOCUS	FINDINGS
Adelekan (40)	2021	"…to investigate the extent to which the COVID-19 pandemic and related lockdowns had affected the provision of essential reproductive, maternal, child, and adolescent health (RMCAH) services in primary health care facilities across the Nigerian States" (p. 01).	**QUALITATIVE**<br>Semi-structured interviews (questionnaire not provided)<br>Description of questions: description of the health facility, RMCAH service delivery and use, and difficulties experienced before/during/after lockdown.<br>Head nurses and midwives	**NIGERIA**<br>Primary Health Centres (PHCs) in selected Local Government Areas (LGAs) in 10 states: Lagos, Akwa Ibom, Kano, Kaduna, Gombe, Borno, Ogun, Enugu, Adamawa, and the Federal Capital Territory (FCT) (Abuja Municipal Area Council)	**2020**<br>1 Nov-16 Dec	**RMCAH**	**PROVISION OF SERVICE**<br>**Defined as: Percentage of surveyed PHC's offering a service**<br>ANC: No decline in provision of service except in Gombe (10% decrease during lockdown)<br>Delivery care: No decline in provision of service.<br>Intrapartum care: No decline in provision of service except in Gombe (increased during lockdown but following lockdown dropped to pre-lockdown levels).<br>Postnatal care: Decline in provision of service during lockdown (for some states) and further decline following lockdown (for some states).<br>Childhood immunisation: Decline during lockdown and further decline following lockdown (except in Borno)
Akaba (39)	2022	"…to explore the barriers and facilitators of access to MNCH services during the first wave of COVID-19	**QUALITATIVE**<br>In-depth interviews (questionnaire provided)<br>Used 'three delays model' to structure interviews (i.e. reason for delay in seeking	**NIGERIA**<br>Six states: Abuja, Lagos, Kano (high numbers of cases); Engu, Taraba,	**2020**<br>May/Jul	**MNH**	**QUALITY OF SERVICE**<br>• Delays in receiving care<br>• Shortage of health staff<br>• Inadequate supplies<br>• Lack of preparedness amongst staff

For peer review only - http://bmjopen.bmj.com/site/about/guidelines.xhtml

		pandemic in Nigeria" (p. 01).	care, delay in reaching health care facility, delay in receiving care at the hospital).<br>Service users, health workers, policymakers	Bayelsa (lower numbers of cases)			
Asefa (47)	2022	"…to explore how the COVID-19 pandemic negatively affected frontline health workers' ability to provide respectful maternity care globally" (p. 378).	**QUALITATIVE**<br>Survey data [free-text responses] (questionnaire not provided)<br>Description of question: '*At this time, to what extent do you feel that you are able to provide respectful care to women and newborns compared to before the COVID-19 outbreak?*'<br>Health workers (mainly midwives, obstetricians, gynaecologists, nurses, and GPs)	**71 COUNTRIES**<br>Includes LMICs and HICs	**2020**<br>Jul-Dec	**Respectful maternity care**	**QUALITY OF SERVICE**<br>Six ways in which the provision of care was negatively affected:<br>• Less family involvement<br>• Reduced emotional support to women<br>• Reduced physical support for women<br>• Compromised standards of care<br>• Increased risk of medically unjustified caesarean section<br>• Overwhelmed staff with rapidly changing guidelines and enhanced infection prevention measures
Ashish (37)	2021	"…to understand the disruption of maternity services in Nepal due to the COVID-19 pandemic as well as to explore the provision and experience of institutional delivery care" (p. 2).	**BOTH**<br>**Quantitative**<br>Observation [number of health care workers in the labour and delivery room and those re-deployed to COVID-19 area; number of days maternity services were disrupted]	**NEPAL**	2019/20<br>Mar-Aug 2019, Mar-Aug 2020	**Respectful maternity care**	**PROVISION OF SERVICE**<br>• Maternity services halted for mean 4.3 days during pandemic<br>• Redeployment of staff to COVID-19 care<br>• Positive<br>• Reduced number of health care workers in labour room (compared to pre-COVID)

For peer review only - http://bmjopen.bmj.com/site/about/guidelines.xhtml

	2021		**Qualitative**<br>Semi-structured interviews (questionnaire not provided)<br>Mothers with live births (followed-up 45 days post-partum)				**QUALITY OF SERVICE**<br>• Less than half were counselled to keep newborn warm (40%), or exclusive breastfeeding (44%).<br>• Few were counselled on newborn danger signs (15%)<br>• Consent before vaginal examination (primiparous 55%; nulliparous 44%)
Assefa (13)	2021	"…to characterize the impacts of the COVID-19 pandemic on the interruptions on health services from the perspectives of both HCPs and community members in three sub-Saharan African countries, Burkina Faso, Ethiopia, and Nigeria" (p. 324).	**QUANTITATIVE**<br>Survey data [interruption of health service by type] (questionnaire not included)<br>Questions related to the interruption of a type of health service were scored from 0 to 2 (0 = no interruption;1 = partial interruption; 2 = complete interruption). Asked to score: ANC, iron and folate, family planning.<br>Health care workers (also surveyed community to assess access – not included)	**BURKINA FASO, ETHIOPIA, NIGERIA**	**2020**<br>Jul-Nov	**General (but includes maternal and reproductive health services)**	**PROVISION OF SERVICE**<br>• Almost all services were reported to have experienced disruption<br>• Maternal and reproductive services particularly interrupted<br>• MRH: Nigeria (2.24 out of 6); Burkina Faso (1.72 out of 6); and Ethiopia (1.67 out of 6). [where 6=complete interruption]. Total (1.85 out of 6).<br>• Total health service interruption: Nigeria (7.9 out of 20); Burkina Faso (6.2 out of 20); and Ethiopia (5.4 out of 20). [where 20=complete interruption]. Total (6.49 out of 20)
Avula (27)	2022	to, "…stud[y] disruptions and restorations, challenges and adaptations in health and nutrition service delivery by frontline workers (FLWs) in India during	**QUANTITATIVE**<br>Survey data [service delivery at T1-lockdown and T2-post-lockdown] (questionnaire included)<br>Question example:	**INDIA**<br>Seven states: Bihar, Chhattisgarh, Madya Pradesh, Odisha, Tamil Nadu, Telangana, and Uttar Pradesh	**2020**<br>Aug-Oct	**FLW-provided services (iron and folic acid, and ANC)**	**PROVISION OF SERVICE**<br>• Services were disrupted during T1-lockdown but were restored in the T2-post lockdown (but not to T0-pre-pandemic levels)

For peer review only

		COVID-19 in 2020" (p. 1).	a. "Were you able to provide help in providing antenatal care services to all pregnant women?" (Yes/No)<br>b. "…in the last month?" (0=No one; 1=Yes, only to some pregnant women in the last trimester for high-risk pregnant women; 2=Yes, to all pregnant women)<br>c. "…during the last COVID-19 lockdown in April?"<br>HIS data also retrieved to establish T0-pre-COVID-19 service provision.<br>Frontline workers				
Basnet (38)	2022	"…to explore the experiences of nurses providing maternity care in the public sector during the COVID-19 pandemic in Nepal" (p. 01).	**QUALITATIVE**<br>Semi-structured interviews (topic guide not provided)<br>Topic guide was developed based on previous relevant literature, the research question, and informal interviews.<br>Nurses	**NEPAL**<br>Biratnagar (Koshi Hospital)	**2020**<br>Sep-Nov	**MNH**	**QUALITY OF SERVICE**<br>• The majority of the 10 participants had experienced decreased quality of care<br>• Decrease in nurse-patient time<br>• Decrease in presence of medical practitioners<br>• Time allocated per patient decreased<br>**PROVISION OF SERVICE**<br>• Reduced kangaroo mother care for pre-term<br>• Counselling less consistent
Hazfiarini (33)	2022	"…[t]o explore how COVID-19 influenced the provision of high-	**QUALITATIVE**<br>Semi-structured interviews (topic guide not provided)	**INDONESIA**<br>Surabaya and Mataram	**2020/1**	**Midwife services**	**PROVISION OF SERVICE**<br>• Cancelled education classes

For peer review only - http://bmjopen.bmj.com/site/about/guidelines.xhtml

		quality maternity care in Indonesia" (p. 01).	Topic guide included three sections: midwives were asked how they provided antenatal, childbirth, postpartum care prior to COVID-19 [Q1] and during COVID-19 [Q2], and were asked to describe experiences providing care during COVID-19 [Q3].<br>Midwives		Dec 2020 - Feb 2021		• Reduced frequency of ANC reduced (reducing from 6 to 2-4)<br>• Reduced post-partum visits
Leung (41)	2022	"…to explore maternity healthcare workers' experiences of, and perceptions about providing maternity care during the COVID-19 outbreak in Lagos State, Nigeria" (p. 01).	**QUALITATIVE**<br>Semi-structured interviews (topic guide provided)<br>Topic guide included:<br>"How has COVID-19 outbreak affected maternity care services?<br>What impact do you feel that COVID-19 pandemic has had on your work?<br>Probe: How has COVID-19 affected normal daily tasks/responsibilities?<br>Probe: How has COVID-19 affected the way you look after patients?<br>Probe: Have supply of drugs, equipment, PPE been affected?	**NIGERIA**<br>Seven Local Government Areas in Lagos State (Lagos Mainland, Surulere, Ikeja, Alimosho, Kosofe, Badagry, OshodiIsolo)	**2021**<br>Mar-Apr	**Facility-based maternity care**	**PROVISION OF SERVICE**<br>• Antenatal appointments for non-urgent cases had been reduced<br>• Home visits not possible<br>• Some felt services maintained<br>**QUALITY OF SERVICE**<br>• Some midwives had taken on COVID-19 roles (e.g. vaccination)<br>• Reduced capacity of consultation room

For peer review only - http://bmjopen.bmj.com/site/about/guidelines.xhtml

			Probe: Have staff been redeployed from or within your facility?<br>Please tell me about any changes COVID-19 has had on your work<br>Probe: Can you explain why there have been these changes?<br>Probe: How do you feel about the changes you have had to make during the pandemic?<br>Probe: COVID-19 control measures - How do you feel about mask wearing/social distancing/handwashing?<br>What differences have you noticed in the way that patients interact with your services during COVID-19?<br>Probe: Has there been a change? Why?<br>Midwives and TBAs				
Mirzakhani (35)	2022	"…to explore the experiences of women with high-risk pregnancies who were receiving prenatal care during the COVID-19 pandemic" (p. 01).	**QUALITATIVE**<br>Semi-structured interviews (no topic guide provided)<br>Topic guide included:<br>1. What was your experience with prenatal care during the Corona pandemic?	**IRAN**<br>Ghaem, Ommolbanin, and Imam Reza in Mashhad	**2020/2**<br>Sep 2020 – Mar 2021	**Women with high-risk pregnancies**	**QUALITY OF SERVICE**<br>• Health care provider's care and attention had decreased during COVID-19<br>• Staff refused to listen to foetal sounds or perform abdominal examinations<br>• Shorter visits from health care workers

For peer review only - http://bmjopen.bmj.com/site/about/guidelines.xhtml

			2. How do you feel about being pregnant in this situation?<br>3. How do you feel when you apply for prenatal care?<br>4. How is your access to prenatal care in the face of the Corona pandemic?<br>5. Discuss the factors that make you feel good about receiving health services.<br>6. Discuss the factors that make you feel uneasy/bad about receiving health services.<br>7. How does the environment and location of health delivery affect health services receipt?<br>8. What limitations /restrictions did you face when seeking health services?<br>Women with high-risk pregnancies				• Staff not taking time to talk<br>• Mothers reported stress, anxiety, neglect<br>**PROVISION OF SERVICE**<br>• Some doctors closed their offices<br>• Drug shortages
Mohammed (42)	2022	"…[to] assess the effect of Covid-19 on maternal and child health services in Federal medical Centre Gusau Zamfara state following the lockdown" (p. 124).	**QUALITATIVE**<br>Key informant interviews (no topic guide provided)<br>Topic guide was 'standard COVID-19 KII guide' and asked respondents' opinion on the COVID-19 outbreak	**NIGERIA**<br>Federal medical centre Gusau, Zamfara state	??	**MNH services**	**PROVISION OF SERVICE**<br>• Some respondents reported having to quarantine after contact with COVID-19 positive patient<br>• Most participants believed services were affected with some suspended

For peer review only - http://bmjopen.bmj.com/site/about/guidelines.xhtml

			and its specific and general effects on the facility.<br>Heads of units delivering MNCH services (principal medical officers, chief nursing officers, principal nursing officers and nutrition officers)				**QUALITY OF SERVICE**<br>• Paediatric outpatient and paediatric emergency unit reduced staffing numbers
Musiimenta (43)	2022	"…[to] describe the challenges experienced in accessing maternal and child health services by women with limited or no education during this COVID-19 pandemic and discuss the potential of social network technologies (SNTs) to support maternal and child health amidst this crisis".	**QUALITATIVE**<br>Interviews (no topic guide provided)<br>Interviews meant to elicit information about challenges women encountering in accessing maternal and child health services during lockdown.<br>Women who had recently given birth in a rural setting	**UGANDA**<br>Mbarara Regional Referral Hospital	**2021**<br>Jul	**Post-natal care**	**PROVISION OF SERVICE**<br>• Delays in accessing services, long queues and missed appointments
Nakate (44)	2022	"…[to] explore the experiences of mothers and their significant others of comprehensive care in the first 1000 days of life post-conception during the COVID-19 pandemic" (p. 01).	**QUALITATIVE**<br>Semi-structured interviews (no topic guide provided)<br>Topics included:<br>Open-ended questions regarding participants' experiences accessing care and their understanding of these experiences.	**UGANDA**<br>Bunghokho-Mutoto sub-county, Mbale District	**2020**<br>10 Oct-9 Nov	**First 1000 days post-conception**	**PROVISION OF SERVICE**<br>• Lack of follow-up visits<br>• Curfew resulted in staff starting late and leaving early whilst mothers waited<br>• Facilities reduced admissions or appointments due to staff shortages<br>**QUALITY OF SERVICE**

For peer review only - http://bmjopen.bmj.com/site/about/guidelines.xhtml

			Mothers (pregnant or with a child under 2 years) and their significant others				• Shortages of staff and work overload was felt to have hindered provision of care<br>• Staff pre-occupied with infection prevention and control
Nguyen (24)	2021	"…to (1) quantify the effect of the COVID-19 pandemic and COVID-19 restrictions on health and nutrition service provision and utilisation by pregnant women and mothers of children under 2 years of age in Dhaka City, Bangladesh, and (2) identify adaptions and a range of feasible solutions that have the potential to strengthen delivery and uptake of essential health and nutrition interventions in the context of COVID-19 and beyond" (p. 03).	**QUANTITATIVE**<br>Survey data [to assess survey provision before pandemic, during lockdown, and post-lockdown]<br>(survey not included)<br>Survey (health providers):<br>1) Feb baseline: assess exposure to nutrition training, workload and time commitments, and services provided.<br>2) Sep/Oct follow-up: assess trainings, receipt of PPE, additional responsibilities, service provision, and challenges and adaptations (asked to recall Mar-May [lockdown] and Sept)<br>Survey (mothers):<br>1) baseline: assess receipt of health and nutrition services<br>2) follow-up: how service use had changed, exposure to services, counselling messages, mode of service receipt and challenges	**BANGLADESH**<br>Dhaka City	**2020**<br>Sept-Oct	**Mothers with children <2**	**PROVISION OF SERVICE**<br>• Fewer facilities offered ANC services during lockdown when compared to before pandemic (dropping 6.6 percentage points)<br>**QUALITY OF SERVICE**<br>• Most ANC services still offered (with the exception of anthropometric measurements (20 percentage point drop for weight measurements), (29 percentage point drop for height measurements)<br>• Post-lockdown anthropometric measurements were higher post-lockdown but still below pre-pandemic levels<br>• Smaller drops reported for iron folic-acid and calcium supplementation (<4 percentage points), nutrition counselling (dropped to 12.5 percentage points post-lockdown when compared to lockdown)<br>• Other services affected but all improved post-lockdown though not to pre-pandemic levels

For peer review only

For peer review only - http://bmjopen.bmj.com/site/about/guidelines.xhtml

			**Included a baseline survey**<br>Health providers and mothers with children under 2				• Increased workloads during the pandemic was reported by 56% of providers.
Padhye (28)	2022	"…to understand the status of maternal health service provision and challenges faced by 110 pregnant and recently delivered women, 38 health care providers and 18 Village Health Sanitation and Nutrition Committee members during COVID-19 pandemic" (p. 01).	**QUALITATIVE**<br>Interviews<br>(topic guide not included)<br>Interviews were intended to: understand the effect of COVID-19 and lockdown on service provision (for health care providers) and understand the role and capacity building efforts for providing access to maternal health services (VHSNC) in their village.<br>Health care providers and Village Health Sanitation and Nutrition Committee members (VHSNC)	**INDIA**<br>Two districts in Assam (Kamrup and Darrang)	**2020**<br>Oct-Dec	**Maternal health services**	**PROVISION OF SERVICE**<br>• Most ANC/PNC services were managed through home visits but travel to remote areas was difficult<br>• Non-availability of laboratory services was a significant gap in ANC provisioning<br>• Village-level health centres were closed for three months during lockdown<br>• Post-lockdown iron and folic acid tablets were unavailable at the village health centres<br>• Village Accredited Social Health Activists reported not being able to provide any of the expected health services during the pandemic<br>• Services that were not available included ultrasound check-up, laboratory services, health care providers, and certain health facilities<br>**QUALITY OF SERVICES**<br>• Home deliveries for high-risk women were supported by health care staff but without backup

For peer review only - http://bmjopen.bmj.com/site/about/guidelines.xhtml

							support from a medical officer or an auxiliary nurse midwife
PSBI Formative Research Study Group (45)	2022	"…to understand the pandemic's impact on barriers to and mitigation for strategies of care-seeking and managing possible serious bacterial infection (PSBI) in young infants" (p. 01).	**QUALITATIVE**<br>Observation, in-depth interviews, and focus groups (to ascertain trends in service provision and use across lockdown and post lockdown periods)<br>(conceptual framework included)<br>Conceptual framework lists factors influencing PSBI management:<br>**Changes in National and local policies**<br>• Conversion of hospitals into COVID-19 care centres<br>**COVID-19 care centres**<br>• Resource reallocation • Task shifting • Diversion of cases to other higher or lower health care facilities.<br>**Health Systems changes: public and private; facility and community**<br>• Functionality of outpatient and inpatient facilities • Effective referral system • Availability of human resources • Availability of essential equipment,	**BANGLADESH, ETHIOPIA, INDIA, NIGERIA, PAKISTAN**	**2020/1**<br>Dec 2020 – Mar 2021	**Newborn care**	**PROVISION OF SERVICE**<br>• Several hospitals were closed, offered restricted services, or converted to COVID-19 care centres<br>• Respondents discouraged the admission of infants except in an emergency<br>• Many of these problems reported to have existed pre-COVID-19<br>• Community clinics closed during lockdown<br>• Routine services (e.g. ANC, vaccination, and post-partum care) were disrupted<br>• Outpatient care was disrupted at many sites due to service suspension, limited hours, or non-availability of staff<br>• Home-based post-natal and referral were also reduced<br>**QUALITY OF SERVICES**<br>• Some facility staff indicated that staff shortages due to COVID-19, insufficient oxygen and medicines, and unhygienic conditions at facilities had led to sub-optimal care for young infants<br>• Some respondents reported poor care and limited patient time with staff who were unwilling to touch or examine infants

For peer review only - http://bmjopen.bmj.com/site/about/guidelines.xhtml

			supplies and medicines • Perceptions, fear and apprehensions among health care providers and community health workers • Availability of COVID-related supplies<br>**Caregivers and Community**<br>• Fear of accessing care • Needs for seeking treatment outside the home or allowing health personnel to visit home • Constraints in accessing treatment • Transportation availability, costs • Perceived barriers • Immediate competing demands • Economic stress, loss of employment, PSBI low priority<br>Health care staff (CHWs, staff nurses, medical officers, program managers, and private practitioners)				• Many of these problems reported to have existed pre-COVID-19<br>• Social distancing felt to have reduced service quality
Rao (48)	2021	"…to survey reported disruptions to small and sick newborn care worldwide and undertake thematic analysis of healthcare providers' experiences and proposed mitigation strategies to provide insights on	**QUALITATIVE**<br>Survey data<br>(survey not included)<br>Survey collected free-text data on:<br>Demographic characteristics, healthcare preparedness and response to COVID-19, the effect of COVID-19 on	**62 COUNTRIES**<br>mainly LMICs	**2020**<br>13 Jul – 13 Oct	**Newborn care**	**QUALITY OF SERVICES**<br>• Changes to newborn care reported including reallocation of unit space and reassignment of staff from newborn care to COVID-19 and other duties<br>• Oxygen supplies for newborns compromised<br>**PROVISION OF SERVICES**

For peer review only - http://bmjopen.bmj.com/site/about/guidelines.xhtml

		disruptions to coverage and quality of small and sick newborn care, and to identify possible solutions to protect vulnerable newborns during the COVID-19 and similar future pandemics" (p. 1).	health professionals and newborn care, and major disruptions and solutions.<br>Health providers (nurses, midwives, doctors and community health workers), public health professionals and policymakers				• Some respondents reported closure of wards (in this case KMC) due to its proximity to the COVID-19 area<br>• A hospital outbreak resulted in closure of 'mother lodger' and KMC wards<br>• Limited social distancing space affected the quality of neonatal care<br>• Fewer staff to carry out follow-up visits and fewer appointments per newborn<br>• Staff anxiety about infection compromised KMC care
Rezapour (36)	2022	"…[to] investigate the impact of the COVID-19 pandemic on the service utilization status in the Iranian PHC system" (p. 01).	**QUANTITATIVE**<br>Survey of patients<br>(text message included)<br>Indicator Actual Percentage of Service Delivery (ASPD) measured the actual number of people who have gone to PHC facilities and received the service.<br>SMS was sent to all patients via SMS. SMS message read: "Has the service been received?"<br>**Includes baseline survey**<br>PHC facility patients	**IRAN**	**2019-2021**<br>Mar 2019-Jan 2020 (before); Feb 2020-Feb 2021 (after)	**General (include midwifery services)**	**PROVISION OF SERVICES**<br>• There was a significant difference in ASPD for midwifery services when comparing pre-pandemic to the COVID-19 period
Sahoo (29)	2022	"…[to] explore the experiences of	**QUALITATIVE**	**INDIA**	**2020**	**ANC, nutritional**	**PROVISION OF SERVICES**

For peer review only - http://bmjopen.bmj.com/site/about/guidelines.xhtml

		vulnerable communities—urban-slum-dwelling women regarding maternal and child health services during COVID-19" (p. 01).	In-depth interviews [about experiences including coping strategies, and health system readiness concerning participants' MCH care]<br>(topic guide included)<br>Topic guide questions:<br>Q1. Please share your experience during the COVID-19 pandemic?<br>Q2. What is your experience with availing antenatal services/Intra/postnatal services/immunization services during a regular period in your community? Probe: availability—site, day, time, staff, logistics, transport, appropriateness—technical and professional adequacy, approachability, acceptability, affordability, continuity of services<br>Q3. What changes have you seen in services during the COVID-19 situation? Probe: availability, appropriateness, approachability, acceptability, affordability, continuity of services<br>Q4. How did you manage the care of your sick infants during the pandemic?	Four states: Odisha, Uttarakhand, Chhattisgarh, and Assam.	Apr-May	**care for pregnant women, intra-natal and post-natal care, immunisation, and sick infant treatment**	• The majority of respondents reported that they received the antenatal check-up whilst in lockdown<br>• Some intra-natal services were not provided by the facility<br>• Timing of immunisation services were changed to accommodate staff COVID-19 duties

For peer review only - http://bmjopen.bmj.com/site/about/guidelines.xhtml

			Q5. How did you cope with the situation?<br>Q6. In your view, how can we strengthen the services in an emergency?<br>Urban slum dwelling women who were antenatal, intra-natal, or postnatal				
Saso (49)	2020	"…[t]o understand immunization service interruptions specifically for maternal, neonatal and infant vaccines" (p. 01).	**BOTH**<br>Survey data<br>(survey question included)<br>Survey questions:<br>Are you aware of any issues in delivering these vaccines successfully during the COVID-19 pandemic? (Yes/No/Don't know)<br>If you answered yes to the above question, please provide some detail below (e.g. administration problems, rescheduling, staff shortage, parental concerns, social/logistical barriers, others)<br>Health care workers (e.g. doctors, midwives, nurses, lab scientists, public health officials)	**51 COUNTRIES**<br>Includes 32 (63%) LMICs	**2020**<br>15 Apr-30 Apr	**Maternal and infant vaccination**	**PROVISION OF SERVICE**<br>• 50% of respondents reported disruption in either maternal or infant vaccine delivery (similarly reported across HICs and LMICs)<br>• Disruption newborn vaccination was reported by 42% (n=15) of LMIC-based respondents<br>• Disruption in maternal vaccination was reported by 53% (n=19)<br>• Disruption to clinic services was reported by most participants<br>• Issues secondary to vaccine shortages and logistics primarily described within LMICs<br>**QUALITY OF SERVICE**<br>• Staff shortages were described more frequently in the context of newborn, infant, toddler vaccination<br>• The barriers described in LMICs were more skewed towards supply-side issues
Semaan (50)	2022	"…to explore PNC provision, availability,	**QUANTITATIVE**	**61 COUNTRIES**	**2020**	**Post-natal care (mothers**	**QUALITY OF SERVICE**

For peer review only - http://bmjopen.bmj.com/site/about/guidelines.xhtml

		content and quality following the early phase of the COVID-19 pandemic" (p. 01).	Survey data (questions developed iteratively from past surveys)<br>(survey questions provided)<br>Survey questions:<br>In the past month, how was inpatient postnatal care for women and newborns affected? (Please select all that apply)<br>In the past month, how was outpatient postnatal care affected? (Please select all that apply)<br>In the past month, was the content of inpatient postnatal care to women and newborns provided by you or in your facility affected in terms of: (Please select all that apply)<br>In the past month, was the content of outpatient postnatal care to women and newborns provided by you or in your facility affected in terms of: (Please select all that apply)<br>Maternal and newborn health providers	Includes 34 LMICs	Jul-Dec	**and newborns)**	• Amongst LMIC respondents 37% reported reduced numbers of beds due to social distancing<br>• Amongst LMIC respondents 14% reported reduced space in postnatal ward<br>**PROVISION OF SERVICE**<br>• Amongst LMIC respondents 53% reported that appointments were scheduled further apart<br>• Amongst LMIC respondents 28% reported not being able to provide postnatal care face-to-face for all patients<br>• Amongst LMIC respondents 20 reported that home visits had stopped<br>• Amongst LMIC respondents 8% reported suspension of outpatient postnatal care<br>• Amongst LMIC respondents 22% reported shorter operating hours or fewer days for postnatal care<br>• Amongst LMIC respondents 6% reported suspending inpatient postnatal care
Shrivastava (30)	2021	"...to understand the challenges faced by pregnant women	**MEDIA ANALYSIS**<br>Content analysis (News articles reporting on the	INDIA	2020	Pregnant women	**PROVISION OF SERVICE**

For peer review only - http://bmjopen.bmj.com/site/about/guidelines.xhtml

		seeking institutional care during the lockdown" (p. 202).	challenges that pregnant women faced when seeking institutional care during the lockdown)<br>Pregnant women seeking institutional care	17 states	Mar-May		• Night shift staff were reassigned to other duties to address HR gaps caused by COVID-19<br>**QUALITY OF SERVICE**<br>• Pregnant women experienced poor quality care during lockdown; related to systemic health system issues, or individual care
Sinha (31)	2022	"…[t]o estimate utilization of maternal, perinatal healthcare services after the lockdown was implemented in response to the COVID-19 pandemic compared to the period before" (p. 01).	**QUALITATIVE**<br>Semi-structured survey and in-depth interviews with a sub-sample<br>(no survey questions or topic guide provided)<br>Survey included:<br>Sociodemographic, antenatal, intrapartum and postnatal care details including out-of-pocket expenditure<br>Interview topics:<br>Experiences, challenges, and barriers in accessing health care services.<br>Women who had recently delivered	INDIA<br>Delhi	2019/20<br>Oct 2019 – Mar 2020 (pre-lockdown)<br>Mar 2020 – Nov 2020 (after lockdown)	Maternal and perinatal services	**PROVISION OF SERVICE**<br>• Many hospitals had been converted into CVODI-19 care centres<br>• Respondents reported that community workers were unavailable during lockdown for AND or postnatal services<br>**QUALITY OF SERVICE**<br>ANC (survey)<br>• The most common barriers to availing ANC services were poor quality of care (55%), lack of transport (41%), or long waiting times (39%).<br>ANC (interviews)<br>• Accounts of poor quality care and staff reluctant to carry out examinations<br>Intrapartum care (survey)<br>• 28% of respondents reported issues with intrapartum care<br>Intrapartum care (interviews)

For peer review only - http://bmjopen.bmj.com/site/about/guidelines.xhtml

							• Accounts of fear of contracting COVID-19, poor quality of care, and availability of few or no health staff<br>• Respondents found labour room experience distressing due to shortage of staff and unhygienic facilities
Tadesse (25)	2020	"…[t]o assess the impact of the COVID-19 pandemic on antenatal care utilization among pregnant women attending public facilities in Northeast Ethiopia" (p. 1181).	**QUANTITATIVE**<br>Interviewer-administered questionnaire<br>(questionnaire provided)<br>Did you miss or late to start ANC visit during COVID-19 outbreak? (Yes/No)<br>Facility related factors:<br>1) deploying of maternal care workers (Yes/No)<br>2) interruption and diversion of maternity services to COVID-19 response (Yes/No)<br>3) Fear of COVID-19 infection (Yes/No)<br>4) Social distancing (Yes/No)<br>Pregnant women	**ETHIOPIA**	**2020**<br>Feb-Aug	**ANC**	**PROVISION OF SERVICE**<br>• Interruption and diversion of maternal health services due to COVID-19 reduced full service use by 32%
Tilahun (26)	2022	"…to examine the effect of the COVID-19 pandemic on MNCH service uptake and to identify effective strategies to ensure MNCH service delivery	**QUALITATIVE**<br>In depth interviews<br>(no interview guide; no description of interview guide)	**ETHIOPIA**	**2020**	**Maternal and child health services**	**PROVISION OF SERVICE**<br>• Respondents reported low commitment of health care workers, movement restrictions, and shortage of resources were

For peer review only - http://bmjopen.bmj.com/site/about/guidelines.xhtml

		during the pandemic in Ethiopia" (p. 2782).	Decision-makers, health workers, NGO delegates and clients				felt to have reduced the quality of services
Ulaganeethi (32)	2021	"…to assess the proportion of pregnant women who had not completed the ideal number of antenatal visits, availability of iron-folic acid (IFA) supplements and challenges in availing health services during the period of lockdown" (p. i23).	**QUALITATIVE**<br>Semi-structured interviews (Interview guide provided)<br>Interviews included:<br>3. Did you face any difficulty in availing antenatal care services or healthcare services for any danger sign you noticed during this lockdown? Can you […] your experience?<br>4. Why you were not able avail the antenatal services? (Probe: non-availability of transportation facilities, fear of visiting the hospital, anxiety to go out of house, family support, getting medicines, fear of getting COVID, spread of COVID to child, delivery, income).<br>Pregnant women	**INDIA**<br><br>Puducherry	2020<br><br>Jul-Jun	ANC	**PROVISION OF SERVICE**<br>• Respondents indicated that health facilities closed down their outpatient clinics during lockdown
Villalobos Dintrans (46)	2021	"…[t]o describe the perception of key actors regarding the interruption of health services for populations not prioritized by the pandemic —pregnant women, newborns,	**QUANTITATIVE**<br>Survey (survey questions provided)<br>Question:<br>For the following health actions for pregnant women, newborns, children,	**19 Latin American and Caribbean countries**	2020	General (includes MNH services)	**PROVISION OF SERVICE**<br>Services for newborns and pregnant women, such as institutional delivery care and postpartum care, as well as vaccination programs, showed the

For peer review only - http://bmjopen.bmj.com/site/about/guidelines.xhtml

		children, adolescents, and women— in Latin American and Caribbean (LAC) countries during the first stage of the COVID-19 pandemic" (p. 01).	adolescents and women, at a general level, indicate the state in your country during the month of June 2021. [have been maintained, have been partially reduced, have been suspended, Adapted to new circumstances, created new services, don't know].<br>Decision-makers and implementers of social and health programs and policies				best perceptions of coverage during the pandemic.
Widiasih (34)	2021	"…to explore the experiences of pregnant women regarding ANC and monitoring fetal wellbeing during the COVID-19 pandemic" (p. 01).	**QUALITATIVE**<br>Semi-structured interview<br>(no interview guide provided)<br>Interviews described as:<br>"…covering the pregnant woman's experience when engaged in ANC and how and when they undertook fetal welfare checks during the COVID-19 pandemic".<br>Pregnant women	INDONESIA	2021<br>Jan	Self-foetal wellbeing monitoring and ANC	**PROVISION OF SERVICE**<br>• Respondents reported queues to access care, limits on the number of patients, and changes to service hours

For peer review only

PRISMA 2020 Checklist

Section and Topic	Item #	Checklist item	Location where item is reported
**TITLE**			
Title	1	Identify the report as a systematic review.	TITLE
**ABSTRACT**			
Abstract	2	See the PRISMA 2020 for Abstracts checklist.	p. 2
**INTRODUCTION**			
Rationale	3	Describe the rationale for the review in the context of existing knowledge.	p. 7
Objectives	4	Provide an explicit statement of the objective(s) or question(s) the review addresses.	p. 7
**METHODS**			
Eligibility criteria	5	Specify the inclusion and exclusion criteria for the review and how studies were grouped for the syntheses.	p. 8
Information sources	6	Specify all databases, registers, websites, organisations, reference lists and other sources searched or consulted to identify studies. Specify the date when each source was last searched or consulted.	p. 8-9
Search strategy	7	Present the full search strategies for all databases, registers and websites, including any filters and limits used.	Supplemental
Selection process	8	Specify the methods used to decide whether a study met the inclusion criteria of the review, including how many reviewers screened each record and each report retrieved, whether they worked independently, and if applicable, details of automation tools used in the process.	p. 9
Data collection process	9	Specify the methods used to collect data from reports, including how many reviewers collected data from each report, whether they worked independently, any processes for obtaining or confirming data from study investigators, and if applicable, details of automation tools used in the process.	p. 9
Data items	10a	List and define all outcomes for which data were sought. Specify whether all results that were compatible with each outcome domain in each study were sought (e.g. for all measures, time points, analyses), and if not, the methods used to decide which results to collect.	p. 9
	10b	List and define all other variables for which data were sought (e.g. participant and intervention characteristics, funding sources). Describe any assumptions made about any missing or unclear information.	p. 9
Study risk of bias assessment	11	Specify the methods used to assess risk of bias in the included studies, including details of the tool(s) used, how many reviewers assessed each study and whether they worked independently, and if applicable, details of automation tools used in the process.	p. 9
Effect measures	12	Specify for each outcome the effect measure(s) (e.g. risk ratio, mean difference) used in the synthesis or presentation of results.	n/a
Synthesis methods	13a	Describe the processes used to decide which studies were eligible for each synthesis (e.g. tabulating the study intervention characteristics and comparing against the planned groups for each synthesis (item #5)).	p. 9
	13b	Describe any methods required to prepare the data for presentation or synthesis, such as handling of missing summary statistics, or data conversions.	p. 9
	13c	Describe any methods used to tabulate or visually display results of individual studies and syntheses.	See tables
	13d	Describe any methods used to synthesize results and provide a rationale for the choice(s). If meta-analysis was performed, describe the model(s), method(s) to identify the presence and extent of statistical heterogeneity, and software package(s) used.	p. 9
	13e	Describe any methods used to explore possible causes of heterogeneity among study results (e.g. subgroup analysis, meta-regression).	n/a
	13f	Describe any sensitivity analyses conducted to assess robustness of the synthesized results.	n/a
Reporting bias assessment	14	Describe any methods used to assess risk of bias due to missing results in a synthesis (arising from reporting biases).	n/a
Certainty assessment	15	Describe any methods used to assess certainty (or confidence) in the body of evidence for an outcome.	n/a

For peer review only - http://bmjopen.bmj.com/site/about/guidelines.xhtml

PRISMA 2020 Checklist

Section and Topic	Item #	Checklist item	Location where item is reported
**RESULTS**			
Study selection	16a	Describe the results of the search and selection process, from the number of records identified in the search to the number of studies included in the review, ideally using a flow diagram.	p. 9-10
	16b	Cite studies that might appear to meet the inclusion criteria, but which were excluded, and explain why they were excluded.	Figure 1 and p. 15
Study characteristics	17	Cite each included study and present its characteristics.	Tables 1 and 2
Risk of bias in studies	18	Present assessments of risk of bias for each included study.	n/a
Results of individual studies	19	For all outcomes, present, for each study: (a) summary statistics for each group (where appropriate) and (b) an effect estimate and its precision (e.g. confidence/credible interval), ideally using structured tables or plots.	n/a
Results of syntheses	20a	For each synthesis, briefly summarise the characteristics and risk of bias among contributing studies.	n/a
	20b	Present results of all statistical syntheses conducted. If meta-analysis was done, present for each the summary estimate and its precision (e.g. confidence/credible interval) and measures of statistical heterogeneity. If comparing groups, describe the direction of the effect.	n/a
	20c	Present results of all investigations of possible causes of heterogeneity among study results.	n/a
	20d	Present results of all sensitivity analyses conducted to assess the robustness of the synthesized results.	n/a
Reporting biases	21	Present assessments of risk of bias due to missing results (arising from reporting biases) for each synthesis assessed.	n/a
Certainty of evidence	22	Present assessments of certainty (or confidence) in the body of evidence for each outcome assessed.	n/a
**DISCUSSION**			
Discussion	23a	Provide a general interpretation of the results in the context of other evidence.	p. 14-16
	23b	Discuss any limitations of the evidence included in the review.	p. 16
	23c	Discuss any limitations of the review processes used.	p. 16
	23d	Discuss implications of the results for practice, policy, and future research.	p. 14-17
**OTHER INFORMATION**			
Registration and protocol	24a	Provide registration information for the review, including register name and registration number, or state that the review was not registered.	p. 17
	24b	Indicate where the review protocol can be accessed, or state that a protocol was not prepared.	n/a
	24c	Describe and explain any amendments to information provided at registration or in the protocol.	n/a
Support	25	Describe sources of financial or non-financial support for the review, and the role of the funders or sponsors in the review.	p. 17
Competing interests	26	Declare any competing interests of review authors.	p. 17
Availability of data, code and other materials	27	Report which of the following are publicly available and where they can be found: template data collection forms; data extracted from included studies; data used for all analyses; analytic code; any other materials used in the review.	p. 17

For peer review only
